# Spatiotemporal Targeting Randle Cycle and Immune Checkpoint for Potent Antitumor Therapy

**DOI:** 10.1002/advs.76109

**Published:** 2026-06-15

**Authors:** Yuan Gao, Zijian Gong, Yixuan Fu, Jianan Zheng, Xiao Sang, Binglin Chen, Qinzhi Su, Weiping Gao, Fei Duan, Jinqi Wei, Xuliang Deng, Xinyu Liu

**Affiliations:** ^1^ Central Laboratory NMPA Key Laboratory For Dental Materials National Engineering Research Center of Oral Biomaterials and Digital Medical Devices Beijing Laboratory of Biomedical Materials Beijing Key Laboratory of Biomaterials for Oral Disease National Center For Stomatology National Clinical Research Center For Oral Diseases Peking University School and Hospital of Stomatology Beijing P. R. China; ^2^ Institute of Advanced Clinical Medicine Peking University Beijing P. R. China; ^3^ Department of Nanomedicine Translational Medicine Research Center & Shanghai Key Laboratory of Nautical Medicine and Translation of Drugs and Medical Devices Naval Medical University Shanghai P. R. China; ^4^ First Clinical Division Peking University School and Hospital of Stomatology Beijing P. R. China; ^5^ Department of Geriatric Dentistry Peking University School and Hospital of Stomatology Beijing P. R. China

**Keywords:** glucose oxidase, immunotherapy, nanogels, oral cancer, randle cycle, tumor metabolism

## Abstract

Tumor metabolic reprogramming plays a crucial role in cancer progression and therapeutic resistance. The competitive and compensatory relationship between glucose and lipid metabolism—known as the Randle cycle—poses a major challenge to single‐pathway metabolic inhibition strategies. In this study, we developed a glucose oxidase‐based nanogel (GOX‐NG) system using catechol‐functionalized alginate, which exhibits enhanced tumor penetration, prolonged retention, and sustained glucose depletion in the tumor microenvironment. When combined with etomoxir (ETX), a fatty acid oxidation (FAO) inhibitor, this system effectively implements dual metabolic suppression, thereby enhancing reactive oxygen species (ROS)‐induced immunogenic cell death and reprogramming the tumor immune microenvironment. Further combination with the immune checkpoint inhibitor αPD‐1 amplified antitumor immune responses, achieving complete tumor regression in 60% of animals and full survival in 100% of tumor‐bearing mice. This strategy demonstrates significant potential in the treatment of metastatic tumors through a synergistic starvation–oxidation–immunotherapy loop and paves the way for applying intratumorally‐retaining nanogels to a broad spectrum of therapeutic proteins targeting metabolic pathways.

## Graphical abstract

A glucose oxidase‐based nanogel (GOX‐NG) system using catechol‐functionalized alginate, exhibits enhanced tumor penetration, prolonged retention, and sustained glucose depletion in the tumor microenvironment. When combined with a fatty acid oxidation inhibitor, it implements dual metabolic suppression, thereby enhancing ROS‐induced immunogenic cell death and reprogramming tumor immune microenvironment. Further combination with the immune checkpoint inhibitor αPD‐1 significantly suppresses distant tumor growth and achieves complete survival in 100% of tumor‐bearing mice.

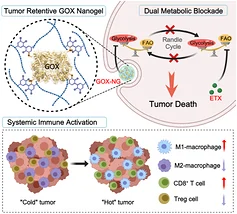

## 1 Introduction

Due to their rapid proliferation and aberrant growth characteristics, tumors exhibit profound metabolic reprogramming, including aerobic glycolysis (the Warburg effect), glutamine addiction, and reliance on alternative energy sources [1]. This metabolic plasticity is critical for tumor initiation, progression, metastasis, and even modulates responses to immunotherapy, making targeting tumor metabolism a promising therapeutic avenue [2, 3, 4, 5, 6, 7, 8, 9]. However, recent studies indicate that conventional starvation‐based therapies focusing on single metabolic pathways often yield limited efficacy and, in some cases, may even paradoxically promote metastasis [10, 11]. Therefore, developing combined therapies that incorporate spatiotemporally coordinated, multi‐pathway metabolic inhibition with existing immune checkpoint blockade represents a valuable direction for future anticancer research.

The Randle cycle, first described in the 1960s, refers to the reciprocal inhibition and compensation between glucose and lipid metabolism within cells [12]. Although this concept has been widely applied in the context of diabetes and nutrition research, its relevance in tumor metabolism has been largely overlooked [13]. In cancer cells, glucose and lipid metabolism exhibit a similar compensatory relationship. Inhibiting one pathway often leads to the upregulation of the other, undermining therapeutic efficacy. For instance, inhibition of lipid oxidation using etomoxir (ETX) can induce compensatory enhancement of glycolysis, reducing the impact of fatty acid oxidation (FAO) suppression. Conversely, inhibition of glycolysis may prompt increased FAO activity [14, 15, 16]. Therefore, dual inhibition of both glycolytic and lipid metabolic pathways holds promise for enhancing antitumor efficacy. However, most known inhibitors are small molecules with short half‐lives, rendering them unsuitable for sustained metabolic suppression. This mismatch in pharmacokinetics compromises the possibility of achieving spatial and temporal synergy, ultimately limiting treatment outcomes. Inspired by the physiological principles of the Randle cycle, we hypothesized that temporally coordinated dual blockade of glucose and fatty acid metabolism enabled by a tumor‐retentive nanocarrier could overcome compensatory metabolic rewiring and potentiate therapeutic efficacy.

Glucose oxidase (GOX), due to its unique ability to consume glucose while generating hydrogen peroxide (H_2_O_2_), has attracted attention as a means of inducing both starvation and oxidative stress in tumors [17, 18, 19, 20]. However, as an exogenous enzyme derived from Aspergillus niger, GOX suffers from several limitations that hinder its clinical translation—namely, high immunogenicity, poor stability, limited pharmacokinetics, inadequate tissue penetration, and systemic toxicity [21]. These drawbacks lead to rapid clearance and poor accumulation in tumor tissues, preventing sustained glucose depletion and failing to create the low‐glucose microenvironment critical for dual metabolic inhibition strategies.

To overcome these barriers, we herein propose a nanogel‐based delivery system for GOX. Nanogels are nanoscale, hydrophilic polymer networks formed through chemical or physical crosslinking, combining the structural features of nanoparticles with the water‐rich matrix of hydrogels [22, 23, 24]. With sizes typically ranging from tens to several hundred nanometers, nanogels exhibit excellent biocompatibility and are considered ideal carriers for protein delivery [25, 26]. In this work, we constructed a GOX‐loaded nanogel (GOX‐NG) by using catechol‐functionalized alginate (Cat‐Alg) as the scaffold, wherein GOX acts as the crosslinking node. This GOX‐NG system demonstrated superior tumor tissue penetration and retention, enabling effective suppression of tumor glycolysis and significantly enhancing the sensitivity of tumors to FAO inhibition. This dual metabolic blockade remodels the tumor's local metabolic environment, promotes oxidative stress, and activates antitumor immune responses. When combined with immune checkpoint blockade therapy, the strategy not only resulted in substantial inhibition of primary tumors but also showed efficacy against distant metastatic lesions—highlighting its potential in curbing tumor metastasis. Altogether, this GOX‐NG platform provides a robust foundation for implementing dual inhibition of glucose and lipid metabolism and opens new avenues for integrated starvation–oxidation–immunotherapy in cancers such as oral squamous cell carcinoma (OSCC).

## 2 Results

### 2.1 Synthesis and Characterization of GOX Nanogels

**FIGURE 1:**
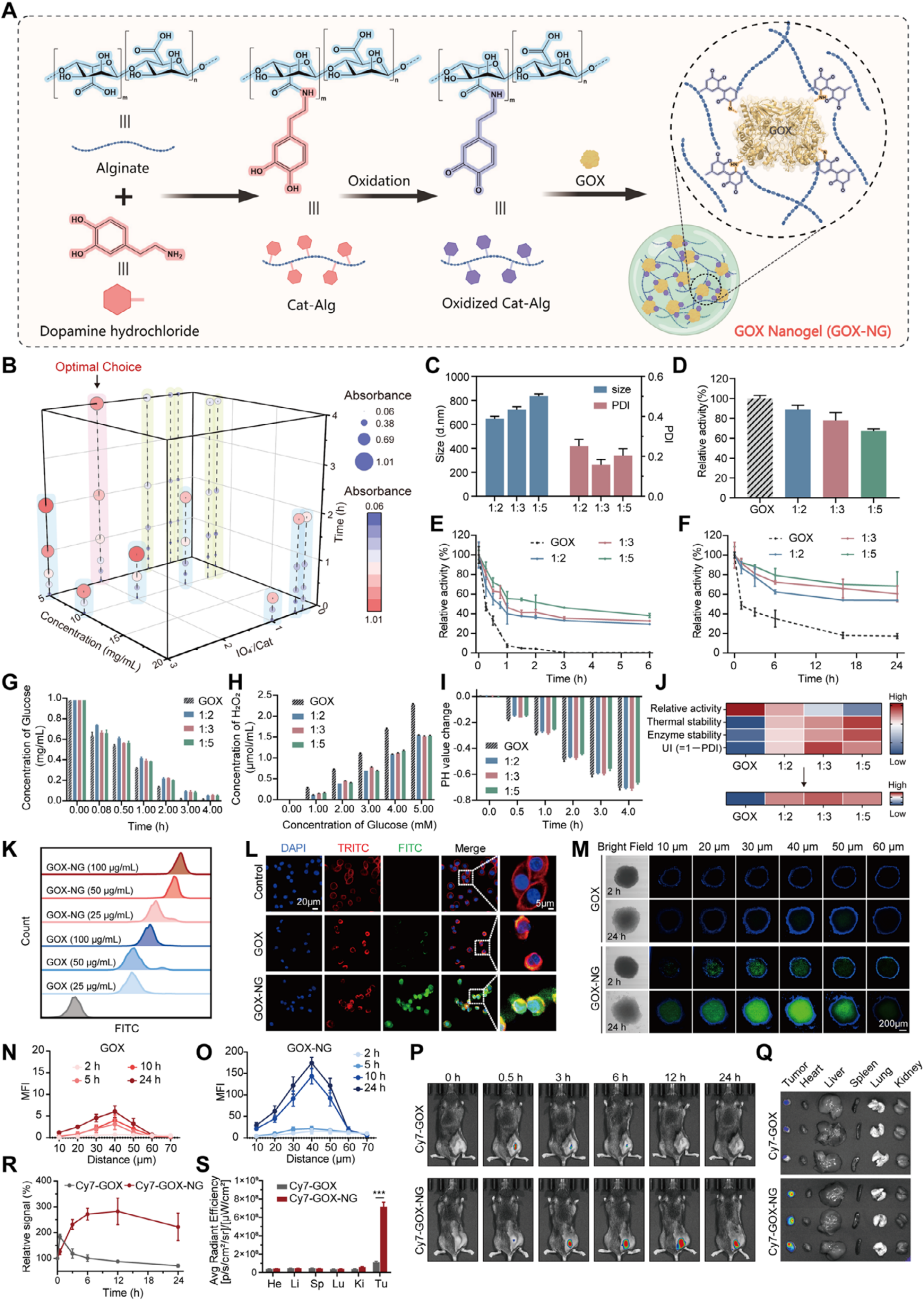
Characterization of physicochemical properties and tumor retention ability of GOX‐NG. (A) Schematic illustration of the GOX‐NG preparation process. (B) Screening of optimal oxidation conditions for Cat‐Alg (Different levels of absorbance were represented by the size and color of the symbol). The pink‐highlighted conditions represented the optimum. Yellow‐highlighted ones showed excessively low peak absorption, indicating insufficient reactive groups. Blue‐highlighted conditions had overly short oxidation times, leading to rapid oxidation and macroscopic gel formation, which was undesirable for nanogel preparation. (C) Particle size and PDI of 3 different ratios of GOX‐NGs (*n* = 3). (D) Relative enzyme activities of 3 different ratios of GOX‐NGs (*n* = 3). (E) Thermal stability of GOX and GOX‐NGs at 60°C (*n* = 3). (F) Changes in the relative enzyme activities of GOX and GOX‐NGs incubated with proteinase K at 37°C (n = 3). (G) Glucose content at different time points after incubation of GOX or GOX‐NGs with glucose (5 mM) (*n* = 3). (H) Hydrogen peroxide content in solution after 4 h of incubation of GOX or GOX‐NGs with different concentrations of glucose (*n* = 3). (I) pH Changes in solution at different time points after incubation of GOX or GOX‐NGs with glucose (5 mM) (*n* = 3). (J) Optimal screening of GOX‐NG. (K) After co‐incubation of different concentrations of FITC‐labeled GOX or GOX‐NG with SCC7 cells for 2 h, cellular uptake was measured by flow cytometry. (L) The fluorescence distribution of FITC‐labeled GOX or GOX‐NG in SCC7 cells after cellular uptake. (M) After co‐incubation of FITC‐labeled GOX‐NG with SCC7 tumor spheres, the permeability of GOX‐NG in the tumor spheres was detected at different time points. Fluorescence images of tumor spheres at different depths taken by CLSM. (N‐O) Fluorescence quantification of GOX and GOX‐NG at different depths of the tumor spheres (*n* = 3). (P‐Q) SCC7‐bearing C3H mice were treated with Cy7‐labeled GOX or GOX‐NG by intratumor injection. In vivo fluorescence imaging of the mice was taken at different time points, and ex vivo fluorescence imaging of the mice tumors and organs 24 h after injection. (R) Fluorescence intensity of tumors in mice over time (*n* = 3). (S) Quantification of fluorescence intensity *in* ex vivo tumors and organs of mice 24 h after injection (*n* = 3). All data are presented as mean ± SEM. ***p < 0.001.

To fabricate the GOX‐loaded nanogel, we first synthesized catechol‐functionalized alginate (Cat‐Alg) through an NHS/EDC‐mediated amidation reaction between dopamine hydrochloride and alginate (Figure 1A). Alginate, a naturally derived polysaccharide with excellent biocompatibility, is widely used in biomedical hydrogel systems [27]. The introduction of catechol groups not only provided reactive sites for crosslinking but also imparted adhesive properties to the polymer [28]. Successful modification of catechol onto the alginate backbone was confirmed by ^1^H NMR spectroscopy and UV‐vis absorption spectra (Figures S1 and S2), with a grafting rate of 177.2 µmol/1 g Cat‐Alg. Subsequently, sodium periodate was used to oxidize the catechol moieties into quinone structures, which could further react with amine or thiol groups on the GOX protein via Schiff base formation or Michael addition (Figure 1A). However, excessive oxidation may lead to intermolecular crosslinking between quinone groups themselves, reducing available conjugation sites for GOX and potentially forming bulk black gels unsuitable for injectable applications [29]. Therefore, we optimized the oxidation conditions by monitoring the increase in absorbance at 460–475 nm via UV‐vis spectroscopy, which reflects quinone formation (Figures S3–S5). Based on the balance between oxidation efficiency and solution injectability, the optimal condition was determined to be Cat‐Alg at 5 mg mL^−1^, oxidized for 4 h with a Cat/IO_4_^−^ molar ratio of 1:2 (Figure 1B). Upon mixing oxidized Cat‐Alg with GOX, GOX nanogels (GOX‐NG) formed spontaneously via covalent crosslinking. To tune gel properties, we prepared three formulations with different GOX‐to‐Cat‐Alg ratios (1:2, 1:3, and 1:5). SDS‐PAGE analysis showed that the nanogels largely remained in the sample wells with minimal migration into the gel, indicating successful formation of high‐molecular‐weight nanostructures (Figure S6). Dynamic light scattering (DLS) measurements revealed hydrated diameters of 646 ± 22, 723 ± 25, and 836 ± 19 nm for the respective formulations, with zeta potentials of −27.5 ± 0.6, −31.5 ± 2.1, and −32.5 ± 1.7 mV and PDI values of 0.25 ± 0.03, 0.16 ± 0.03, and 0.20 ± 0.03, respectively (Figure 1C and Figures S7, S8). In contrast, transmission electron microscopy (TEM) images showed significantly smaller particle sizes, likely due to dehydration and shrinkage under vacuum during sample preparation (Figure S9).

To identify the most suitable formulation for antitumor applications, we evaluated the enzyme activity and in vitro stability of the three GOX‐NG systems. Enzymatic assays indicated that higher alginate content correlated with reduced GOX activity, possibly due to steric shielding of catalytic sites by polymer chains (Figure 1D). Further stability assessments revealed that GOX‐NG exhibited significantly improved thermal and proteolytic stability compared to free GOX (Figure 1E,F). Despite this, the nanogels retained sufficient catalytic function, consuming over 90% of glucose within 4 h, generating H_2_O_2_ concentrations exceeding 1 mM, and decreasing the solution pH by 0.7 units (Figure 1G–I). Increasing alginate content further enhanced this resistance, likely due to the protective network of the nanogel scaffold. These enhancements in stability are critical for in vivo application. Based on the balance of size uniformity, enzymatic activity, and environmental stability, we selected the 1:3 GOX‐to‐Cat‐Alg formulation for subsequent studies (Figure 1J).

We also evaluated the stability of GOX under various physiological conditions. We measured the particle size stability of GOX‐NG in H_2_O, DMEM, and PBS at 37°C daily over a one‐week period. The results showed that the particle size of GOX‐NG remained virtually unchanged over the course of the week, indicating good particle size stability under physiological conditions (Figure S10). In addition, we monitored changes in GOX‐NG enzyme activity during incubation at 37°C. The results showed that GOX‐NG maintained stable and high enzyme activity over the course of a week (Figure S11). This approach offers a simple, biocompatible method to encapsulate and stabilize GOX, representing a distinct improvement over previously reported systems.

### 2.2 Enhanced Tumor Penetration and Retention of GOX Nanogels

We next investigated the tumor penetration and retention properties of GOX‐NG both in vitro and in vivo. Initially, we assessed cellular uptake by co‐culturing GOX‐NG with SCC7 oral squamous carcinoma cells. Flow cytometry and confocal laser scanning microscopy (CLSM) revealed a striking enhancement in intracellular uptake of GOX when delivered via the nanogel system, exhibiting a clear dose‐dependent trend (Figure 1K,L and Figure S12). To our knowledge, although alginate is commonly known as a negatively charged, naturally derived polymer, its ability to enhance intracellular delivery of exogenous proteins through nanogel encapsulation has not been previously reported.

To evaluate tumor penetration in a 3D context, we utilized a multicellular tumor sphere (MCTS) model. Confocal microscopy was used to image fluorescence distribution within spheroids at multiple time points (Figure 1M–O and Figure S13). The results were compelling: compared to free GOX, GOX‐NG exhibited significantly greater penetration depth, with a 29.2‐fold increase in total fluorescence intensity (quantified by area under the curve analysis), demonstrating its superior tumor infiltration capacity.

We then assessed in vivo retention by intratumorally injecting equivalent doses of GOX and GOX‐NG into SCC7 tumor‐bearing mice (Figure 1P–S). Fluorescence imaging showed that GOX‐NG exhibited substantially higher retention within the tumor over time. At 24 h post‐injection, the intratumoral concentration of GOX‐NG was 6.5 times higher than that of free GOX. This enhanced retention can be attributed to several factors, including the nanogel's larger size, improved structural stability, stronger tissue adhesion conferred by catechol groups, and increased cellular uptake efficiency. In addition, we measured serum GOX concentrations at various time points following intratumoral injection of GOX or GOX‐NG. The results showed that serum GOX levels in the free GOX group rose rapidly 1 min after injection, peaked at 0.5 h, and were significantly higher than those in the GOX‐NG group, indicating that GOX rapidly leaked from the tumor region into the circulatory system and was rapidly cleared within 6 h. In contrast, serum GOX concentrations in the GOX‐NG group rose slightly at 0.5 h and subsequently remained at extremely low levels, suggesting that a large amount of GOX‐NG was confined to the tumor region without significant leakage (Figure S14). The superior penetration and retention properties of GOX‐NG are highly advantageous for tumor‐targeted delivery. Not only do they reduce off‐target toxicity to healthy tissues, but they also sustain glucose depletion within the tumor microenvironment. This prolonged metabolic suppression is essential for successful dual inhibition of glucose and lipid metabolism, laying the foundation for subsequent combination with fatty acid oxidation inhibitors.

### 2.3 In Vitro Antitumor Efficacy and Synergy of GOX‐NG and Etomoxir

**FIGURE 2:**
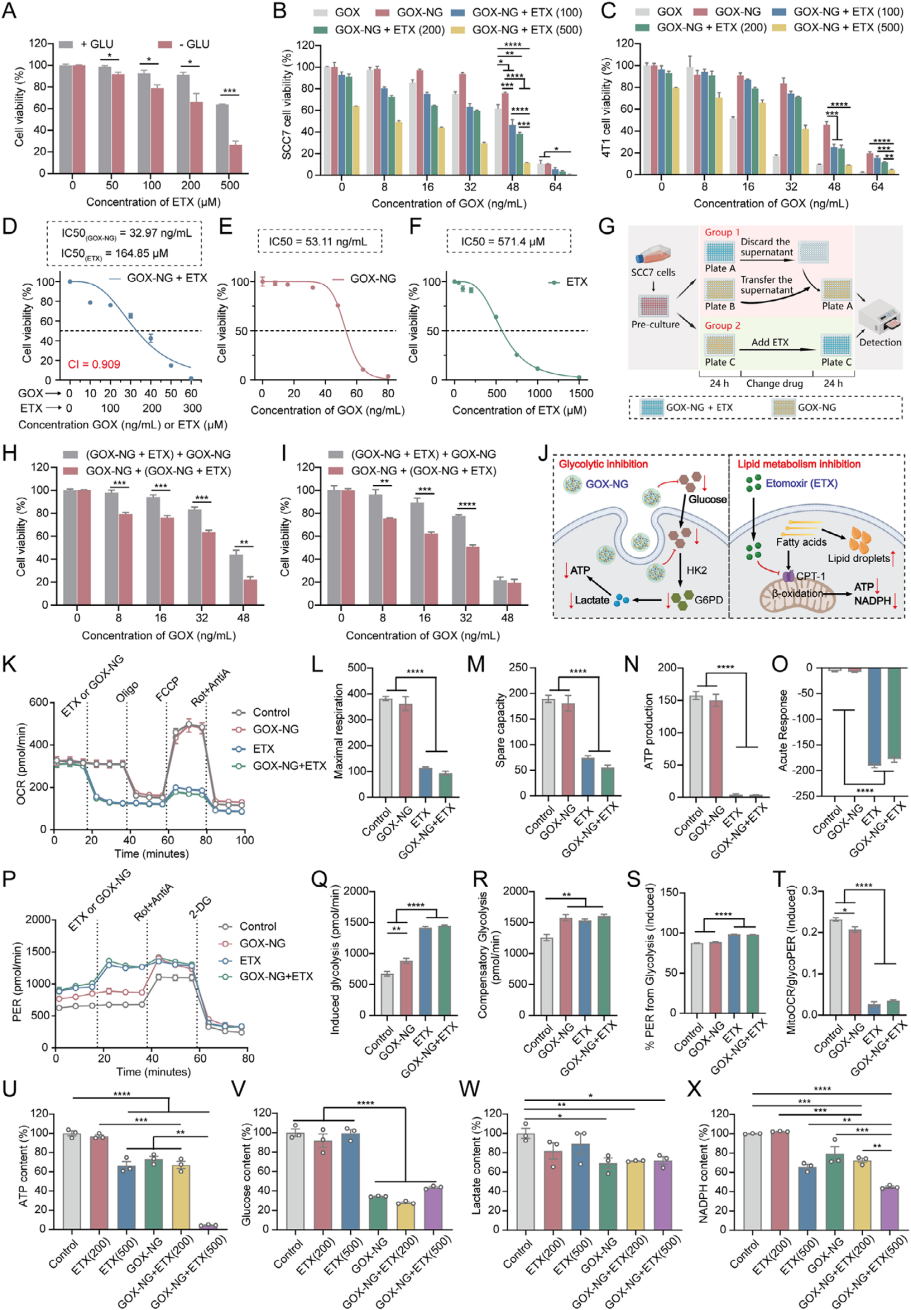
In vitro cell experiments to verify the antitumor toxic effect of GOX‐NG combined with ETX and the mechanism of enhanced starvation therapy. (A) The toxicity of ETX on SCC7 cells was examined in medium with or without glucose (n = 6). (B) The toxic effect of GOX‐NG + ETX on SCC7 cells determined by CCK8 assay (*n* = 3). (C) The toxic effect of GOX‐NG + ETX on 4T1 cells was determined by CCK8 assay (*n* = 3). (D) Combination Index (CI) and IC50 of GOX‐NG + ETX (*n* = 3). (E) IC50 of GOX‐NG (*n* = 3). (F) IC50 of ETX (*n* = 3). (G–I) Schematic illustration and CCK8 results of the toxic effects on SCC7 cells when ETX was added at different times in the presence of GOX‐NG (ETX = 50 µM or 100 µM) (*n* = 4). (J) Schematic illustration of GOX‐NG in combination with ETX to enhance tumor starvation therapy. (K) Cell mitochondrial stress test assay profile (*n* = 3). (L–O) Maximal respiration, reserve respiration, ATP production, and acute response were quantified by mitochondrial stress assay (*n* = 3). (P) Glycolytic rate assay profile (*n* = 3). (Q–T) Induced glycolysis, compensatory glycolysis, %PER from glycolysis (induced), and mitoOCR/glycoPER (induced) quantified by glycolytic rate assay (*n* = 3). (U–X) Intracellular ATP content, glucose content in the supernatant, lactate content in the supernatant, and NADPH content of SCC7 cells after 24 h of co‐incubation with different drug regimens (*n* = 3). All data are presented as mean ± SEM. *p < 0.05, **p < 0.01, ***p < 0.001 and ****p < 0.0001.

To evaluate the antitumor effects and underlying mechanisms of GOX‐NG in combination with the fatty acid oxidation inhibitor ETX, we conducted a series of in vitro studies. ETX is a well‐characterized inhibitor that selectively targets carnitine palmitoyl transferase 1 (CPT1) on the mitochondrial membrane, effectively blocking the entry of fatty acids into mitochondria for β‐oxidation [5]. Its antitumor potential has been explored in glioblastoma, non‐small cell lung cancer, and bladder cancer, and it has already entered clinical trials [5]. We first examined the toxic effect of ETX on SCC7 oral squamous carcinoma cells in glucose or glucose‐free medium. The results showed that the proliferation of SCC7 in the glucose‐containing medium was hardly affected (cell viability >90%) when treated with ETX at concentrations up to 200 µM. However, cell proliferation was significantly inhibited in the glucose‐free medium, indicating that glucose deprivation can significantly enhance cells' sensitivity to ETX. (Figure 2A). Next, we examined whether GOX‐NG could also achieve the same ETX sensitization effect by consuming glucose. The CCK8 results of SCC7 and 4T1 showed that, when treated with GOX‐NG, even a relatively low concentration of ETX could significantly inhibit cell proliferation. This verified that the glucose deprivation produced by GOX‐NG made cells more sensitive to fatty acid metabolism. Similarly, in the presence of ETX, low concentrations of GOX‐NG had a significant inhibitory effect on cell proliferation, indicating that inhibiting fatty acid metabolism would also make tumor cells more sensitive to glucose deprivation (Figure 2B,C and Figure S15). Combination index (CI) analysis yielded a value of 0.909, confirming a synergistic interaction between GOX‐NG and ETX (Figure 2D–F). Live‐cell confocal microscopy and flow cytometry further demonstrated enhanced apoptosis in the combination group, reinforcing the therapeutic advantage of dual metabolic inhibition (Figures S16 and S17).

To explore the dynamics of metabolic reprogramming in response to nutrient stress, we designed a timing‐controlled experiment. Under otherwise identical conditions—including exposure duration, culture time, and medium composition—we compared the effects of adding ETX either simultaneously with GOX‐NG or 24 h later. Interestingly, delaying ETX administration by 24 h led to significantly stronger inhibition of tumor cell proliferation (Figure 2G–I). This finding suggests that tumor cells require time to adapt to glucose scarcity by upregulating fatty acid oxidation as a compensatory pathway. Targeting FAO after this metabolic shift enhances treatment efficacy, providing a rationale for temporally coordinated therapeutic intervention.

Furthermore, we measured the kinetics of changes in intratumoral glucose levels following intratumoral injection of GOX‐NG (Figure S18). The results showed that intratumoral glucose levels dropped rapidly following GOX‐NG administration, falling below 20% of baseline levels within 6–24 h, and remained significantly suppressed throughout this time window. However, glucose levels began to rebound 48 h post‐injection, indicating the presence of a transient glucose‐deprived metabolic window. Therefore, in subsequent in vivo experiments, we established a 24‐hour interval between the administration of GOX‐NG and ETX, with the aim of aligning ETX delivery with the peak window of glucose deprivation, thereby maximizing metabolic vulnerability.

### 2.4 Real‐Time Metabolic Analysis Confirms Dual Pathway Suppression

Theoretically, GOX‐NG can block energy supply both inside and outside cells by directly consuming glucose, reduce ATP production, and simultaneously decreasing the accumulation of the metabolic product lactic acid. ETX blocks the entry of fatty acids into mitochondria for energy supply by inhibiting the CPT‐1 transporter, resulting in the accumulation of lipid droplets in the cytoplasm and the reduction of ATP and NADPH (Figure 2J). To verify the potential mechanism of GOX‐NG and ETX combination, we conducted real‐time metabolic analysis and metabolite content tests. Firstly, we performed mitochondrial and glycolysis stress tests using the Seahorse XF96 analyzer. For the mitochondrial stress test, GOX‐NG or ETX was sequentially added to the cells, followed by oligomycin, FCCP, and a combination of rotenone and antimycin A. The resulting oxygen consumption rate (OCR) profiles revealed that both ETX and the combination group caused a marked decrease in mitochondrial respiration compared to GOX‐NG alone or untreated controls. Quantitative analyses of maximal respiration, reserve capacity, ATP production, and acute response confirmed that the combination of GOX‐NG and ETX most effectively suppressed mitochondrial energy output (Figure 2K–O).

In glycolytic stress tests, treatment with ETX induced a compensatory increase in glycolysis, as evidenced by elevated proton efflux rate (PER), induced and compensatory glycolysis, and the percentage of glycolysis‐derived PER. These results indicate that tumor cells possess considerable metabolic flexibility, shifting toward glycolysis when FAO is blocked (Figure 2P–T). Furthermore, when GOX‐NG and ETX were used in combination, both glycolytic and oxidative energy pathways were significantly inhibited, leading to a sharp drop in intracellular ATP levels (Figure 2U and Figure S19). Further biochemical analyses revealed significant reductions in extracellular glucose and lactate concentrations, as well as intracellular NADPH (Figure 2V–X and Figures S20, S21). Due to the inhibition of CPT1, fatty acids could not enter the mitochondria and accumulated in the cytoplasm, forming more lipid droplets. (Figure S22). Together, these findings confirm that the dual inhibition strategy effectively suppresses energy metabolism in tumor cells through complementary blockade of glycolysis and fatty acid oxidation, exhibiting its great potential in tumor starvation therapy.

### 2.5 Combination Therapy Induces Severe Oxidative Stress

**FIGURE 3:**
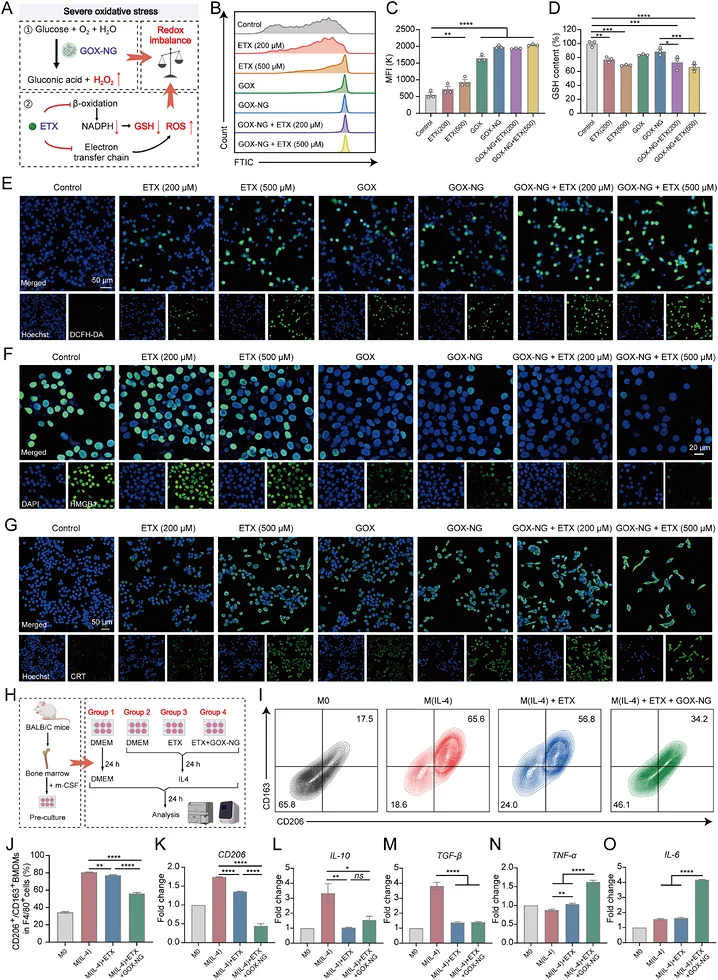
In vitro cell experiments to verify that GOX‐NG combined with ETX enhanced oxidative therapy by enhancing oxidative stress and enhanced immunotherapy by inducing ICD and blocking M2 polarization of macrophages. (A) Schematic illustration of the mechanism that enhanced oxidative stress. (B,C) SCC7 cells were incubated with different regimens for 24 h, and ROS levels and quantification were measured by flow cytometry (*n* = 3). (D) Intracellular GSH content of SCC7 cells after incubation with different regimens for 24 h (*n* = 3). (E) Representative CLSM images of ROS in SCC7 cells after incubation with different regimens for 24 h. (F,G) Representative CLSM images of HMGB1 and CRT in SCC7 cells after incubation with different regimens for 24 h. (H) Schematic illustration of an experiment to verify the blocking of M2 polarization by ETX. (I,J) Flow cytometric analysis for the CD206^+^/CD163^+^ population in BMDM after co‐incubation with different drug regimens (*n* = 3). (K–O) qPCR analysis of CD206, IL10, TGF‐β, TNF‐α, and IL6 in BMDM after co‐incubation with different drug regimens (*n* = 3). All data are presented as mean ± SEM. *p < 0.05, **p < 0.01, ***p < 0.001 and ****p < 0.0001.

In addition to nutrient deprivation, GOX catalyzes the oxidation of glucose to gluconic acid and hydrogen peroxide (H_2_O_2_), a potent reactive oxygen species (ROS). The FAO pathway is the main source of intracellular NAPDH, generating reduced glutathione (GSH) to capture excessive ROS within cells, thereby promoting cell survival [30]. Therefore, ETX‐induced FAO inhibition leads to a decrease in intracellular GSH and an increase in ROS (Figure 3A and Figure S23). Elevated ROS levels are known to induce oxidative stress and trigger tumor cell death. To examine the oxidative stress induced by GOX‐NG and ETX, we measured intracellular ROS levels in SCC7 cells using a ROS assay kit and flow cytometry (Figure 3B,C). GOX‐NG treatment significantly increased ROS accumulation compared to controls. Notably, high concentration of ETX alone significantly elevated ROS levels—an effect observed in prior studies, potentially linked to activation of NAD(P)H oxidases, mitochondrial damage, or impaired ATP production leading to reduced synthesis of antioxidant GSH [31, 32]. This result might partially explain the cytotoxicity caused by ETX at high concentrations in the previous CCK8 test. Meanwhile, GSH quantification showed a marked reduction in antioxidant capacity following GOX‐NG or ETX treatment, with the most significant depletion in the combined group (Figure 3D). Confocal imaging confirmed robust intracellular ROS accumulation in the combination group (Figure 3E). It's been widely reported that ROS can lead to immunogenic cell death (ICD), which produces “eat me” signal, attracting and activating antigen‐presenting cells, ultimately triggering an adaptive immune response [33, 34, 35]. Therefore, we assessed ROS‐induced ICD effects by measuring its key marker HMGB1 and calreticulin (CRT). Results showed that consistent with ROS levels, high concentrations of ETX, GOX‐NG, and the combination therapy group all induced significant HMGB1 release, CRT exposure, extracellular ATP release, and surface expression of HSP70, with the combination therapy group exhibiting the most pronounced effect (Figure 3F,G and Figures S24–S27). Together, these data indicate that dual treatment induces severe oxidative stress and ICD, further contributing to tumor cell killing and supporting the oxidation therapy mechanism of the GOX‐NG+ETX combination.

### 2.6 Inhibition of Immunosuppressive Macrophage Polarization

Tumor‐associated macrophages (TAMs) often adopt an M2‐like, immunosuppressive phenotype within the tumor microenvironment. However, when appropriately stimulated, they can be reprogrammed to the M1 phenotype, which exhibits pro‐inflammatory and antitumor functions [36]. Previous reports suggest that ETX can inhibit M2 polarization [37, 38, 39, 40, 41]. To validate this, we harvested bone marrow‐derived macrophages (BMDMs) from mice, induced M2 polarization using IL‐4, and treated cells with ETX or the GOX‐NG+ETX combination (Figure 3H). Flow cytometry analysis revealed that ETX alone reduced the proportion of M2 macrophages (CD206^+^/CD163^+^) from 80.63% to 77.17%, while the combination group showed a more substantial reduction to 56.17% (Figure 3I,J). This enhanced effect may stem from the high levels of ROS generated by GOX‐NG, which may independently impair M2 polarization [42, 43]. RT‐qPCR analysis further confirmed these observations: expression of M2‐associated anti‐inflammatory genes (CD206, IL‐10, TGF‐β) was significantly downregulated, while expression of M1‐associated pro‐inflammatory genes (TNF‐α, IL‐6) was upregulated (Figure 3K–O). These results support the idea that the GOX‐NG and ETX combination effectively reprograms macrophages and modulates the tumor immune microenvironment. This effect, together with the induction of ICD, providing a rationale for its further combination with immunotherapy.

### 2.7 Transcriptomic Profiling Reveals Multidimensional Mechanisms of Action

**FIGURE 4:**
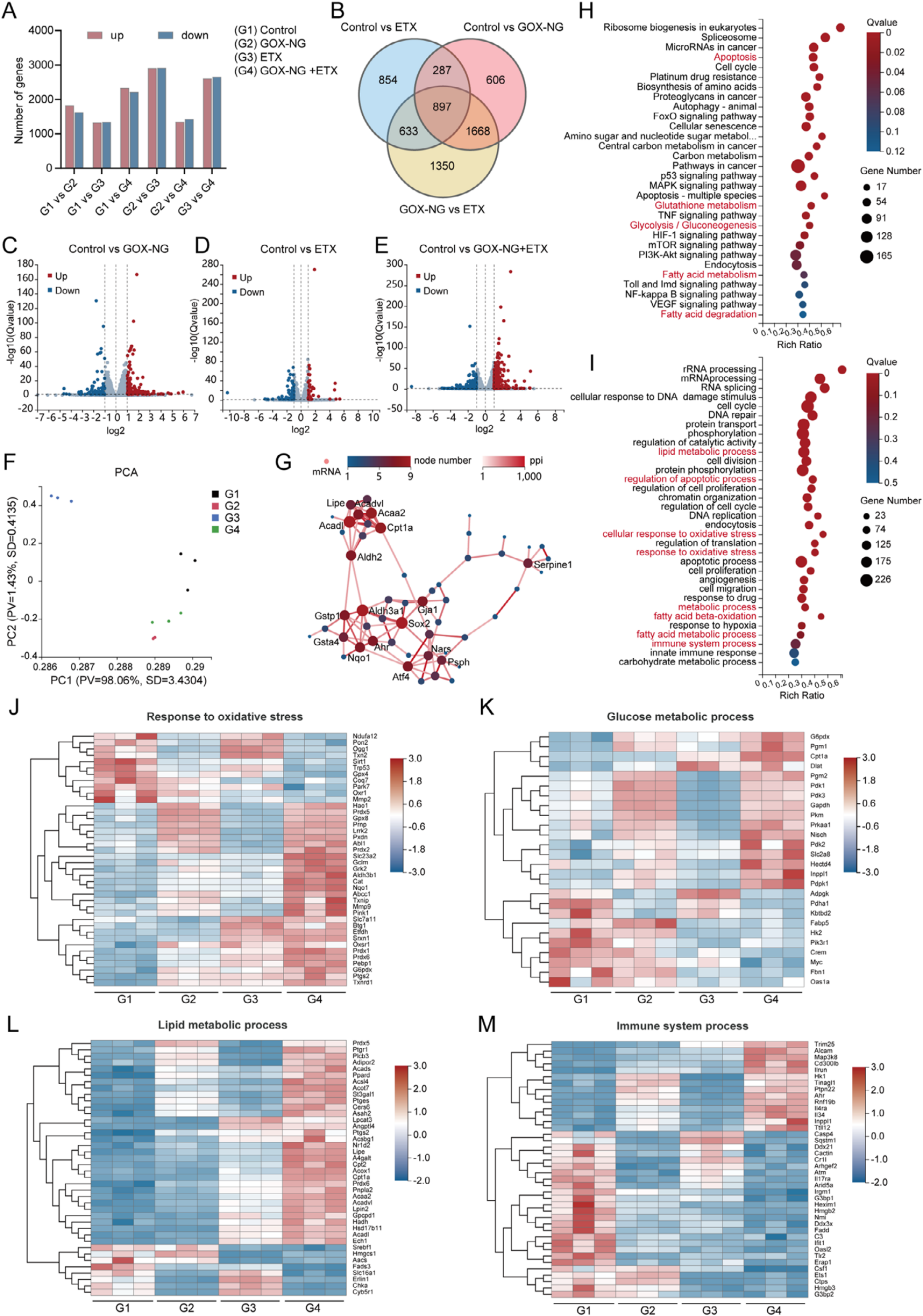
RNA‐seq analysis. SCC7 cells were incubated with different drug regimens for 24 h, and RNA was extracted for transcriptome sequencing analysis. (A) The number of up‐regulated and down‐regulated differentially expressed genes (DEGs) between different groups. (B) Venn diagram of DEGs between different groups. (C–E) Volcanic maps of DEGs between Control vs GOX‐NG, Control vs ETX, and Control vs GOX‐NG+ETX. (F) Principal component analysis (PCA) of the correlation of gene expression profiles between different groups. (G) The interaction network consists of proteins encoded by representative DEGs. (H) Kyoto Encyclopedia of Genes and Genomes (KEGG) classification of DEGs between Control vs GOX‐NG+ETX. (I) GO classification of DEGs between Control versus GOX‐NG+ETX. (J–M) Expression profiles of representative DEGs in different groups related to response to oxidative stress, glucose metabolism process, lipid metabolism process, and immune system process.

To further elucidate the molecular mechanisms underpinning the starvation–oxidation–immunotherapy strategy, we performed RNA sequencing (RNA‐seq) on SCC7 cells treated with PBS (control), ETX, GOX‐NG, or the GOX‐NG+ETX combination (*n* = 3 per group). Differential gene expression analysis revealed that compared to the control, the GOX‐NG+ETX group exhibited extensive transcriptomic changes, with 2330 genes upregulated and 2218 genes downregulated (Figure 4A and Figures S28, S29). Venn diagrams indicated substantial overlap in differentially expressed genes (DEGs) among groups, while volcano plots highlighted the scale and intensity of transcriptional modulation (Figure 4B–E and Figure S30). Principal component analysis (PCA) and Pearson correlation coefficient heatmap demonstrated clear segregation between treatment groups and high consistency among replicates (Figure 4F and Figure S31). Protein‐protein interaction network analysis revealed the critical roles of key proteins in lipid metabolism pathways, such as Cpt1a, Acaa2, and Acadl, as well as proteins associated with cellular oxidative stress pathways, including Atf4, Aldh3a1, Nqo1, and Gstp1, reflecting the dual metabolic and redox stress imposed by the treatment (Figure 4G). To gain deeper insights into functional pathways, we performed Kyoto Encyclopedia of Genes and Genomes (KEGG) and Gene Ontology (GO) enrichment analyses. KEGG results showed that in the GOX‐NG+ETX group, pathways related to nutrient metabolism, oxidative stress, and cell death were the most affected. GO enrichment similarly revealed alterations in processes involving the cell cycle, metabolism, oxidative response, and immune activity (Figure 4H,I and Figures S32–S35). Furthermore, we clustered gene expression patterns related to four major biological processes: oxidative stress response, glucose metabolism, lipid metabolism, and immune system function. Heatmaps clearly demonstrated that the combination group had the most profound impact across all four axes, suggesting a comprehensive reshaping of tumor cell homeostasis through synergistic metabolic, redox, and immune modulation (Figure 4J–M). Based on the above evidence, this study provides a foundation for understanding the potential mechanism of the GOX‐NG+ETX combination in tumor starvation‐oxidation‐immunotherapy.

### 2.8 In Vivo Antitumor Efficacy and Synergy With Immune Checkpoint Blockade

**FIGURE 5:**
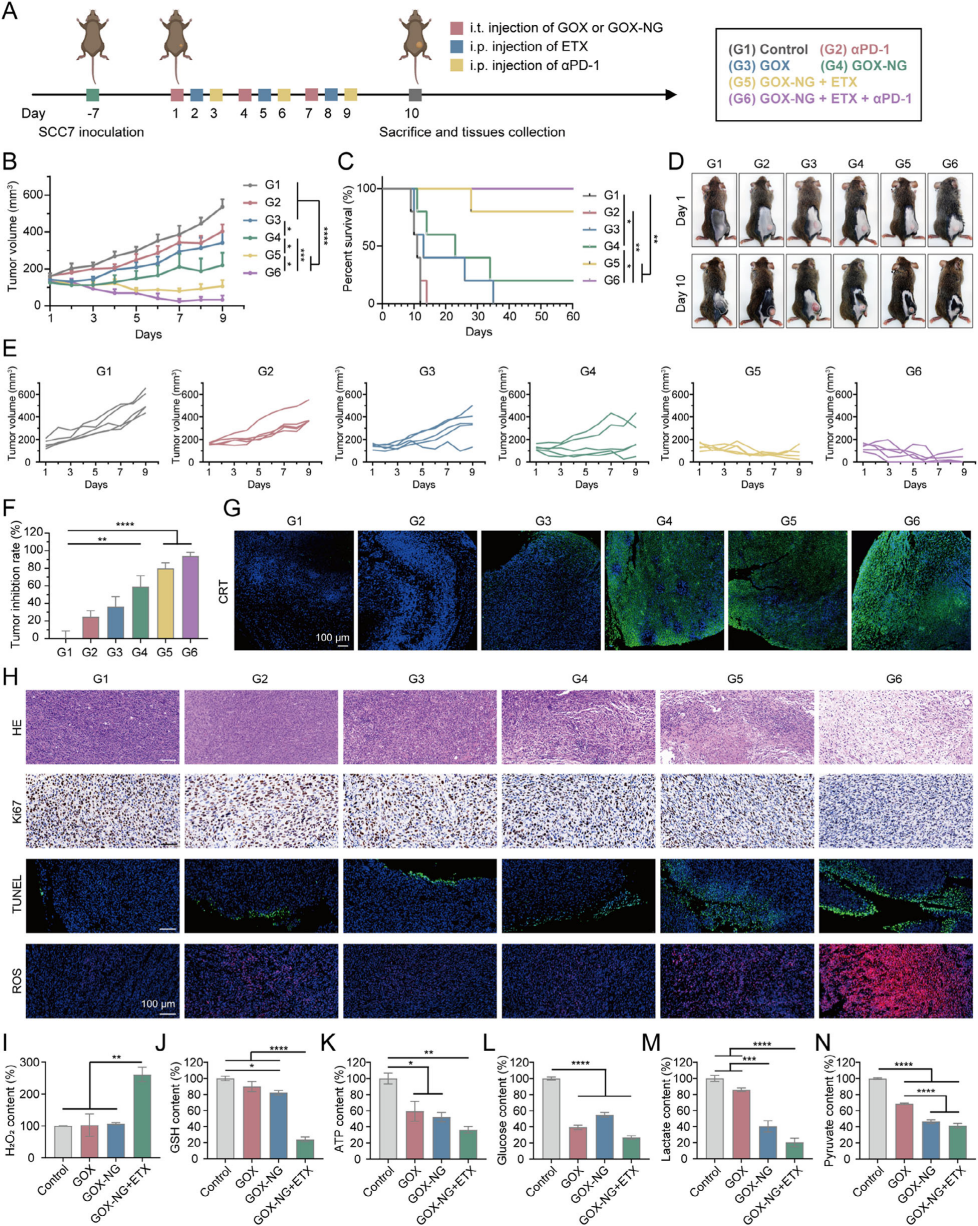
In vivo antitumor efficacy of combination therapy with GOX‐NG + ETX and αPD‐1. (A) Schematic illustration of the therapeutic schedule. (B) Daily average tumor volume in different treatment groups (*n* = 5). (C) Survival of mice in different treatment groups (*n* = 5). (D) Bright‐field photographs of representative mice of different treatment groups before and after treatment. (E) Tumor volume growth curves for each mouse in different treatment groups. (F) Tumor inhibition rates in different treatment groups (*n* = 5). (G,H) Representative images of CRT, HE staining, Ki67 staining, TUNEL staining and ROS staining of tumor tissue sections. (I–N) H_2_O_2_ content, GSH content, ATP content, glucose content, lactate content and pyruvate content in tumors after treatment (n = 5). All data are presented as mean ± SEM. *p < 0.05, **p < 0.01, ***p < 0.001 and ****p < 0.0001.

Through the above in vitro experiments, we have validated the antitumor effects of GOX‐NG+ETX and its mechanisms in starvation therapy, oxidative stress, and immune regulation. Moreover, we found that GOX‐NG+ETX treatment could induce ICD and block the polarization of tumor‐promoting M2 macrophages, as well as alleviate the accumulation of lactic acid that is recently been found to be a vital molecule promoting immune escape and tumor progression [44, 45]. Lactate is known to inhibit T cell function by suppressing glycolysis and promoting T cell exhaustion through GPR81‐mediated PD‐1 upregulation, while also driving M2‐like macrophage polarization via HIF‐1α–VEGF and lactylation‐dependent pathways, collectively contributing to an immunosuppressive microenvironment that can be reversed by lactate reduction [46]. Given the potential contribution of GOX‐NG+ETX to tumor inhibition and immune remodeling, we next evaluated the therapeutic efficacy of GOX‐NG+ETX in vivo and further assessed its potential to synergize with immune checkpoint blockade (ICB) therapy. A subcutaneous SCC7 tumor model was established in C3H mice. Before efficacy testing, a dose‐escalation study determined that GOX‐NG was significantly better tolerated than free GOX, with a maximum tolerated dose of 8 mg kg^−1^ (GOX‐NG) versus 2 mg kg^−1^ (GOX), respectively—likely due to enhanced tumor retention and reduced systemic exposure enabled by the nanogel delivery system (Figure S36). When tumor volumes reached approximately 120 mm^3^, mice were treated with GOX‐NG and ETX, with a 24‐hour interval between administrations to optimize metabolic synchronization (Figure 5A). Compared to either monotherapy, the combination group of GOX‐NG and ETX exhibited significantly improved tumor suppression and prolonged survival, achieving an 80% survival rate at day 60. Strikingly, the addition of an αPD‐1 further enhanced therapeutic efficacy. This triple combination therapy led to complete tumor regression in 60% of mice and a 100% survival rate. Tumor inhibition reached 93.8% (Figure 5B–F and Figure S37). Histological results revealed that obvious CRT exposure was observed in the GOX‐NG‐containing treatment groups, with the triple combination group showing the highest level, indicating the occurrence of ICD (Figure 5G). Hematoxylin and eosin (H&E) staining confirmed extensive tumor cell damage in the GOX‐NG+ETX+αPD‐1 group. By contrast, GOX alone induced minimal histological changes. These findings were corroborated by Ki‐67 staining (reduced proliferation) and TUNEL staining (increased apoptosis). To further validate the dual metabolic and oxidative effects of the combination therapy in vivo, we conducted biochemical and histological analyses on tumor tissue. ROS staining and Oil Red O staining confirmed the highest levels of reactive oxygen species and lipid accumulation in the GOX‐NG+ETX+αPD‐1 group (Figure 5H and Figure S38). Quantification of intratumoral metabolites revealed significantly elevated H_2_O_2_ and reduced levels of GSH, ATP, glucose, lactate, and pyruvate, collectively indicating the establishment of a severely energy‐deficient and oxidative microenvironment (Figure 5I–N).

**FIGURE 6:**
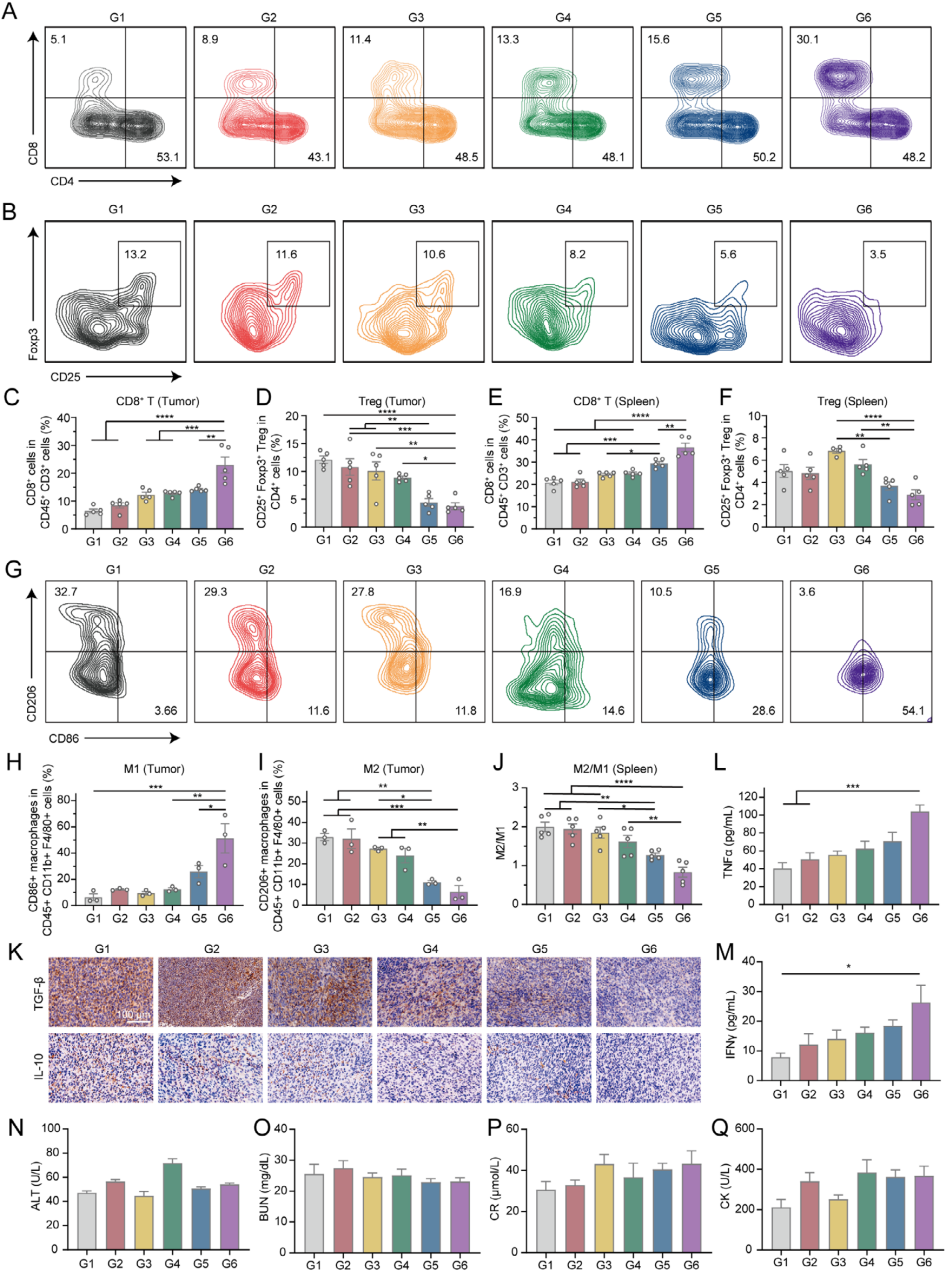
Antitumor immunity response including T cell infiltration and macrophage polarization of combination therapy with GOX‐NG + ETX and αPD‐1. (A) Flow cytometric images of the CD8^+^ and CD4^+^ T cells, gating on CD45^+^ CD3^+^ cells in tumor. (B) Flow cytometric images of the Foxp3^+^ CD25^+^ Tregs gating on CD4+ cells in the tumor. (C) The quantification of the CD8^+^ T cells gating on CD45^+^ CD3^+^ cells in the tumor (*n* = 5). (D) The quantification of the Foxp3^+^ CD25^+^ Tregs gating on CD4^+^ cells in tumor (*n* = 5). (E) The quantification of the CD8^+^ T cells gating on CD45^+^ CD3^+^ cells in the spleen (*n* = 5). (F) The quantification of the Foxp3^+^ CD25^+^ Tregs gating on CD4^+^ cells in the spleen (*n* = 5). (G) Flow cytometric images of the M1 macrophages CD86^+,^ and M2 macrophages CD206^+^ cells gating on CD45^+^ CD11b^+^ F4/80^+^ cells in tumor. (H‐I) The quantification of the M1 macrophages CD86^+^ and M2 macrophages CD206^+^ cells, gating on CD45^+^ CD11b^+^ F4/80^+^ cells in tumor (*n* = 3). (J) The quantification of the M2/M1 macrophages in the spleen (*n* = 4). (K) Representative immunohistochemical images of TGF‐β and IL‐10 of tumor tissue sections. (L,M) Serum levels of TNF‐α (*n* = 3) and IFN‐γ (*n* = 3). (N–Q) Serum levels of biochemical indicators, including ALT, BUN, CR, and CK (*n* = 4). All data are presented as mean ± SEM. *p < 0.05, **p < 0.01, ***p < 0.001 and ****p < 0.0001.

Generally speaking, under immunogenic cell death, dendritic cells (DCs) are activated and mature, presenting antigens and activating naive T cells. When further combined with ICB, it will trigger an intense adaptive immune response. To assess the immune activation, we analyzed T cell and macrophage populations in tumors and spleens using flow cytometry. The GOX‐NG+ETX+αPD‐1 group displayed the highest levels of CD8^+^ cytotoxic T cells and the lowest levels of regulatory T cells (Tregs, CD25^+^/Foxp3^+^) in CD4^+^ T cells, indicating enhanced cytotoxic T cell infiltration and successful reversal of local and systemic immune suppression (Figure 6A–F and Figures S39–S41). In addition, CD86^+^ pro‐inflammatory (M1) macrophages were significantly enriched, while CD206^+^ anti‐inflammatory (M2) macrophages were markedly reduced in both tumor and spleen samples (Figure 6G–J and Figure S42). Immunohistochemical analysis showed reduced expression of the immunosuppressive cytokines TGF‐β and IL‐10 in the tumor microenvironment (Figure 6K). At the same time, ELISA revealed elevated serum levels of the pro‐inflammatory cytokines TNF‐α and IFN‐γ, further indicating systemic immune activation (Figure 6L,M). Safety assessments—including body weight monitoring, hemolysis testing, complete blood counts, serum biochemistry, and histological evaluation of major organs—showed no signs of significant toxicity, confirming the good biocompatibility of the therapeutic regimen (Figure 6N–Q and Figures S43–S45).

### 2.9 Abscopal Effect: Suppression of Distant Tumors via Systemic Immune Activation

**FIGURE 7:**
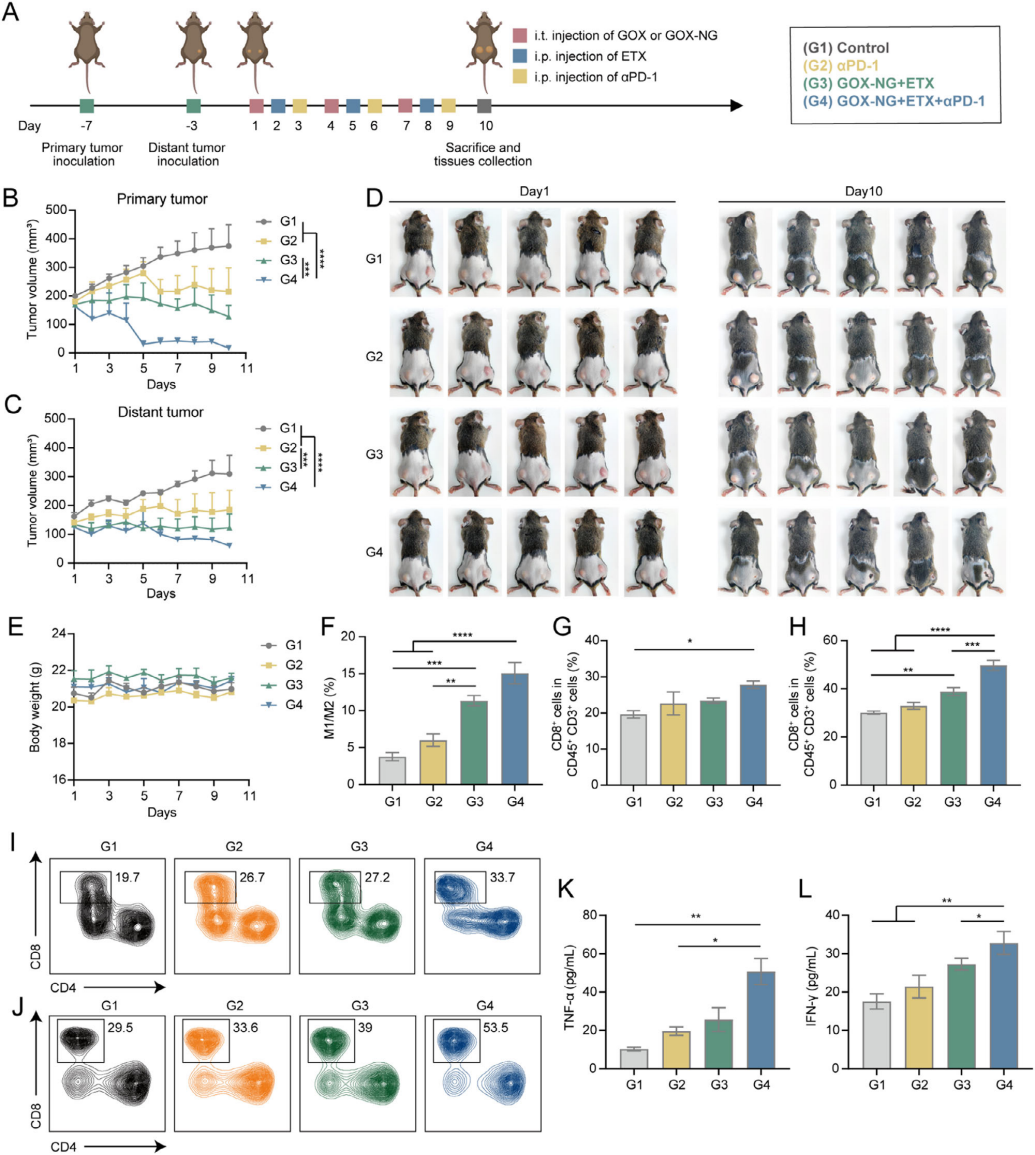
Abscopal effect of combination therapy with GOX‐NG + ETX and αPD‐1 checkpoint blockade. (A) Schematic illustration of the therapeutic schedule. (B) Daily average primary tumor volume in different treatment groups (*n* = 5). (C) Daily average distant tumor volume in different treatment groups (*n* = 5). (D) Bright‐field photographs of mice of different treatment groups before and after treatment. (E) Average body weight of mice in different treatment groups during treatment (*n* = 5). (F) The quantification of the M1/M2 macrophages in the distant tumor (*n* = 5). (G) The quantification of the CD8^+^ T cells gating on CD45^+^ CD3^+^ cells in the distant tumor (*n* = 5). (H) The quantification of the CD8^+^ T cells gating on CD45^+^ CD3^+^ cells in the spleen (*n* = 5). (I) Flow cytometric images of the CD8^+^ T cells, gating on CD45^+^ CD3^+^ cells in the distant tumor. (J) Flow cytometric images of the CD8^+^ T cells gating on CD45^+^ CD3^+^ cells in the spleen. (K) TNF‐α content in distant tumor (*n* = 3). (L) IFN‐γ content in distant tumor (*n* = 3). All data are presented as mean ± SEM. *p < 0.05, **p < 0.01, ***p < 0.001 and ****p < 0.0001.

To determine whether the enhanced immune response could suppress untreated distant tumors, we established a bilateral SCC7 tumor model in mice (Figure 7A). Using the same treatment protocol, we observed varying degrees of growth inhibition in the contralateral (non‐injected) tumors across all groups, with the most pronounced suppression occurring in the GOX‐NG+ETX+αPD‐1 group. Importantly, this regimen completely suppressed primary tumor growth while significantly inhibiting distant lesions—far surpassing the efficacy of either αPD‐1 monotherapy or GOX‐NG+ETX alone (Figure 7B–E). Immune profiling of the distant tumors and spleens revealed elevated CD8^+^ T cell levels and increased M1/M2 macrophage ratios (Figure 7F–J). Additionally, TNF‐α and IFN‐γ in distant tumor tissues were significantly elevated, indicating that local treatment of the primary tumor successfully induced a robust systemic antitumor immune response (Figure 7K,L). These findings highlight the potential of this strategy to generate meaningful abscopal effects, which is of great clinical significance for the treatment of metastatic disease.

## 3 Discussion

As a promising therapeutic enzyme in emerging cancer treatments, glucose oxidase (GOX) has been formulated into various delivery systems [47, 48, 49, 50]. However, challenges remain regarding its clinical translation, particularly in achieving consistent formulation quality and demonstrating long‐term in vivo safety [51]. In this study, we employed alginate as the nanogel scaffold material—an FDA‐approved pharmaceutical excipient commonly used in the clinic as a thickening agent, suspending agent, and disintegrant. Moreover, alginate‐based medical devices, such as dental impression materials, embolic agents, and wound dressings, have also been widely applied, underscoring its excellent biocompatibility and degradability [52, 53, 54]. Compared to inorganic or metal‐based carriers, our alginate nanogel platform offers superior biosafety [55, 56]. Relative to polymeric micelles or microspheres, it provides higher protein encapsulation efficiency and better preservation of protein structure and activity. Furthermore, unlike covalent conjugation or surface polymerization strategies that often involve complex processes and safety concerns due to residual monomers or crosslinkers, our system achieves straightforward and mild encapsulation, minimizing these risks [57, 58, 59, 60]. A particularly surprising finding in our work was the significantly enhanced cellular uptake, tumor penetration, and tissue retention of the GOX nanogel, despite its net negative charge. We speculate that this unexpected behavior may result from a combination of factors: increased particle size, improved structural stability, tissue adhesive properties conferred by catechol groups, and enhanced endocytosis efficiency. Further mechanistic studies, especially at the cellular trafficking level, are warranted to fully elucidate this phenomenon.

**FIGURE 8:**
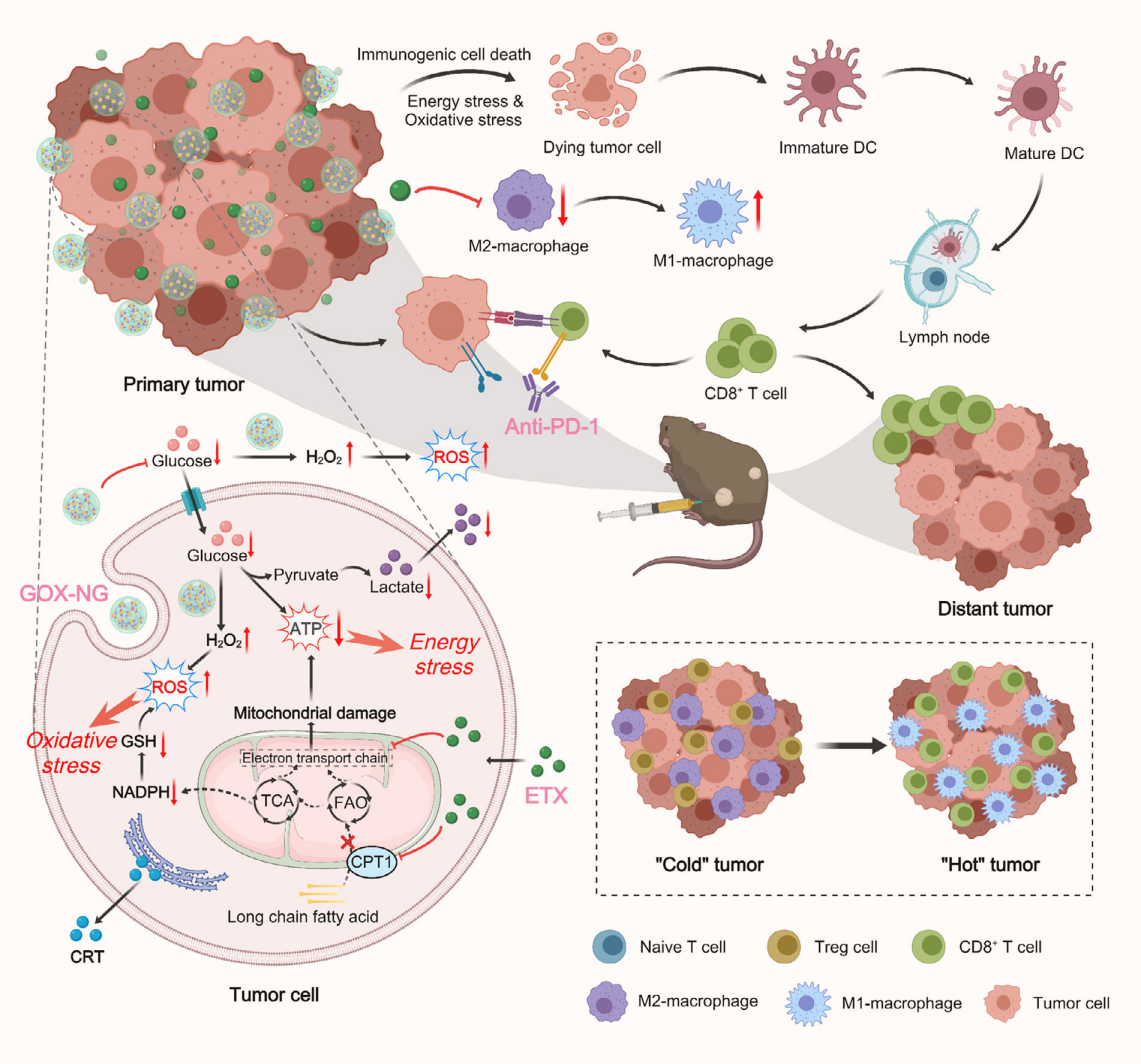
Schematic illustration of in situ tumor synergistic starvation–oxidation–immunotherapy based on glucose oxidase nanogel (GOX‐NG).

In the clinical context, the limited efficacy of monotherapies in treating highly resistant and immunosuppressive tumors highlights the urgent need for effective combination strategies. To date, the FDA has approved several combination regimens—ranging from chemotherapy–immunotherapy to dual immune checkpoint blockade—demonstrating the potential of rationally designed combinatorial approaches [61]. However, the success of combination therapy depends not just on drug co‐administration, but more importantly on the existence of mechanistic synergy—where the whole is greater than the sum of its parts. In this study, we focused on the compensatory interplay between glucose and lipid metabolism in cancer cells—a phenomenon governed by the Randle cycle. This inherent metabolic flexibility often limits the efficacy of single‐pathway inhibition. To overcome this, we proposed a dual inhibition strategy targeting both glycolysis and fatty acid oxidation (Figure 8). By temporally coordinating GOX‐NG and ETX administration, we achieved efficient suppression of both metabolic arms, resulting in significant ATP depletion and nutrient deprivation within the tumor. Simultaneously, the combined treatment induced elevated levels of reactive oxygen species (ROS), triggering oxidative stress and tumor ICD. Notably, recent studies have revealed that glucose deprivation can paradoxically promote tumor metastasis, highlighting the importance of immune regulation in starvation therapy [10, 62]. This insight prompted us to incorporate immune checkpoint inhibition into the treatment regimen. The resulting triple combination—GOX‐NG, ETX, and anti‐PD‐1 antibody—not only eliminated primary tumors but also induced potent systemic immune responses, leading to the suppression of untreated distant lesions. This starvation–oxidation–immunotherapy strategy forms a positive feedback loop: starvation sensitizes cells to oxidative stress, which in turn reshapes the immune microenvironment, rendering the tumor more responsive to immunotherapy (Figure 8). Currently, the response rate to immune checkpoint inhibitors like PD‐1 inhibitors is only 15%–20% in OSCC, and approximately 15%–35% of initial responders develop secondary resistance [63, 64]. Thus, this dynamic interplay of starvation–oxidation–immunotherapy strategy may convert “cold” tumors into “hot” tumors, thereby maximizing the therapeutic effect of immune checkpoint blockade.

It is worth noting that our current delivery approach—intratumoral injection—is most applicable to accessible tumors such as those in the oral cavity, breast, and skin. For deep‐seated or hematological malignancies, future integration with molecular targeting technologies or image‐guided delivery methods (e.g., endoscopic or interventional approaches) may broaden the clinical applicability of this strategy.

## 4 Conclusion

In this proof‐of‐concept study, we developed a GOX nanogel system based on catechol‐functionalized alginate. The resulting formulation exhibits excellent injectability, high structural stability, enhanced tumor penetration, and prolonged tissue retention. These features collectively enable spatiotemporal coordination of dual metabolic inhibition targeting glucose and lipid metabolism within tumors. Mechanistically, GOX‐NG synergizes with the fatty acid oxidation inhibitor etomoxir to suppress glycolysis and β‐oxidation simultaneously, leading to nutrient starvation, elevated ROS generation, disruption of redox homeostasis and ICD of tumor. Moreover, this combination reprograms the tumor immune microenvironment, particularly by inhibiting M2 macrophage polarization and enhancing pro‐inflammatory signaling. When integrated with immune checkpoint blockade, the strategy achieved remarkable therapeutic outcomes in an oral squamous cell carcinoma model: 100% survival, complete tumor regression in 60% of animals, and suppression of distant metastases via systemic immune activation. These results underscore the clinical potential of this integrated starvation–oxidation–immunotherapy strategy for treating both primary and metastatic tumors. Beyond GOX, this nanogel‐based spatiotemporal combination platform may also be applicable to other protein‐based anticancer agents, such as amino acid–targeting enzymes like L‐asparaginase, improving their stability, tumor uptake, and antitumor efficacy, while mitigating off‐target toxicity. Overall, this work offers a versatile and translationally relevant approach for advancing multi‐mechanism cancer therapies centered on metabolic modulation.

## 5 Methods

### 5.1 Synthesis and Characterization of Cat‐Alg

Sodium alginate (1 g, MACKLIN) was dissolved in 100 mL of PBS (50 mM, pH 5.5). 1‐(3‐Dimethylaminopropyl)‐3‐ethylcarbodiimide (EDC) (0.96 g, MACKLIN) and N‐Hydroxysuccinimide (NHS) (0.68 g, MACKLIN) were added into the solution in nitrogen atmosphere. After stirring for 45 min, dopamine hydrochloride (1.92 g, RHAWN) was added into the solution. The mixture was sealed and stirred overnight. After that, the reaction product was dialyzed against deionized water (pH 4.0) in a dialysis bag (MWCO = 3500) for two days. Finally, the solution was lyophilized and the grey sodium alginate grafted with catechol groups (Cat‐Alg) was obtained. The ^1^H NMR spectra were recorded to verify its successful synthesis. Catechol group has a characteristic UV absorption peak at 280 nm, and the grafting rate of catechol in Cat‐Alg is 177.2 µmol/1 g Cat‐Alg by a UV‐vis spectrophotometer.

### 5.2 Optimization of Oxidation Conditions for Cat‐Alg Catechol Groups

Adding the oxidizing agent NaIO_4_ (MACKLIN) to Cat‐Alg can oxidize the catechol group to the structure of O‐diquinone, and the quinone group has the ability to form irreversible covalent bonds with sulfhydryl groups and amino groups. NaIO_4_ was added to dopamine hydrochloride solution or Cat‐Alg solution for oxidation experiments, and the UV absorption changes of the solutions were detected at different time points. The characteristic absorption peak of O‐diquinone was found at 460–475 nm. Due to the instability of the structure of O‐diquinone, the peak value first increased and then decreased with time. The concentration of Cat‐Alg solution (5, 10, and 20 mg mL^−1^) and the molar ratio of Cat/IO_4_^−^ (3:1, 2:1, 1:1, 1:2, 1:3) were adjusted to detect the UV absorption of Cat‐Alg within 48 h.

### 5.3 Preparation of GOX Nanogel (GOX‐NG)

O‐diquinone can react with mercaptans and amines in proteins by Michael addition or Schiff base reaction. According to the optimized oxidation conditions, Cat‐Alg (6 mg) was dissolved in 1 mL PBS, and 200 µL NaIO_4_ solution (2.27 mg mL^−1^) was added. After 4 h of oxidation, according to the mass ratio of GOX/Cat‐Alg (1:2, 1:3, 1:5), different amounts of glucose oxidase (GOX) (3, 2 or 1.2 mg, Shanghai Yuanye) were added to the system. After 48 h of reaction at 37°C, the solution was ultrafiltered (MWCO = 10 kDa) for 3 times to remove NaIO_4_, and three different proportions of GOX‐NG were obtained.

### 5.4 Characterization of GOX‐NG

The hydraulic diameters, PDI, size distribution, and zeta potentials were detected by dynamic light scattering (ZETASIZER NANO ZSP). The morphology images of GOX‐NG were captured by a transmission electron microscopy (HRTEM‐JEM‐F200). The molecular weight change of GOX‐NG was verified from the sodium dodecyl sulfate‐polyacrylamide gel electrophoresis (SDS‐PAGE).

### 5.5 Assay for pH, Glucose, and H_2_O_2_ Changes Catalyzed by GOX‐NG

For pH changes, 20 µL of glucose (90 mg mL^−1^) was added to 2 mL of GOX or GOX‐NG solution (GOX concentration = 100 µg mL^−1^), and the pH of the solution was determined with a pH meter at 0, 0.5, 1, 2, 3, and 4 h. For the change of glucose, 5 µL of glucose (90 mg mL^−1^) was added to 0.5 mL GOX or GOX‐NG solution (GOX concentration = 100 µg mL^−1^), and the content of glucose in the solution was determined by a Glucose Content Assay Kit (Solarbio) at 0, 0.5, 1, 2, 3, and 4 h. For the change of H_2_O_2_, 3, 2.4, 1.8, 1.2, or 0.6 µL of glucose (90 mg mL^−1^) was added to 0.3 mL GOX or GOX‐NG solution (GOX concentration = 100 µg mL^−1^). After reaction at 37°C for 4 h, the content of H_2_O_2_ in the solution was detected with a Hydrogen Peroxide Assay Kit (Beyotime).

### 5.6 Enzyme Activity Assay

The relative enzyme activities of GOX and GOX‐NG (GOX concentration = 100 µg mL^−1^) were detected by a Glucose Oxidase Activity Assay Kit (Solarbio). To evaluate the thermal stability of GOX‐NG, 1 mL of GOX or GOX‐NG solution (GOX concentration = 100 µg mL^−1^) was placed in a 60°C water bath, and changes in enzyme activity were detected at different time points. To evaluate the enzymatic stability of GOX‐NG, 25 µL of Protease K solution (20 mg mL^−1^) (Beyotime) was added to 1 mL of GOX or GOX‐NG solution (GOX concentration = 100 µg mL^−1^) at 37°C and changes in enzyme activity were detected at different time points.

### 5.7 Cell and MCTS Culture

Murine oral cancer cell line SCC7 cells, murine breast cancer cell line 4T1 cells, and human breast cancer cell line MDA‐MB‐231 cells were cultured with DMEM (Wuhan Pricella) containing 10% fetal bovine serum (FBS) (Wuhan Pricella) and 1% penicillin and streptomycin (Wuhan Pricella) at 37°C in a temperature incubator containing 5% CO_2_. The multicellular tumor spheroids (MCTS) of SCC7 cells were cultured with DMEM by an ultra‐low adhesion U‐bottom 96‐well plate (2 × 10^3^ cells per well).

### 5.8 Cellular Uptake of GOX‐NG

GOX and GOX‐NG were labeled with Fluorescein isothiocyanate (FITC) (MACKLIN) (molar ratio of GOX: FITC = 1:20, pH 8.5). SCC7 cells seeded in 12‐well plate (2 × 10^5^ cells per well) were incubated with FITC‐GOX or FITC‐GOX‐NG (GOX concentration = 25, 50, or 100 µg mL^−1^) for 2 h. Then the cells were tested by flow cytometry. In addition, SCC7 cells seeded confocal dishes (2 × 10^5^ cells) were incubated with FITC‐GOX or FITC‐GOX‐NG (GOX concentration = 25 µg mL^−1^) for 2 h. Then the cells were stained with Hoechst 33342 (Beyotime) and Rhodamine labeled phalloidine (Solarbio), and were observed by a laser confocal microscope.

### 5.9 In Vitro Permeability in MCTS

SCC7 MCTS were incubated with FITC‐GOX or FITC‐GOX‐NG (GOX concentration = 25 µg mL^−1^) for 2, 5, 10, and 24 h. Then the MCTS were stained with Hoechst 33342 and observed by a laser confocal microscope.

### 5.10 In Vivo Bio‐Distribution of GOX‐NG

GOX and GOX‐NG were labeled with Sulfo‐Cy7‐SE (MACKLIN) (molar ratio of GOX: Cy7 = 1:20, pH 8.5). SCC7 tumor‐bearing mice were divided into two groups (*n* = 3). The mice were treated with Cy7‐GOX or Cy7‐GOX‐NG by intratumor injection. The fluorescence distribution in mice was observed by a small animal imaging system (IVIS Lumina Series III) at 0, 0.5, 3, 6, 12, and 24 h after injection. 24 h after injection, the mice were dissected and fluorescence quantification of the isolated organs and tumors was performed.

### 5.11 In Vivo Glucose Consumption of GOX‐NG

GOX‐NG was administered via intratumoral injection into tumor‐bearing mice. Tumor tissues were harvested at 6, 12, 24, and 48 h, and the glucose content within the tumors was determined by tissue homogenization using a Glucose Content Assay Kit (Solarbio). The data for 0 h represent the glucose content in tumor tissues from untreated mice, which served as the control group.

### 5.12 Cytotoxicity Assay

To evaluate the cytotoxicity of GOX, GOX‐NG or Etomoxir (ETX) (MedChemExpress), SCC7 cells or 4T1 cells seeded in 96‐well plate (5 × 10^3^ cells per well) were incubated with GOX, GOX‐NG, GOX‐NG+ETX (ETX concentration = 100, 200, and 500 µM, GOX concentration = 0, 8, 16, 32, 48, 64, and 80 ng mL^−1^) for 24 h. And the cytotoxicity was preformed using a CCK8 kit (BIOREGEN).

To verify that glucose depletion could sensitize cancer cells to ETX, SCC7 cells were first seeded in 96‐well plate (5 × 10^3^ cells per well) in DMEM containing glucose for 12 h. After complete attachment, cells were incubated with ETX (0, 50, 100, 200, and 500 µM) for 24 h in DMEM with or without glucose. Subsequently, cell viability was measured using a CCK8 kit.

Furthermore, another experiment was designed to verify that the glucose depletion induced by GOX‐NG also sensitized cancer cells to ETX when GOX‐NG was used in combination with ETX, resulting in enhanced cell cytotoxicity. SCC7 cells were seeded in three 96‐well plates (denoted as well as plate A, B, and C) and cultured for 12 h until the cells adhered. Subsequently, cells in plate A were co‐cultured with GOX‐NG+ETX, and cells in plates B and C were co‐cultured with GOX‐NG. After 24 h, the medium in plate A was discarded, and the entire medium in plate C was transferred to plate A for continued incubation. In plate B, incubation was continued after ETX was added to the medium. After an additional 24 h, cell viability was measured using a CCK8 kit. (ETX concentration = 50 or 100 µM, GOX concentration = 0, 8, 16, 32, 48 ng mL^−1^).

### 5.13 Calculation of Combination Index (CI)

SCC7 cells were seeded in 96‐well plate (5 × 10^3^ cells per well) and incubated with 3 drug groups at a series of concentrations for 24 h: 1, GOX‐NG; 2, ETX; 3, GOX‐NG + ETX (Fixed concentration ratio of GOX‐NG to ETX = 1: 5). After that the cell viability was measured using a CCK8 kit. Finally, the dose‐response curve was used to fit the data of single drug and combination drug, and the IC50 values of the three drug groups were obtained. The combination index was calculated by the following formula when GOX‐NG and ETX were used in combination:

$$\begin{array}{cc} \mathrm{CI} & ={\left(\mathrm{IC}50_{\left(\mathrm{GOX}-\mathrm{NG}\right)}\right)}_{\mathrm{Combined}}/{\left(\mathrm{IC}50_{\left(\mathrm{GOX}-\mathrm{NG}\right)}\right)}_{\mathrm{Single}}\\ & \;+{\left(\mathrm{IC}50_{\left(\mathrm{ETX}\right)}\right)}_{\mathrm{Combined}}/{\left(\mathrm{IC}50_{\left(\mathrm{ETX}\right)}\right)}_{\mathrm{Single}} \end{array}$$

### 5.14 Live Cell Staining

SCC7 cells seeded in 96‐well plate (5 × 10^3^ cells per well) were incubated with GOX, GOX‐NG, GOX‐NG+ETX (GOX concentration = 40 ng mL^−1^, ETX concentration = 100, 200, and 500 µM) for 24 h. After that SCC7 cells were stained with Fluorescein diacetate (FDA) (RHAWN) for 15 min and observed using a fluorescence microscope.

SCC7 cells were first seeded in 96‐well plate (5 × 10^3^ cells per well) in DMEM containing glucose for 12 h. After complete attachment, cells were incubated with ETX (0, 50, 100, 200, and 500 µM) for 24 h in DMEM with or without glucose. After that SCC7 cells were stained with FDA for 15 min and observed using a fluorescence microscope.

### 5.15 Cell Apoptosis

SCC7 cells seeded in 6‐well plate (6 × 10^5^ cells per well) were incubated with PBS, ETX, GOX, GOX‐NG, GOX‐NG+ETX (GOX concentration = 40 ng mL^−1^, ETX concentration = 200 or 500 µM) for 24 h. After that SCC7 cells were stained with AnnexinV Alexa Fluor488/PI kit (Solarbio) and tested by flow cytometry.

### 5.16 Cellular Energy Metabolism

The experiments were conducted in a Seahorse XF96 Analyzer. SCC7 cells were plated at 1 × 10^4^ cells per well for 12 h. After complete attachment, cells were incubated with ETX, GOX‐NG, GOX‐NG+ETX (GOX concentration = 40 ng mL^−1^, ETX concentration = 200 µM) for 24 h. On the day of the assay, the medium was replaced with detection buffer composed of XF DMEM medium supplemented with sodium pyruvate (1 mM), glutamine (2 mM), and glucose (10 mM). For XF Cell Mito Stress Test Assay, respiration was measured under basal conditions as well as after injection of drug (ETX, GOX‐NG or GOX‐NG+ETX), 0.5 µM oligomycin,1 µM FCCP, followed by 0.5 µM rotenone/antimycin A. For XF Glycolysis Stress Test Assay, respiration was measured under basal conditions as well as after injection of drug (ETX, GOX‐NG or GOX‐NG+ETX), 0.5 µM rotenone/antimycin A, and 50 mM 2‐DG.

### 5.17 Glucose Content Assay

SCC7 cells seeded in 6‐well plate (6 × 10^5^ cells per well) were incubated with PBS, ETX, GOX, GOX‐NG, GOX‐NG+ETX (GOX concentration = 40 ng mL^−1^, ETX concentration = 200 or 500 µM) for 24 h. After that, the medium supernatant was collected, and the glucose content was tested by a Glucose Content Assay Kit (Solarbio).

### 5.18 Lactate Content Assay

SCC7 cells seeded in 6‐well plate (6 × 10^5^ cells per well) were incubated with PBS, ETX, GOX, GOX‐NG, GOX‐NG+ETX (GOX concentration = 40 ng mL^−1^, ETX concentration = 200 or 500 µM) for 24 h. After that the medium supernatant was collected and the lactate content was tested by a Lactic Acid (LA) Content Assay Kit (Solarbio).

### 5.19 ATP Content Assay

SCC7 cells seeded in 6‐well plate (6 × 10^5^ cells per well) were incubated with PBS, ETX, GOX, GOX‐NG, GOX‐NG+ETX (GOX concentration = 40 ng mL^−1^, ETX concentration = 200 or 500 µM) for 24 h. After that the cells were collected and the ATP content in cells was tested by Enhanced ATP Assay Kit (Beyotime).

### 5.20 NADPH Content Assay

SCC7 cells seeded in 6‐well plate (6 × 10^5^ cells per well) were incubated with PBS, ETX, GOX, GOX‐NG, GOX‐NG+ETX (GOX concentration = 40 ng mL^−1^, ETX concentration = 200 or 500 µM) for 24 h. After that the cells were collected and the NDADPH content in cells was tested by CoenzymeII NADP(H) Content Assay Kit (Solarbio).

### 5.21 Lipid Droplet Assay

SCC7 cells seeded in confocal dishes (3 × 10^5^ cells per well) were incubated with PBS, ETX, GOX, GOX‐NG, GOX‐NG+ETX (GOX concentration = 40 ng mL^−1^, ETX concentration = 200 or 500 µM) for 24 h. After that, the cells were stained using Lipid Droplets Green Fluorescence Assay Kit with BODIPY 493/503 (Beyotime) and observed by CLSM.

### 5.22 ROS Generation Assay

SCC7 cells seeded in 12‐well plate or confocal dishes (3 × 10^5^ cells per well) were incubated with PBS, ETX, GOX, GOX‐NG, GOX‐NG+ETX (GOX concentration = 40 ng mL^−1^, ETX concentration = 200 or 500 µM) for 24 h. After that the cells were stained using Reactive Oxygen Species Assay Kit (Solarbio) and tested by flow cytometry or CLSM.

### 5.23 GSH Content Assay

SCC7 cells seeded in 6‐well plate (6 × 10^5^ cells per well) were incubated with PBS, ETX, GOX, GOX‐NG, GOX‐NG+ETX (GOX concentration = 40 ng mL^−1^, ETX concentration = 200 or 500 µM) for 24 h. After that, the cells were collected and the GSH content in the cells was tested by the Reduced Glutathione (GSH) Content Assay Kit (Solarbio).

### 5.24 Extraction and Polarization of BMDM

To obtain bone marrow‐derived macrophages (BMDM), the femur of balb/c mice (female, 18–22 g) was isolated and irrigated with a syringe. After cracking the red blood cells, the obtained bone marrow cells were centrifuged and inoculated into a cell culture dish. On the second day, the cells were stimulated with macrophage colony‐stimulating factor (M‐CSF) (ABclonal) (20 ng mL^−1^) for 7 days. During this period, fresh medium containing M‐CSF was replaced on the 3rd and 5th days to obtain unpolarized M0 macrophages.

M0 macrophages were inoculated on 6‐well plates and incubated with PBS, ETX, ETX+GOX‐NG (GOX concentration = 80 ng mL^−1^, ETX concentration = 200 µM) for 24 h. IL‐4 (ABclonal) (20 ng mL^−1^) was added into the medium and the cells continued to be cultured for 24 h. The cells were then collected and incubated with PE‐F4/80^+^, APC‐CD206^+^ and PE‐Cy7‐CD163^+^ antibodies, and the proportion of M2 macrophages was measured by flow cytometry. The flow cytometry antibody information is shown in Table S2.

### 5.25 RNA Isolation and qPCR Analysis for BMDM

M0 macrophages were inoculated on 6‐well plates and incubated with PBS, ETX, ETX+GOX‐NG (GOX concentration = 80 ng mL^−1^, ETX concentration = 200 µM) for 24 h. IL‐4 (20 ng mL^−1^) was added to the medium, and the cells continued to be cultured for 24 h. After that the cells were collected using cell scrapers and centrifuged. RNA was isolated by FastPure Cell/Tissue Total RNA Isolation Kit (Vazyme). Then cDNA was synthesized using Evo M‐MLV RT Premix for qPCR (Accurate). SYBR Green Premix Pro Taq HS qPCR Kit (Accurate) was used for quantitative RT‐PCR by QuantStudio 3 System (Thermo Scientific). The primer sequences are shown in Table S1.

### 5.26 RNA Sequencing Assay

SCC7 cells seeded in 6‐well plate (6 × 10^5^ cells per well) were incubated with PBS, ETX, GOX‐NG, GOX‐NG+ETX (GOX concentration = 40 ng mL^−1^, ETX concentration = 200 µM) for 24 h. RNA‐seq analysis was conducted on Dr. Tom system (BGI‐Shenzhen, China). Differential expression analysis was performed using DESeq2 with default parameters: |log_2_FoldChange| > 1 and Q‐value < 0.05. Up‐ and down‐regulated genes were highlighted on the volcano plot based on these thresholds, and the heatmap was normalized using TPM z‐score by row.

### 5.27 Animal Models

C3H mice (female, 6–8 weeks) were purchased from Vital River Laboratory Animal Technology (Beijing, China). The SCC7 oral cancer model was established by subcutaneous injection with 5 × 10^6^ SCC7 cells in 100 µL of serum‐free DMEM at the right side of the back. All the experiments on animals were conducted according to the guidelines of Laboratory Animal Welfare Ethics Committee of Peking University.

### 5.28 Maximum Tolerated Dose (MTD) Measurement

SCC7 tumor‐bearing mice were randomly grouped (*n* = 3) and treated with GOX (2, 4, or 6 mg kg^−1^) or GOX‐NG (GOX concentration = 4, 8, 12, or 16 mg kg^−1^) by intratumoral administration. The weight of the mice was measured daily for a week after administration. The MTD of GOX or GOX‐NG was defined as the maximum dose that did not cause over 10% weight loss compared with the initial weight.

### 5.29 In Vivo Antitumor Efficacy of GOX‐NG+ETX Combined with Immunotherapy

SCC7 cells (5 × 10^6^ cells) in 100 µL of serum‐free DMEM were subcutaneously injected at the right back side of C3H female mice. When the tumor volume reached 120 mm^3^, the mice were randomly divided into six groups (*n* = 5): 1, Control; 2, αPD‐1 (BioXcell); 3, GOX; 4, GOX‐NG; 5, GOX‐NG+ETX; 6, GOX‐NG+ETX+αPD‐1. (GOX concentration = 2 mg kg^−1^, GOX‐NG concentration = 8 mg kg^−1^, ETX concentration = 20 mg kg^−1^, αPD‐1 concentration = 10 mg kg^−1^). The treatment consisted of 3 cycles. On the first day of each cycle, GOX or GOX‐NG was injected intratumorally. On the second day, ETX was injected intraperitoneally. On the third day, αPD‐1 was injected intraperitoneally. The weight and tumor volume of the mice were measured and recorded daily. The following formula was used to calculate the tumor volume:

$$\mathrm{Tumor}\;\mathrm{volume}\;\left(V\right)=\left(\mathrm{Length}\times \mathrm{Widt}h^{2}\right)\times 0.5$$

The mice were euthanized on the 10th day. Tumor and organs (heart, liver, spleen, lung, and kidney) of mice were dissected and made into tissue slices. The contents of hydrogen peroxide, GSH, ATP, glucose, lactic acid and pyruvate in the tumor were detected by the corresponding kit (Hydrogen Peroxide Content Assay Kit (Solarbio), Reduced Glutathione Content Assay Kit (Solarbio), Enhanced ATP Assay Kit (Beyotime), Glucose Content Assay Kit (Solarbio), Lactic Acid Content Assay Kit (Solarbio), Pyruvate Content Assay Kit (Solarbio)) after grinding the tumor tissue in the ice bath.

Mouse serum was also obtained by extracting blood from eyeballs after treatment. The serum concentrations of TNF‐α and IFN‐γ were analyzed using ELISA kits (Solarbio). Biochemical markers in serum including ALT, BUN, CR, and CK were analyzed by Servicebio.

Another survival experiment followed the same protocol, with the mice's survival monitored and recorded after the end of treatment. The mice were considered dead when the tumor reached 600 mm^3^.

### 5.30 Abscopal Effect

SCC7 cells (3 × 10^6^ cells) in 100 µL of serum‐free DMEM were subcutaneously injected at the right back side of C3H female mice on −7 day, denoted as primary tumor. SCC7 cells (3 × 10^6^ cells) in 100 µL of serum‐free DMEM were subcutaneously injected at the left back side of mice on −3 day, denoted as distant tumor. The mice were randomly divided into 4 groups (*n* = 5) on 0 day: 1, Control; 2, αPD‐1; 3, GOX‐NG+ETX; 4, GOX‐NG+ETX+αPD‐1. (GOX‐NG concentration = 8 mg kg^−1^, ETX concentration = 20 mg kg^−1^, αPD‐1 concentration = 10 mg kg^−1^). The treatment consisted of 3 cycles. On the first day of each cycle, GOX‐NG was injected intratumorally. On the second day, ETX was injected intraperitoneally. On the third day, αPD‐1 was injected intraperitoneally. Both primary tumor and distant tumor volumes of the mice were measured and recorded daily. The mice were euthanized on the 10th day. Tumor and organs (heart, liver, spleen, lung, and kidney) of mice were dissected and made into tissue slices for further study.

### 5.31 Flow Cytometric Analysis

For the analysis of T lymphocytes and macrophages in vivo, the tumor and spleen of mice were obtained, and the tissue was cut up, digested by collagenase, and filtered through a filter to obtain a single‐cell suspension. The samples were then stained with surface or intracellular antigens using flow antibodies. The used primary antibodies and dilution rate were indicated in Table S2. The stained cells were tested using a full‐spectrum flow analyzer (Sony) and the data were analyzed using Flowjo software.

### 5.32 Immunohistochemistry and Immunofluorescence

Dewaxing and antigenic repair of sections were performed using an automatic dewaxing and antigen repair apparatus. For immunohistochemical staining, tissue sections were stained according to the instructions of UltraSensitive SP IHC Kit (FUZHOU MAIXIN BIOTECH) and Rat two‐step assay kit (ZSGB‐BIO), including ki67, TGF‐β (ABclonal), and IL10 (abcam). For immunofluorescence staining, tissue sections were stained according to the instructions of One Step TUNEL Apoptosis Assay Kit (Beyotime) and TSAPLus fluorescent three‐standard four‐color dyeing kit (Servicebio). Slides were scanned with the use of a digital pathology slide scanner.

### 5.33 Hemolysis Assay

200 µL defibrillated sheep blood was added to 1 mL of GOX‐NG at different concentrations. The negative control was normal saline and the positive control was water. After incubating at 37°C for 30 min, the samples were centrifuged at 2500 rpm for 5 min. The supernatant was taken for absorbance determination (OD 540 nm). The hemolysis rate is calculated as follows:

$$\mathrm{Hemolysis}\;\mathrm{rate}\;\left(\%\right)=\mathrm{Abs}\;\left(\mathrm{sample}\right)/\mathrm{Abs}\;\left(\mathrm{positive}\right)\times 100\%$$

### 5.34 Blood Routine Assay

SCC7 tumor‐bearing C3H mice were divided into 3 groups (*n* = 4). Group 1 was the control group, and the other two groups were injected with GOX and GOX‐NG, respectively. Blood was collected on day 1 and day 7 for routine blood tests.

### 5.35 Statistical Analysis

All data are presented as mean ± SEM. The comparison between the two groups was performed using an unpaired two‐tailed *t* test. For comparisons between three or more independent groups, one‐way analysis of variance (ANOVA) was performed using the Tukey multiple comparison test. Statistical analysis was performed by GraphPad Prism 7. p values: *p < 0.05, **p < 0.01, ***p < 0.001, and ****p < 0.0001.

## Author Contributions

**Weiping Gao**: writing – review and editing. **Jianan Zheng**: investigation, visualization. **Yixuan Fu**: software, validation, **Fei Duan**: writing – review and editing, project administration. **Zijian Gong**: data curation, formal analysis. **Qinzhi Su**: validation, data curation. **Binglin Chen**: methodology, visualization, project administration. **Yuan Gao**: conceptualization, investigation, methodology, software, data curation, writing – original draft. **Xinyu Liu**: conceptualization, methodology, resources, writing – review and editing, writing – original draft, supervision, funding acquisition. **Xuliang Deng**: funding acquisition, writing – review and editing, supervision. **Jinqi Wei**: writing – review and editing, supervision. **Xiao Sang**: validation, formal analysis, software.

## Funding

This research was supported by the National Key R&D Program of China (2022YFC2405900, 2022YFC2405903); NSFC/China (82202327, 82402194); Beijing Natural Science Foundation (L242126); and Clinical Medicine Plus X‐Young Scholars Project, Peking University, the Fundamental Research Funds for the Central Universities (PKU2024LCXQ018).

## Conflicts of Interest

The authors declare no conflicts of interest.

## Supporting information




**Supporting File**: advs76109‐sup‐0001‐SuppMat.docx.

## Data Availability

The data that support the findings of this study are available in the supplementary material of this article.

## References

[1] I. Martínez‐Reyes and N. S. Chandel , “Cancer Metabolism: Looking Forward,” Nature Reviews Cancer 21 (2021): 669–680, 10.1038/s41568-021-00378-6. 34272515

[2] Q.‐Y. Duan , Y.‐X. Zhu , B.‐H. Shan , et al., “Reallocating Cell Respiration Substrates for Cancer Therapy Using a Metabolism Regulator With an Intermembrane‐Translocatable Accessory,” Advanced Functional Materials 33 (2023): 2213636, 10.1002/adfm.202213636.

[3] X. Wang , L. Wang , Q. Hao , M. Cai , X. Wang , and W. An , “Harnessing Glucose Metabolism With Nanomedicine for Cancer Treatment,” Theranostics 14 (2024): 6831–6882, 10.7150/thno.100036. 39479443 PMC11519798

[4] X. Bian , R. Liu , Y. Meng , D. Xing , D. Xu , and Z. Lu , “Lipid Metabolism and Cancer,” Journal of Experimental Medicine 218 (2021): 20201606, 10.1084/jem.20201606. PMC7754673 33601415

[5] R. Liu , J.‐H. Lee , J. Li , et al., “Choline Kinase Alpha 2 Acts as a Protein Kinase to Promote Lipolysis of Lipid Droplets,” Molecular Cell 81 (2021): 2722–2735.e9, 10.1016/j.molcel.2021.05.005. 34077757

[6] Y. Ma , S. M. Temkin , A. M. Hawkridge , et al., “Fatty Acid Oxidation: An Emerging Facet of Metabolic Transformation in Cancer,” Cancer Letters 435 (2018): 92–100, 10.1016/j.canlet.2018.08.006. 30102953 PMC6240910

[7] Y. Liu , Y. Zhao , H. Song , et al., “Metabolic Reprogramming in Tumor Immune Microenvironment: Impact on Immune Cell Function and Therapeutic Implications,” Cancer Letters 597 (2024): 217076, 10.1016/j.canlet.2024.217076. 38906524

[8] K. Huang , Y. Han , Y. Chen , H. Shen , S. Zeng , and C. Cai , “Tumor Metabolic Regulators: Key Drivers of Metabolic Reprogramming and the Promising Targets in Cancer Therapy,” Molecular Cancer 24 (2025): 7, 10.1186/s12943-024-02205-6. 39789606 PMC11716519

[9] Y.‐W. Du , Z.‐R. Cai , X.‐T. Duan , et al., “Lipid Metabolism Reprogramming Shapes the Immune Landscape in the Tumor Microenvironment,” Cellular & Molecular Immunology 23 (2026): 457–470, 10.1038/s41423-026-01411-0. 41946907 PMC13128921

[10] D. Guo , Y. Tong , X. Jiang , et al., “Aerobic Glycolysis Promotes Tumor Immune Evasion by hexokinase2‐mediated Phosphorylation of IκBα,” Cell Metabolism 34 (2022): 1312–1324.e6, 10.1016/j.cmet.2022.08.002. 36007522

[11] C.‐Y. Wu , C.‐X. Huang , X.‐M. Lao , et al., “Glucose Restriction Shapes Pre‐metastatic Innate Immune Landscapes in the Lung Through Exosomal TRAIL,” Cell 188 (2025): 5701–5716.e19, 10.1016/j.cell.2025.06.027. 40669460

[12] P. J. Randle , P. B. Garland , C. N. Hales , and E. A. Newsholme , “The Glucose Fatty‐Acid Cycle Its Role In Insulin Sensitivity And The Metabolic Disturbances Of Diabetes Mellitus,” Lancet 281 (1963): 785–789. 10.1016/s0140-6736(63)91500-9 13990765

[13] A. A. Challa , “The Enduring Relevance of the Randle Cycle,” Nature Reviews Endocrinology 21 (2025): 269–269, 10.1038/s41574-025-01092-1. 39972151

[14] Y. Meng , D. Guo , L. Lin , et al., “Glycolytic Enzyme PFKL Governs Lipolysis by Promoting Lipid Droplet–Mitochondria Tethering to Enhance β‐Oxidation and Tumor Cell Proliferation,” Nature Metabolism 6 (2024): 1092–1107, 10.1038/s42255-024-01047-2. 38773347

[15] A. S. Rambold , S. Cohen , and J. Lippincott‐Schwartz , “Fatty Acid Trafficking in Starved Cells: Regulation by Lipid Droplet Lipolysis, Autophagy, and Mitochondrial Fusion Dynamics,” Developmental Cell 32 (2015): 678–692, 10.1016/j.devcel.2015.01.029. 25752962 PMC4375018

[16] J. Zhang , Y. Yin , J. Zhang , et al., “Suppression of Energy Metabolism in Cancer Cells With Nutrient‐sensing Nanodrugs,” Nano Letters 22 (2022): 2514–2520, 10.1021/acs.nanolett.2c00356. 35285648

[17] S. H. Khatami , O. Vakili , N. Ahmadi , et al., “Glucose Oxidase: Applications, Sources, and Recombinant Production,” Biotechnology and Applied Biochemistry 69 (2022): 939–950, 10.1002/bab.2165. 33840140

[18] C. Wang , J. Yang , C. Dong , and S. Shi , “Glucose Oxidase‐Related Cancer Therapies,” Advanced Therapeutics 3 (2020): 2000110, 10.1002/adtp.202000110.

[19] Y. Zhang , S. Jiang , J. Lin , and P. Huang , “Antineoplastic Enzyme as Drug Carrier With Activatable Catalytic Activity for Efficient Combined Therapy,” Angewandte Chemie International Edition 61 (2022): 202208583, 10.1002/anie.202208583. 35848681

[20] K. Song , J. Ming , B. Tao , et al., “Emerging Glucose Oxidase‐delivering Nanomedicines for Enhanced Tumor Therapy,” Journal of Controlled Release 381 (2025): 113580, 10.1016/j.jconrel.2025.02.076. 40024341

[21] M. Wang , D. Wang , Q. Chen , C. Li , Z. Li , and J. Lin , “Recent Advances in Glucose‐Oxidase‐Based Nanocomposites for Tumor Therapy,” Small 15 (2019): 1903895, 10.1002/smll.201903895. 31747128

[22] X. Ma , S.‐J. Li , Y. Liu , et al., “Bioengineered Nanogels for Cancer Immunotherapy,” Chemical Society Reviews 51 (2022): 5136–5174, 10.1039/D2CS00247G. 35666131

[23] Z. Zhao , Q. Li , C. Qu , et al., “A Collagenase Nanogel Backpack Improves CAR‐T Cell Therapy Outcomes in Pancreatic Cancer,” Nature Nanotechnology 20 (2025): 1131–1141, 10.1038/s41565-025-01924-1. 40389641

[24] Y. J. Chae , K.‐G. Lee , D. Oh , S.‐K. Lee , Y. Park , and J. Kim , “Antibody‐Conjugated Nanogel With Two Immune Checkpoint Inhibitors for Enhanced Cancer Immunotherapy,” Advanced Healthcare Materials 13 (2024): 2400235, 10.1002/adhm.202400235. 38569198

[25] T. Nochi , Y. Yuki , H. Takahashi , et al., “Nanogel Antigenic Protein‐delivery System for Adjuvant‐free Intranasal Vaccines,” Nature Materials 9 (2010): 572–578, 10.1038/nmat2784. 20562880

[26] Y. Hashimoto , S. Mukai , Y. Sasaki , and K. Akiyoshi , “Nanogel Tectonics for Tissue Engineering: Protein Delivery Systems With nanogel Chaperones,” Advanced Healthcare Materials 7 (2018): 1800729, 10.1002/adhm.201800729. 30221496

[27] Y. Chao , L. Xu , C. Liang , et al., “Combined Local Immunostimulatory Radioisotope Therapy and Systemic Immune Checkpoint Blockade Imparts Potent Antitumour Responses,” Nature Biomedical Engineering 2 (2018): 611–621, 10.1038/s41551-018-0262-6. 31015634

[28] N. Sahatsapan , T. Ngawhirunpat , T. Rojanarata , P. Opanasopit , and P. Patrojanasophon , “Catechol‐functionalized Alginate Nanoparticles as Mucoadhesive Carriers for Intravesical Chemotherapy,” AAPS PharmSciTech [Electronic Resource] 21 (2020): 212, 10.1208/s12249-020-01752-7. 32737610

[29] Y. J. Xu , K. Wei , P. Zhao , Q. Feng , C. K. K. Choi , and L. Bian , “Preserving the Adhesion of Catechol‐Conjugated Hydrogels by Thiourea–Quinone Coupling,” Biomaterials Science 4 (2016): 1726–1730, 10.1039/C6BM00434B. 27722561

[30] Y.‐N. Wang , Z.‐L. Zeng , J. Lu , et al., “CPT1A‐mediated Fatty Acid Oxidation Promotes Colorectal Cancer Cell Metastasis by Inhibiting Anoikis,” Oncogene 37 (2018): 6025–6040, 10.1038/s41388-018-0384-z. 29995871

[31] M. C. Estañ , et al., “Apoptotic Efficacy of Etomoxir in human Acute Myeloid Leukemia Cells. Cooperation With Arsenic Trioxide and Glycolytic Inhibitors, and Regulation by Oxidative Stress and Protein Kinase Activities,” PLoS ONE 9 (2014): 115250. 10.1371/journal.pone.0115250 PMC4266683 25506699

[32] R. S. O'Connor , L. Guo , S. Ghassemi , et al., “The CPT1a Inhibitor, Etomoxir Induces Severe Oxidative Stress at Commonly Used Concentrations,” Scientific Reports 8 (2018): 6289. 29674640 10.1038/s41598-018-24676-6 PMC5908836

[33] Z. Li , X. Lai , S. Fu , et al., “Immunogenic Cell Death Activates the Tumor Immune Microenvironment to Boost the Immunotherapy Efficiency,” Advanced Science 9 (2022): 2201734, 10.1002/advs.202201734. 35652198 PMC9353475

[34] Y. Engelen , R. Demuynck , J. Ramon , et al., “Immunogenic Cell Death as Interplay Between Physical Anticancer Modalities and Immunotherapy,” Journal of Controlled Release 384 (2025): 113721, 10.1016/j.jconrel.2025.113721. 40368187

[35] D. Li , S. Liu , Y. Ma , S. Liu , Y. Liu , and J. Ding , “Biomaterials That Induce Immunogenic Cell Death,” Small Methods 7 (2023): 2300204. 10.1002/smtd.202300204 37116170

[36] A. Christofides , L. Strauss , A. Yeo , C. Cao , A. Charest , and V. A. Boussiotis , “The Complex Role of Tumor‐infiltrating Macrophages,” Nature Immunology 23 (2022): 1148–1156, 10.1038/s41590-022-01267-2. 35879449 PMC10754321

[37] A. S. Divakaruni , W. Y. Hsieh , L. Minarrieta , et al., “Etomoxir Inhibits Macrophage Polarization by Disrupting CoA Homeostasis,” Cell Metabolism 28 (2018): 490–503.e7, 10.1016/j.cmet.2018.06.001. 30043752 PMC6125190

[38] M. Nomura , J. Liu , I. I. Rovira , et al., “Fatty Acid Oxidation in Macrophage Polarization,” Nature Immunology 17 (2016): 216–217, 10.1038/ni.3366. 26882249 PMC6033271

[39] F. Chen , N. Wang , J. Liao , et al., “Esculetin Rebalances M1/M2 Macrophage Polarization To Treat Sepsis‐Induced Acute Lung Injury Through Regulating Metabolic Reprogramming,” Journal of Cellular and Molecular Medicine 28 (2024): 70178, 10.1111/jcmm.70178. PMC11558263 39535339

[40] D. Namgaladze and B. Brüne , “Fatty Acid Oxidation Is Dispensable for human Macrophage IL‐4‐induced Polarization,” Biochimica et Biophysica Acta (BBA)‐Molecular and Cell Biology of Lipids 1841 (2014): 1329–1335. 24960101 10.1016/j.bbalip.2014.06.007

[41] X. He , T. Deng , J. Li , et al., “A Core‐satellite Micellar System Against Primary Tumors and Their Lymphatic Metastasis Through Modulation of Fatty Acid Metabolism Blockade and Tumor‐associated Macrophages,” Nanoscale 15 (2023): 8320–8336, 10.1039/D2NR04693H. 37083874

[42] A. Covarrubias , V. Byles , and T. Horng , “ROS Sets the Stage for Macrophage Differentiation,” Cell Research 23 (2013): 984–985, 10.1038/cr.2013.88. 23835480 PMC3731567

[43] B. Griess , S. Mir , K. Datta , and M. Teoh‐Fitzgerald , “Scavenging Reactive Oxygen Species Selectively Inhibits M2 Macrophage Polarization and Their Pro‐tumorigenic Function in Part, via Stat3 Suppression,” Free Radical Biology and Medicine 147 (2020): 48–60, 10.1016/j.freeradbiomed.2019.12.018. 31863907 PMC10035558

[44] J. Chen , Y. Zhu , C. Wu , and J. Shi , “Engineering Lactate‐modulating Nanomedicines for Cancer Therapy,” Chemical Society Reviews 52 (2023): 973–1000, 10.1039/D2CS00479H. 36597879

[45] T. Li , D. Xu , Z. Ruan , et al., “Metabolism/Immunity Dual‐Regulation Thermogels Potentiating Immunotherapy of Glioblastoma Through Lactate‐Excretion Inhibition and PD‐1/PD‐L1 Blockade,” Advanced Science 11 (2024): 2310163, 10.1002/advs.202310163. 38460167 PMC11095231

[46] A. Llibre , S. Kucuk , A. Gope , M. Certo , and C. Mauro , “Lactate: A Key Regulator of the Immune Response,” Immunity 58 (2025): 535–554, 10.1016/j.immuni.2025.02.008. 40073846

[47] F. Duan , W. Jin , T. Zhang , Y. Sun , X. Deng , and W. Gao , “Thermo‐pH‐Sensitive Polymer Conjugated Glucose Oxidase for Tumor‐Selective Starvation‐Oxidation‐Immune Therapy,” Advanced Materials 35 (2023): 2209765, 10.1002/adma.202209765. 36773963

[48] S. Lei , J. Zhang , N. T. Blum , et al., “In vivo Three‐dimensional Multispectral Photoacoustic Imaging of Dual Enzyme‐driven Cyclic Cascade Reaction for Tumor Catalytic Therapy,” Nature Communications 13 (2022): 1298, 10.1038/s41467-022-29082-1. PMC8917194 35277519

[49] L. Fu , C. Qi , Y. Hu , J. Lin , and P. Huang , “Glucose Oxidase‐Instructed Multimodal Synergistic Cancer Therapy,” Advanced Materials 31 (2019): 1808325, 10.1002/adma.201808325. 30907460

[50] W. Zhao , J. Hu , and W. Gao , “Glucose Oxidase–Polymer Nanogels for Synergistic Cancer‐Starving and Oxidation Therapy,” ACS Applied Materials & Interfaces 9 (2017): 23528–23535, 10.1021/acsami.7b06814. 28650613

[51] S. Li , Q. Wang , Z. Jia , et al., “Recent Advances in Glucose Oxidase‐based Nanocarriers for Tumor Targeting Therapy,” Heliyon 9 (2023): 20407. 10.1016/j.heliyon.2023.e20407 PMC10539972 37780773

[52] X. Qin , C. Yu , J. Wei , et al., “Rational Design of Nanocarriers for Intracellular Protein Delivery,” Advanced Materials 31 (2019): 1902791, 10.1002/adma.201902791. 31496027

[53] D. Bi , R. Zhou , N. Cai , et al., “Alginate Enhances Toll‐Like Receptor 4‐mediated Phagocytosis by Murine RAW264.7 Macrophages,” International Journal of Biological Macromolecules 105 (2017): 1446–1454, 10.1016/j.ijbiomac.2017.07.129. 28739412

[54] V. Rahmani and H. Sheardown , “Protein‐alginate Complexes as pH‐/Ion‐sensitive Carriers of Proteins,” International Journal of Pharmaceutics 535 (2018): 452–461, 10.1016/j.ijpharm.2017.11.039. 29170114

[55] E. J. Delgado‐Pujol , G. Martínez , D. Casado‐Jurado , et al., “Hydrogels and Nanogels: Pioneering the Future of Advanced Drug Delivery Systems,” Pharmaceutics 17 (2025): 215, 10.3390/pharmaceutics17020215. 40006582 PMC11859140

[56] S. Bazban‐Shotorbani , E. Dashtimoghadam , A. Karkhaneh , M. M. Hasani‐Sadrabadi , and K. I. Jacob , “Microfluidic Directed Synthesis of Alginate Nanogels With Tunable Pore Size for Efficient Protein Delivery,” Langmuir 32 (2016): 4996–5003, 10.1021/acs.langmuir.5b04645. 26938744

[57] L. Zhang , J. Sun , W. Huang , S. Zhang , X. Deng , and W. Gao , “Hypoxia‐Triggered Bioreduction of Poly( N ‐oxide)–Drug Conjugates Enhances Tumor Penetration and Antitumor Efficacy,” Journal of the American Chemical Society 145 (2023): 1707–1713, 10.1021/jacs.2c10188. 36601987

[58] X. Liu and W. Gao , “Precision Conjugation: An Emerging Tool for Generating Protein–Polymer Conjugates,” Angewandte Chemie International Edition 60 (2021): 11024–11035, 10.1002/anie.202003708. 32437042

[59] Z. Cao , C. Liu , J. Wen , and Y. Lu , “Innovative Formulation Platform: Paving the Way for Superior Protein Therapeutics With Enhanced Efficacy and Broadened Applications,” Advanced Materials 36 (2024): 2403116, 10.1002/adma.202403116. PMC11571700 38819929

[60] L. Yan , K. Xu , C. Liu , et al., “Polymer‐Formulated Nerve Growth Factor Shows Effective Therapeutic Efficacy for Cerebral Microinfarcts,” Advanced Materials 37 (2025): 2412843, 10.1002/adma.202412843. 39601176

[61] R. Wang , C. Zhang , Y. Cao , et al., “Blockade of Dual Immune Checkpoint Inhibitory Signals With a CD47/PD‐L1 Bispecific Antibody for Cancer Treatment,” Theranostics 13 (2023): 148–160, 10.7150/thno.79367. 36593962 PMC9800731

[62] C.‐Y. Wu , et al., “Glucose Restriction Shapes Pre‐metastatic Innate Immune Landscapes in the Lung Through Exosomal TRAIL,” Cell 188 (2025): P5701–5716.e19, 10.1016/j.cell.2025.06.027. 40669460

[63] E. E. W. Cohen , D. Soulières , C. Le Tourneau , et al., “Pembrolizumab versus Methotrexate, Docetaxel, or Cetuximab for Recurrent or Metastatic Head‐and‐neck Squamous Cell Carcinoma (KEYNOTE‐040): A Randomised, Open‐label, Phase 3 Study,” Lancet 393 (2019): 156–167, 10.1016/S0140-6736(18)31999-8. 30509740

[64] J. M. Zaretsky , A. Garcia‐Diaz , D. S. Shin , et al., “Mutations Associated With Acquired Resistance to PD‐1 Blockade in Melanoma,” New England Journal of Medicine 375 (2016): 819–829, 10.1056/NEJMoa1604958. 27433843 PMC5007206

