# Supplementary material for: Spatiotemporal Targeting Randle Cycle and Immune Checkpoint for Potent Antitumor Therapy
Source: Adv Sci (Weinh). 2026 Jun 15:e76109. Online ahead of print. doi: 10.1002/advs.76109 (PMC13337094; doi:10.1002/advs.76109)
Supplement: Supplementary file 1 — Supporting File: advs76109‐sup‐0001‐SuppMat.docx. [file ADVS-9999-e76109-s001.docx]

**Spatiotemporal Targeting Randle Cycle and Immune Checkpoint for Potent Antitumor Therapy**

*Yuan Gao^ab^, Zijian Gong^ab^, Yixuan Fu^ab^, Jianan Zheng^ab^, Xiao Sang^ab^, Binglin Chen^ab^, Qinzhi Su^ab^, Weiping Gao^b^, Fei Duan^e^, Jinqi Wei^d^*, Xuliang Deng^abc^*, Xinyu Liu^ab^**

^a^Central Laboratory, NMPA Key Laboratory for Dental Materials, National Engineering Research Center of Oral Biomaterials and Digital Medical Devices, Beijing Laboratory of Biomedical Materials, Beijing Key Laboratory of Biomaterials for Oral Disease, National Center for Stomatology, National Clinical Research Center for Oral Diseases, Peking University School and Hospital of Stomatology, Beijing, 100081, P. R. China

^b^Institute of Advanced Clinical Medicine, Peking University, Beijing, 100191, P. R. China

^c^Department of Geriatric Dentistry, Peking University School and Hospital of Stomatology, Beijing, 100081, P. R. China

^d^First Clinical Division, Peking University School and Hospital of Stomatology, Beijing, 100034, P. R. China

^e^Department of Nanomedicine, Translational Medicine Research Center, & Shanghai Key Laboratory of Nautical Medicine and Translation of Drugs and Medical Devices, Naval Medical University, Shanghai 200433, P. R. China.

E-mail: liuxinyu@hsc.pku.edu.cn; kqdengxuliang@bjmu.edu.cn; weijinqipkuss@bjmu.edu.cn

Funding: Supported by the National Key R&D Program of China (2022YFC2405900, 2022YFC2405903); NSFC/China (82202327, 82402194); Beijing Natural Science Foundation (L242126); and Clinical Medicine Plus X-Young Scholars Project, Peking University, the Fundamental Research Funds for the Central Universities (PKU2024LCXQ018).


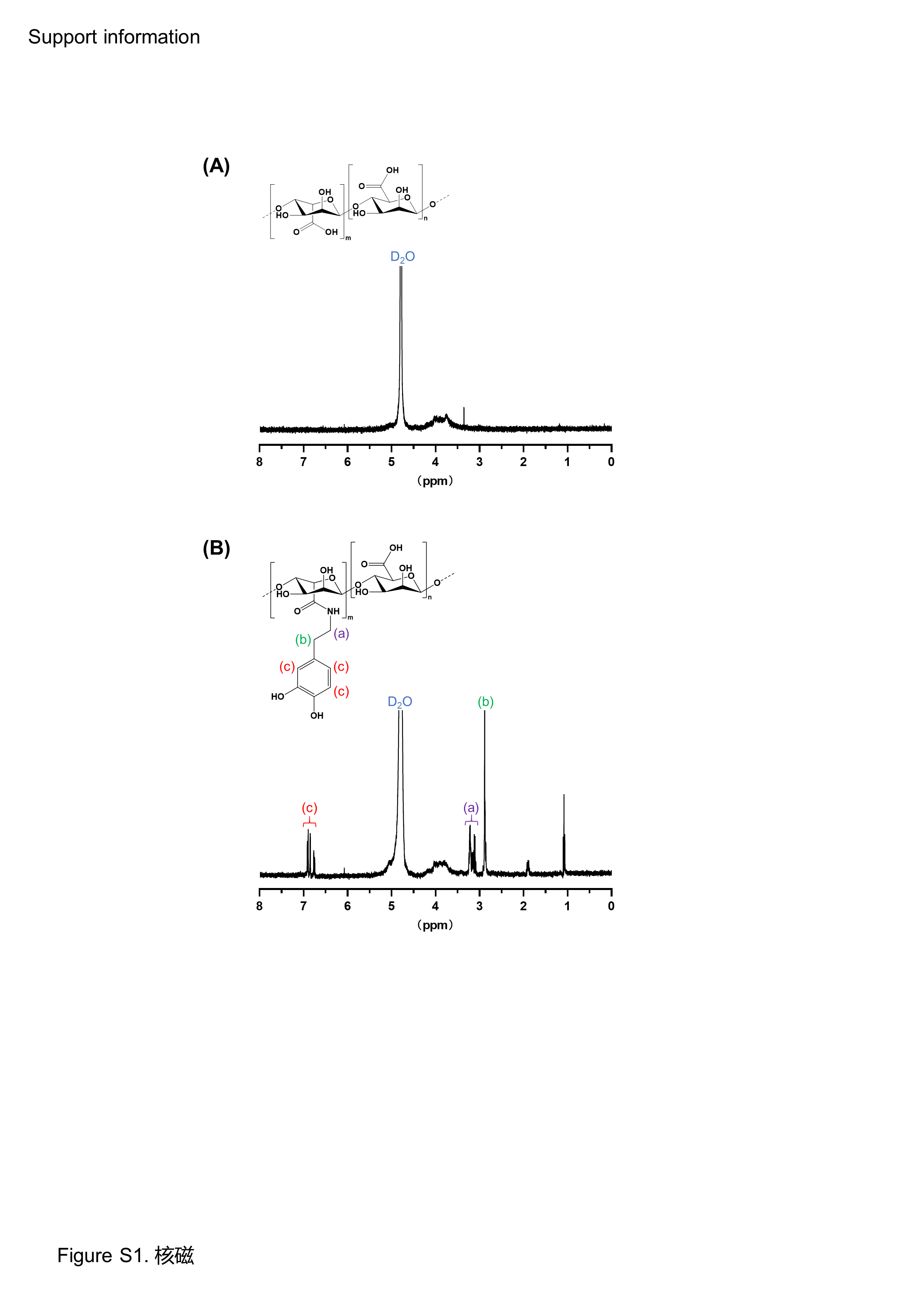


**Figure S1.** ^1^H-NMR spectra of (A) alginate and (B) Cat-Alg.


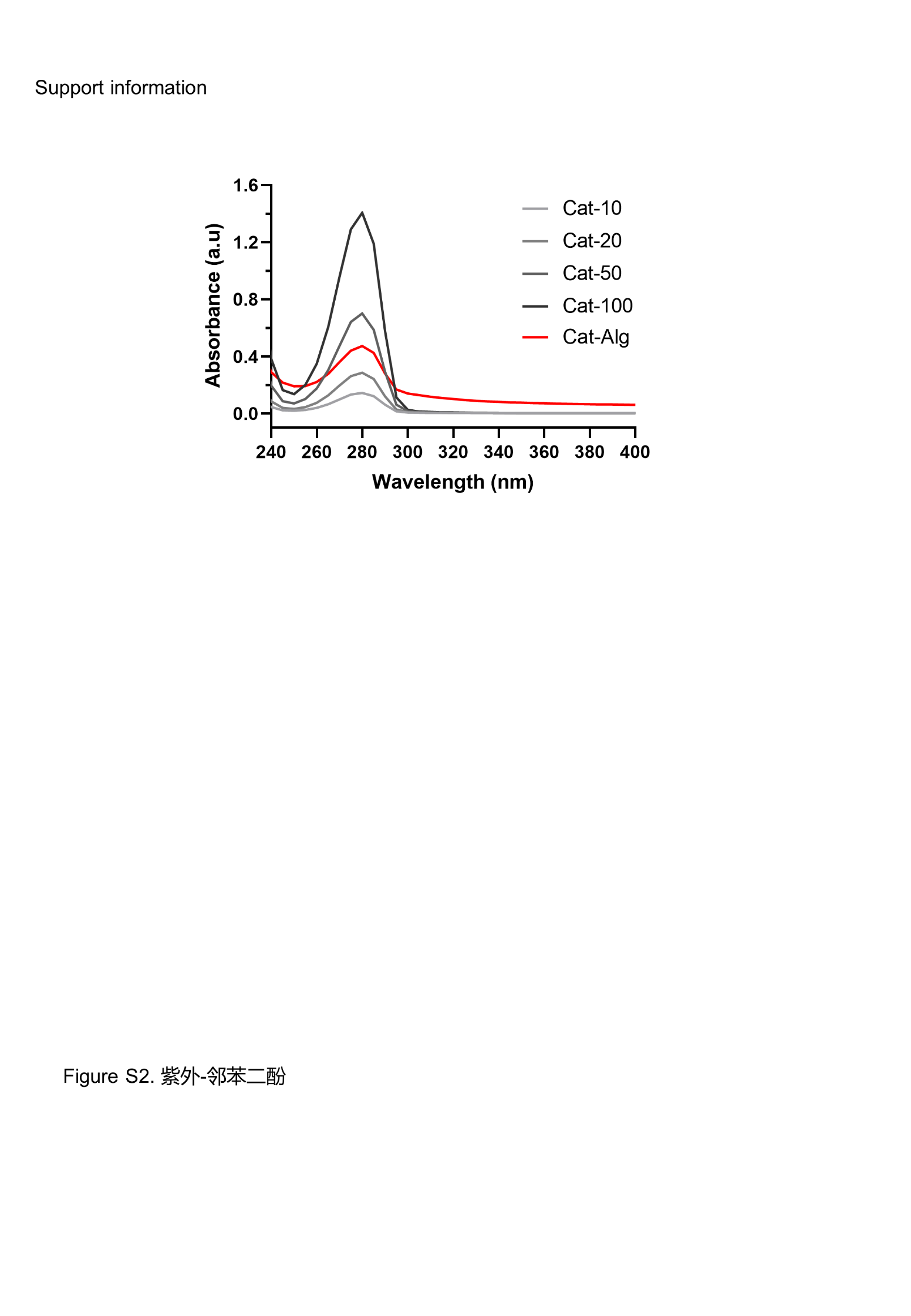


**Figure S2.** The characteristic absorption peak of catechol group was at 280 nm in the UV absorption spectra of dopamine hydrochloride (10, 20, 50, 100 μg mL^-1^) and Cat-Alg.


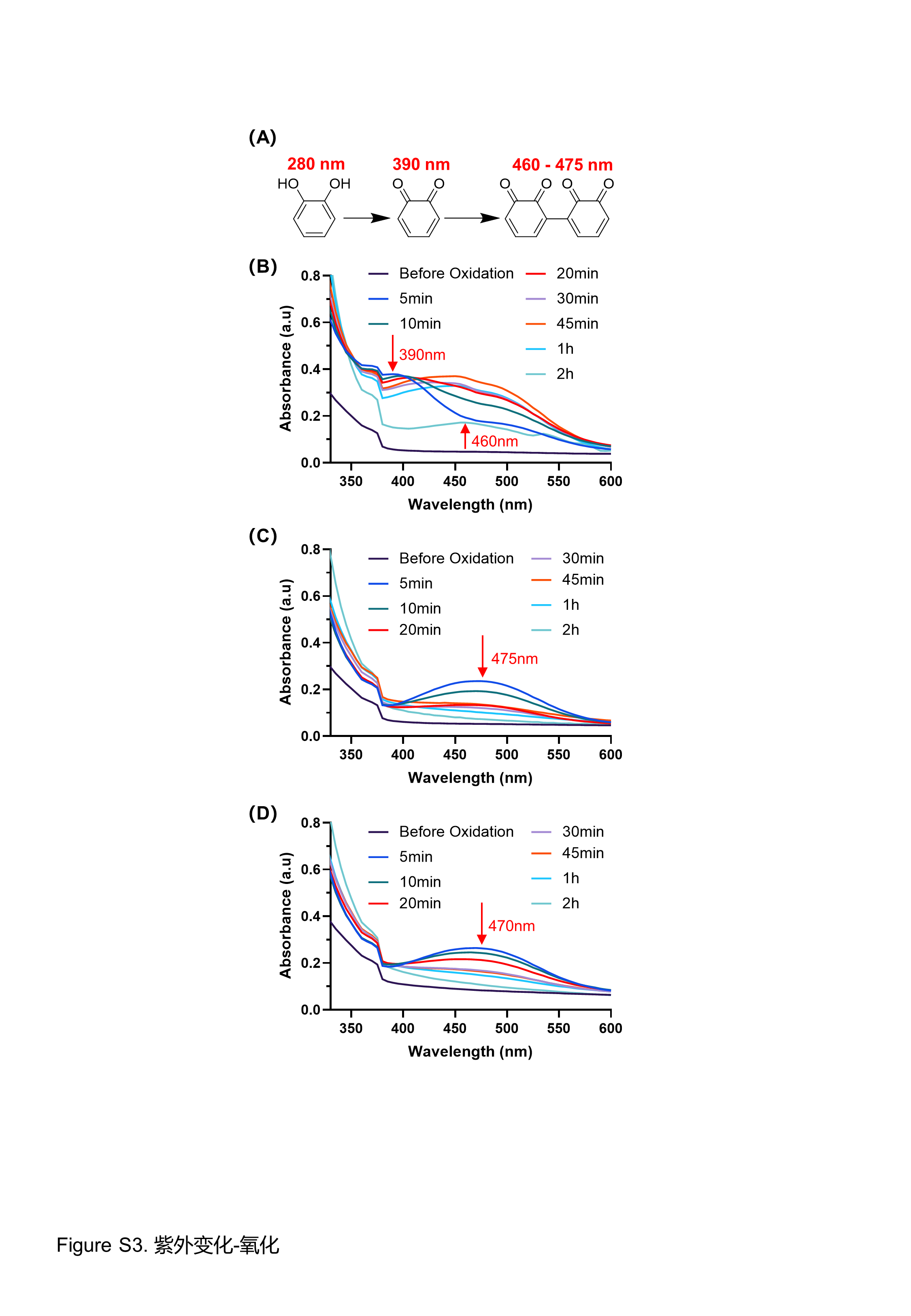


**Figure S3.** (A) The oxidation of the catechol group to the O-diquinone structure. (B) Changes in UV-vis spectra were measured at different times after addition of NaIO_4_ to Dopa solution (100 μg mL^-1^). (C) Changes in UV-vis spectra were measured at different times after addition of NaIO_4_ to Cat-Alg solution (100 μg mL^-1^).


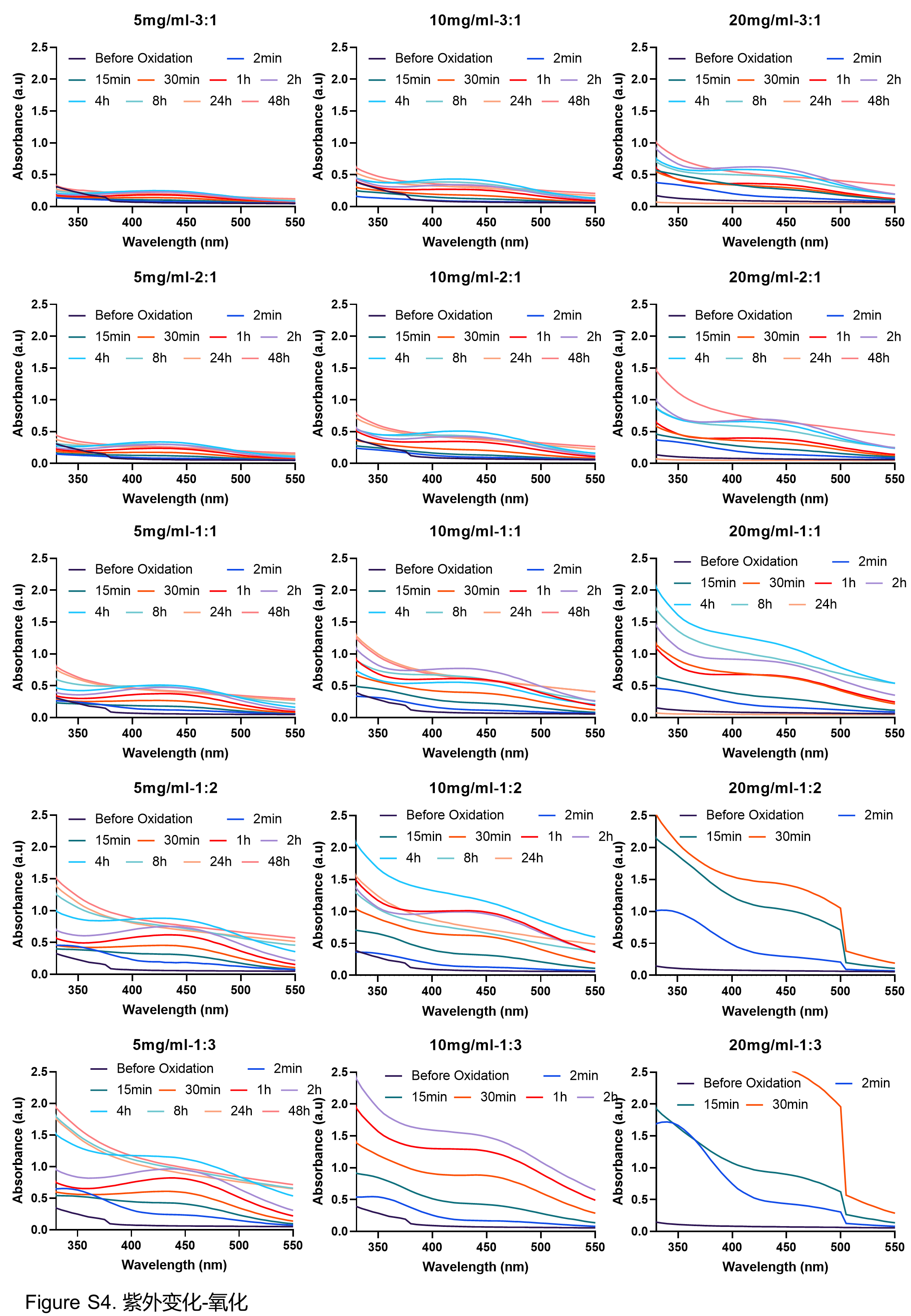


**Figure S4.** The effects of different concentrations of Cat-Alg (Concentration = 5, 10, 20 mg mL^-1^) and different Cat/ IO_4_^-^ molar ratios (3:1, 2:1, 1:1, 1:2, 1:3) on the oxidation process of Cat-Alg were analyzed by UV spectra.


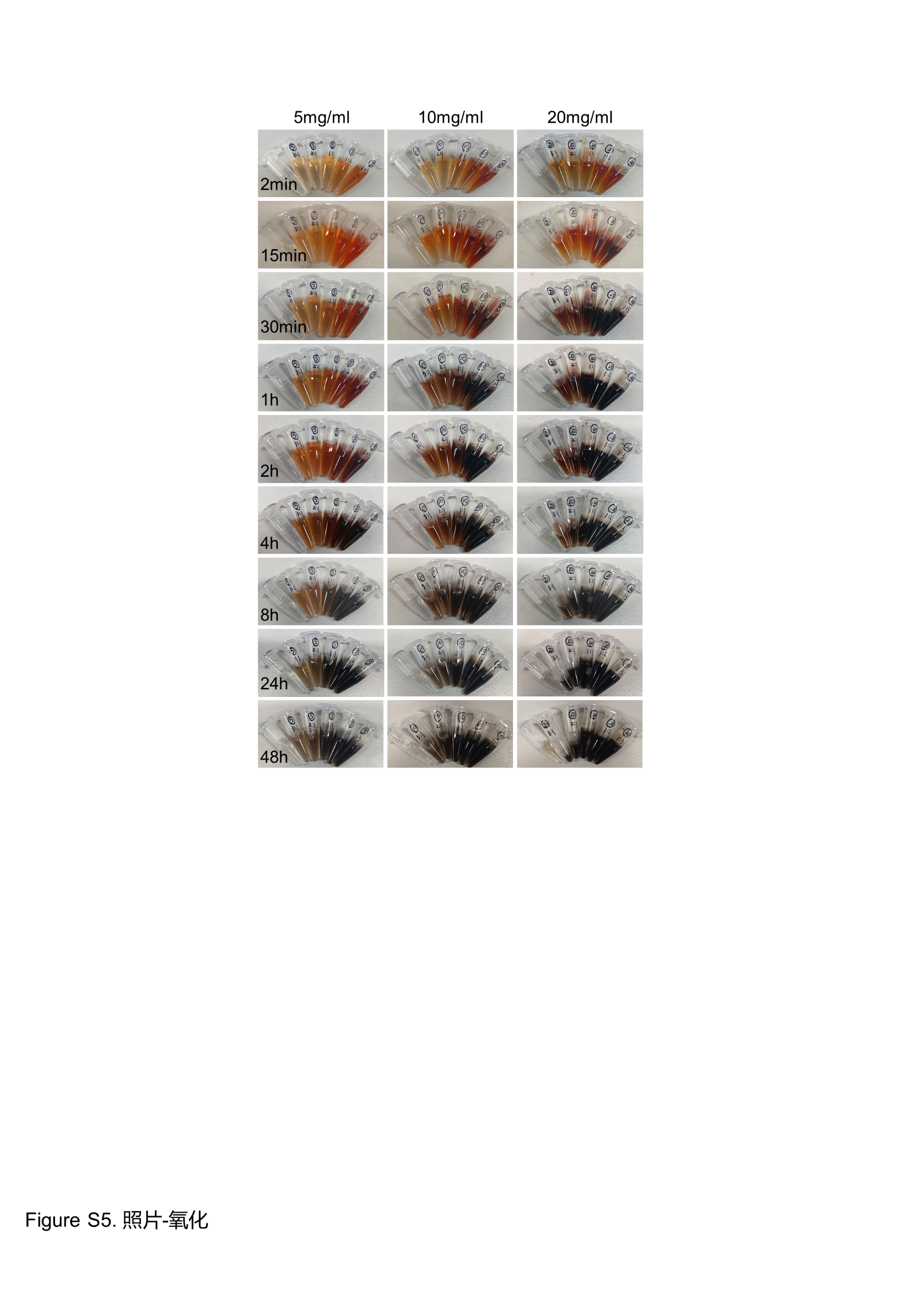


**Figure S5.** Photographs of the oxidation process of Cat-Alg at different concentrations (5, 10, 20 mg mL^-1^) and different Cat/IO_4_^-^ molar ratios (3:1, 2:1, 1:1, 1:2, 1:3).


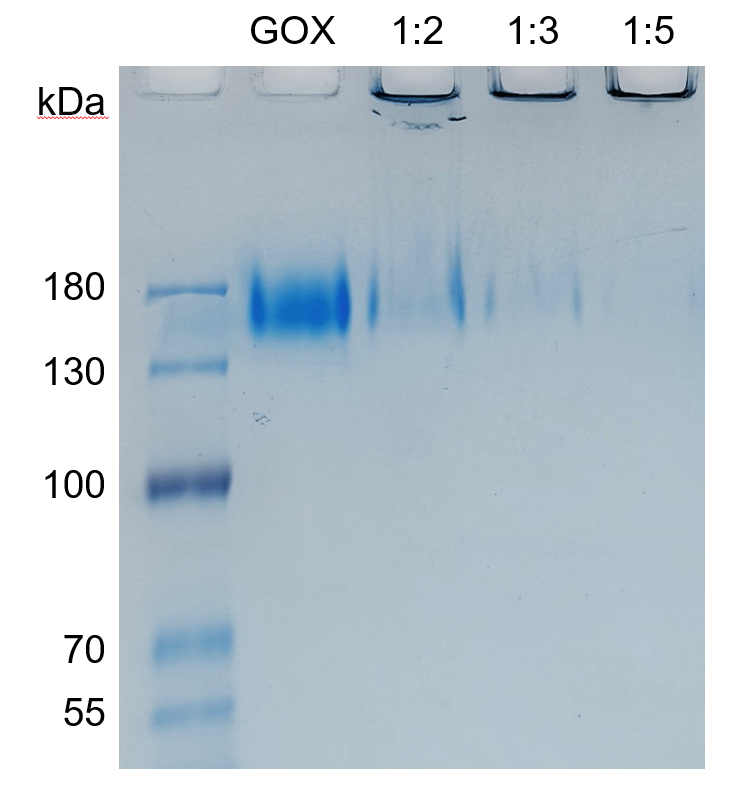


**Figure S6.** SDS PAGE of GOX and GOX-NG (mass ratio of GOX/Cat-Alg = 1:2, 1:3 and 1:5).


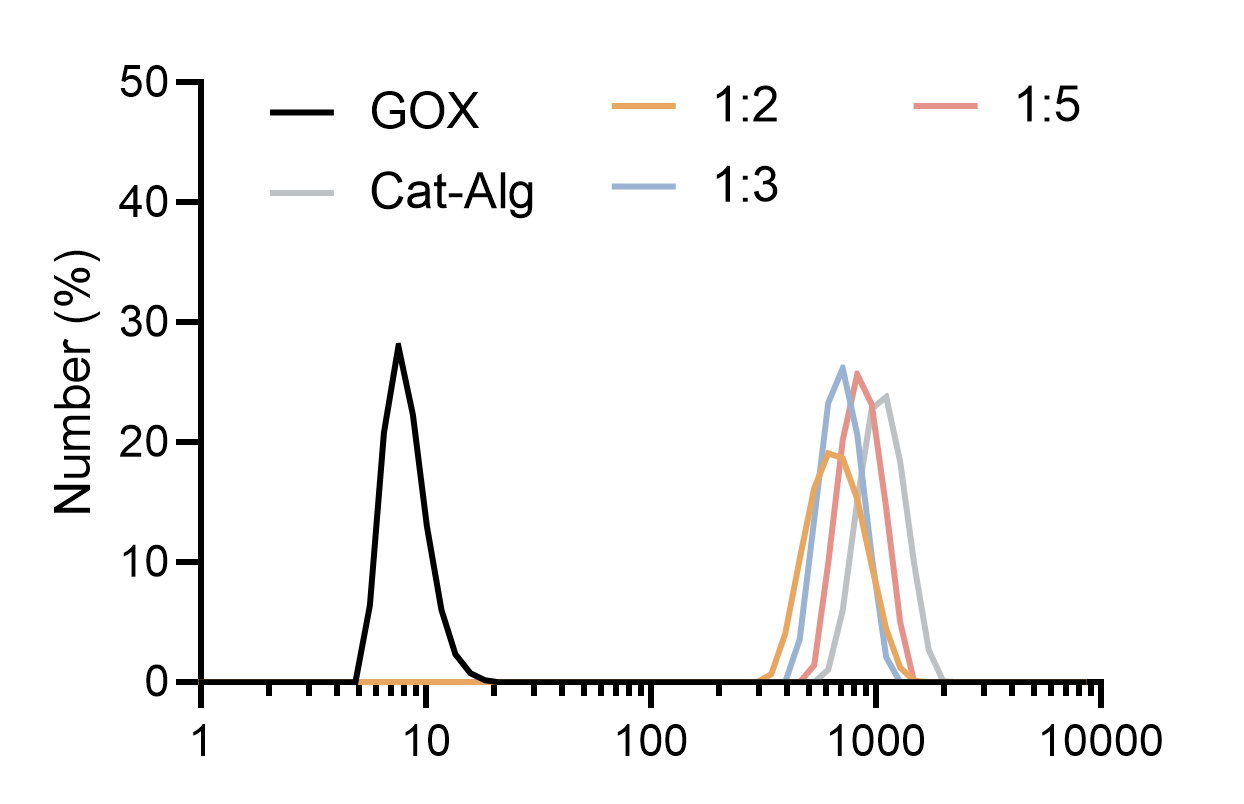


**Figure S7.** Hydrated particle size curves of GOX, Cat-Alg, and GOX-NG (mass ratio of GOX/Cat-Alg = 1:2, 1:3 and 1:5).


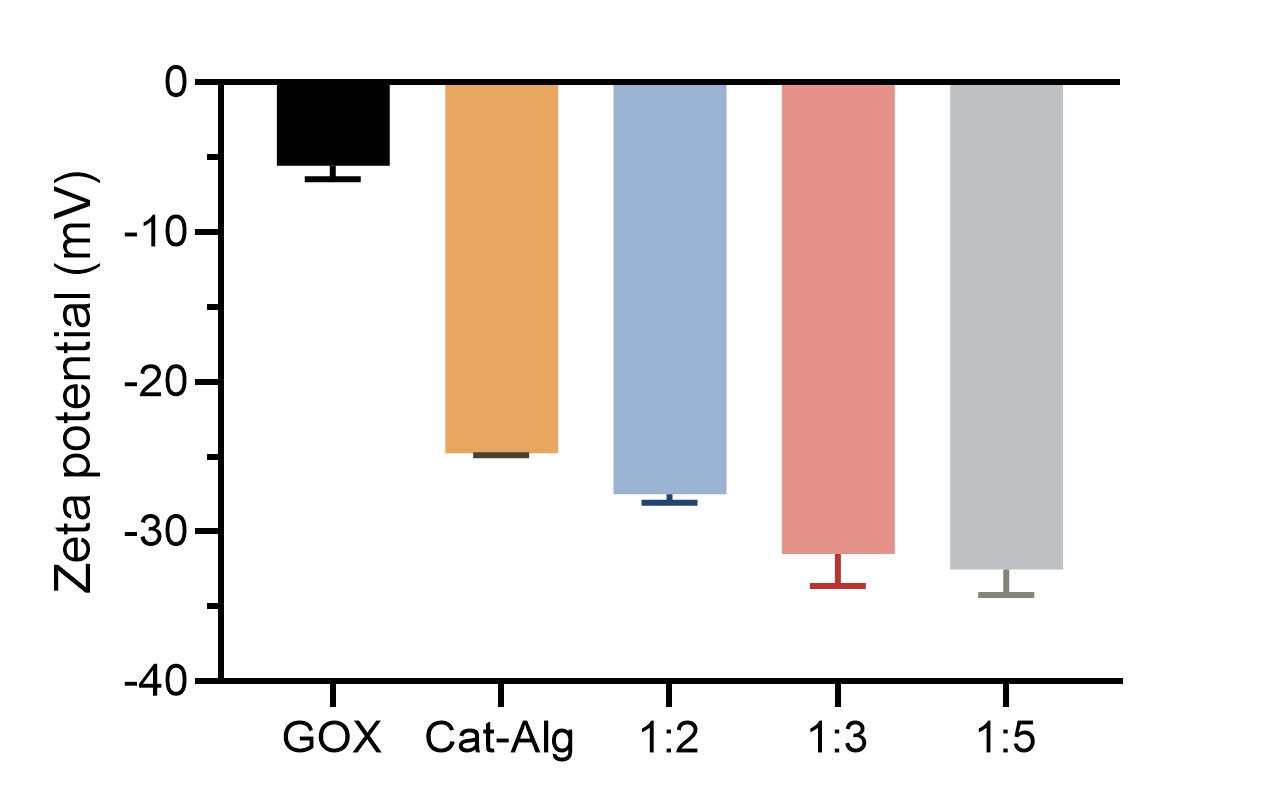


**Figure S8.** Zeta potentials of GOX, Cat-Alg, and GOX-NG (mass ratio of GOX/Cat-Alg = 1:2, 1:3 and 1:5). Data represent the means ± SEM, n = 3.


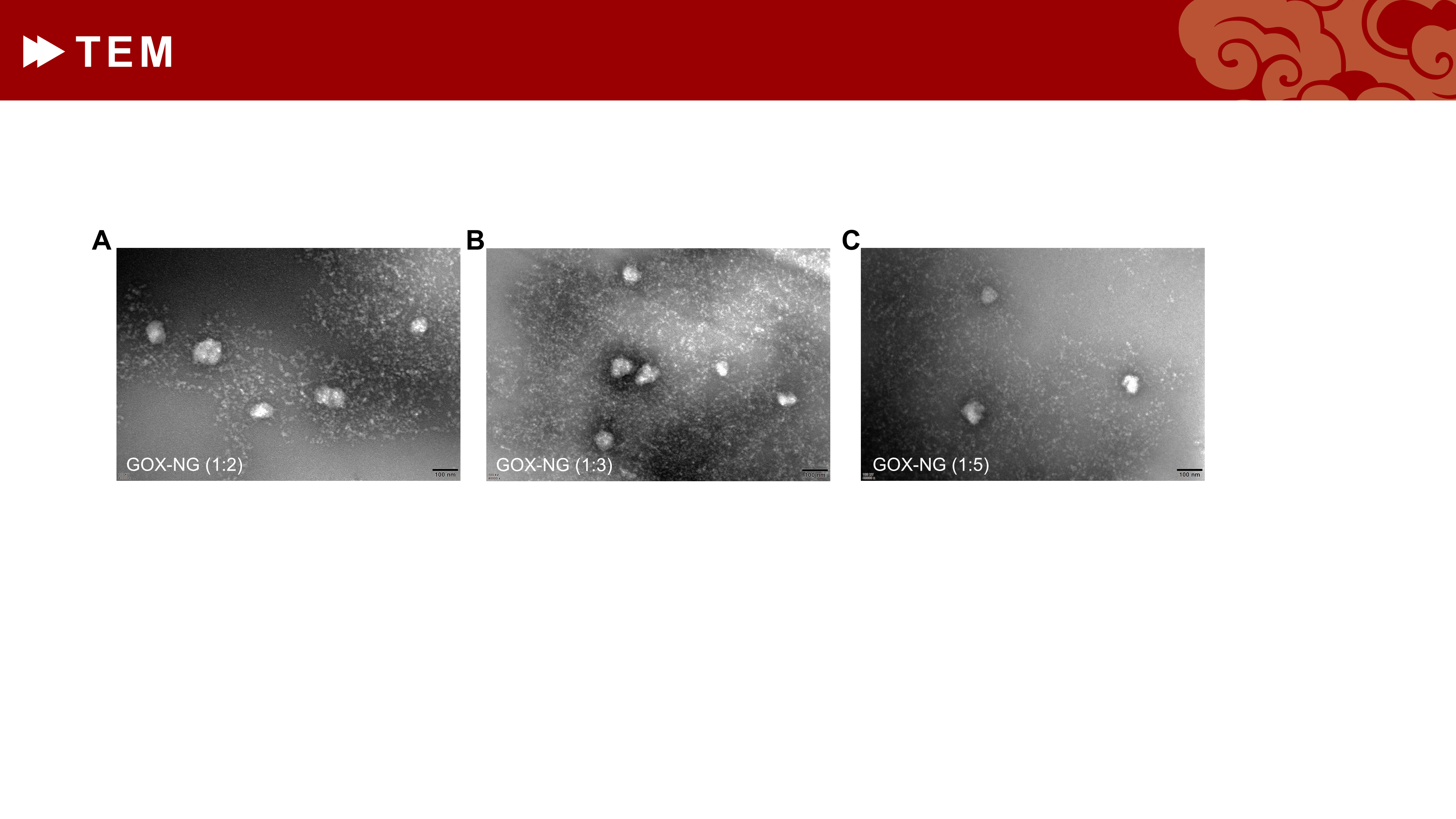


**Figure S9.** TEM images of GOX-NG (mass ratio of GOX/Cat-Alg = 1:2, 1:3 and 1:5). Scale bar, 100 nm.


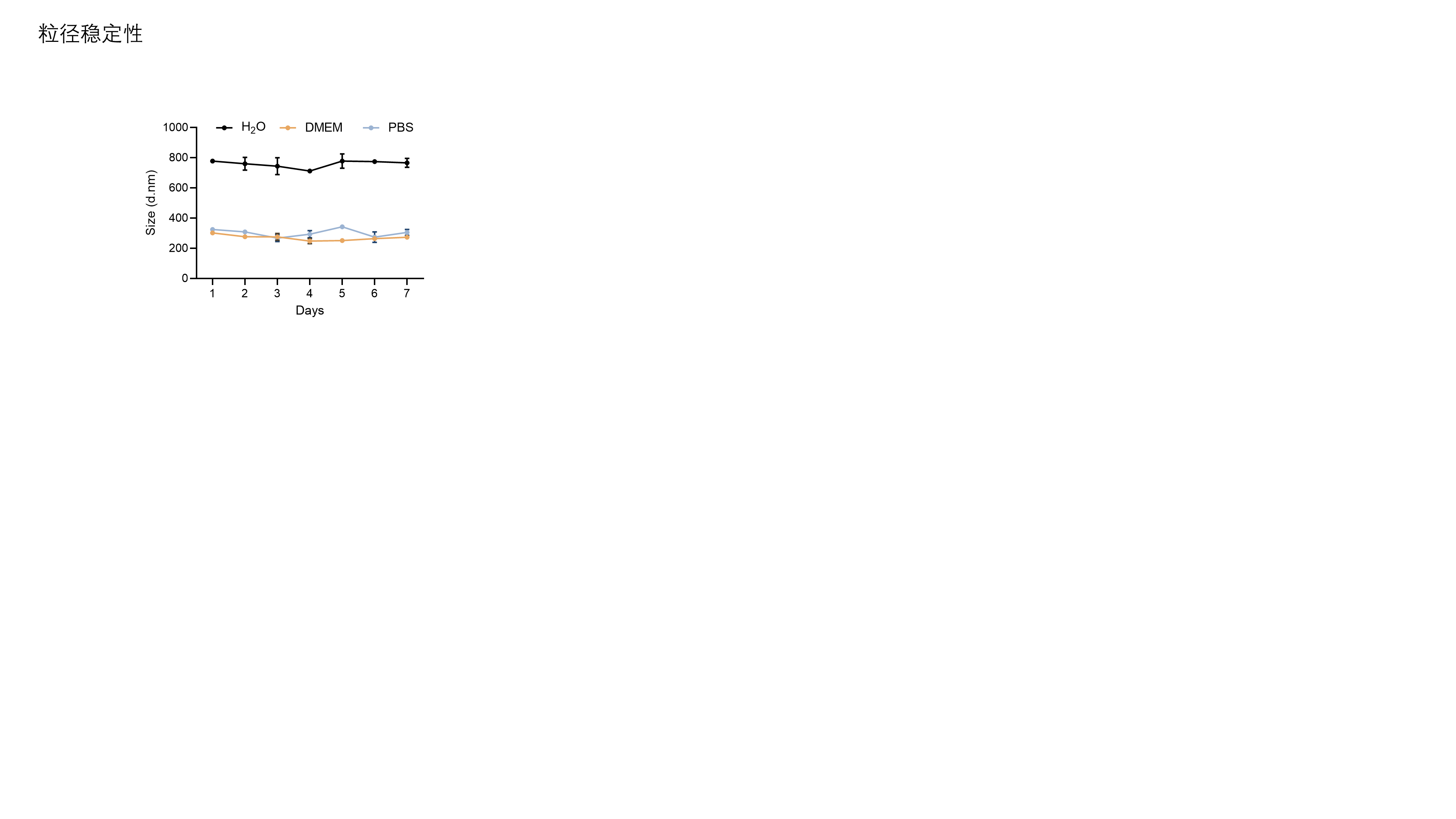


**Figure S10.** Particle size stability of GOX-NG in H₂O, DMEM, and PBS over a one-week period at 37°C.


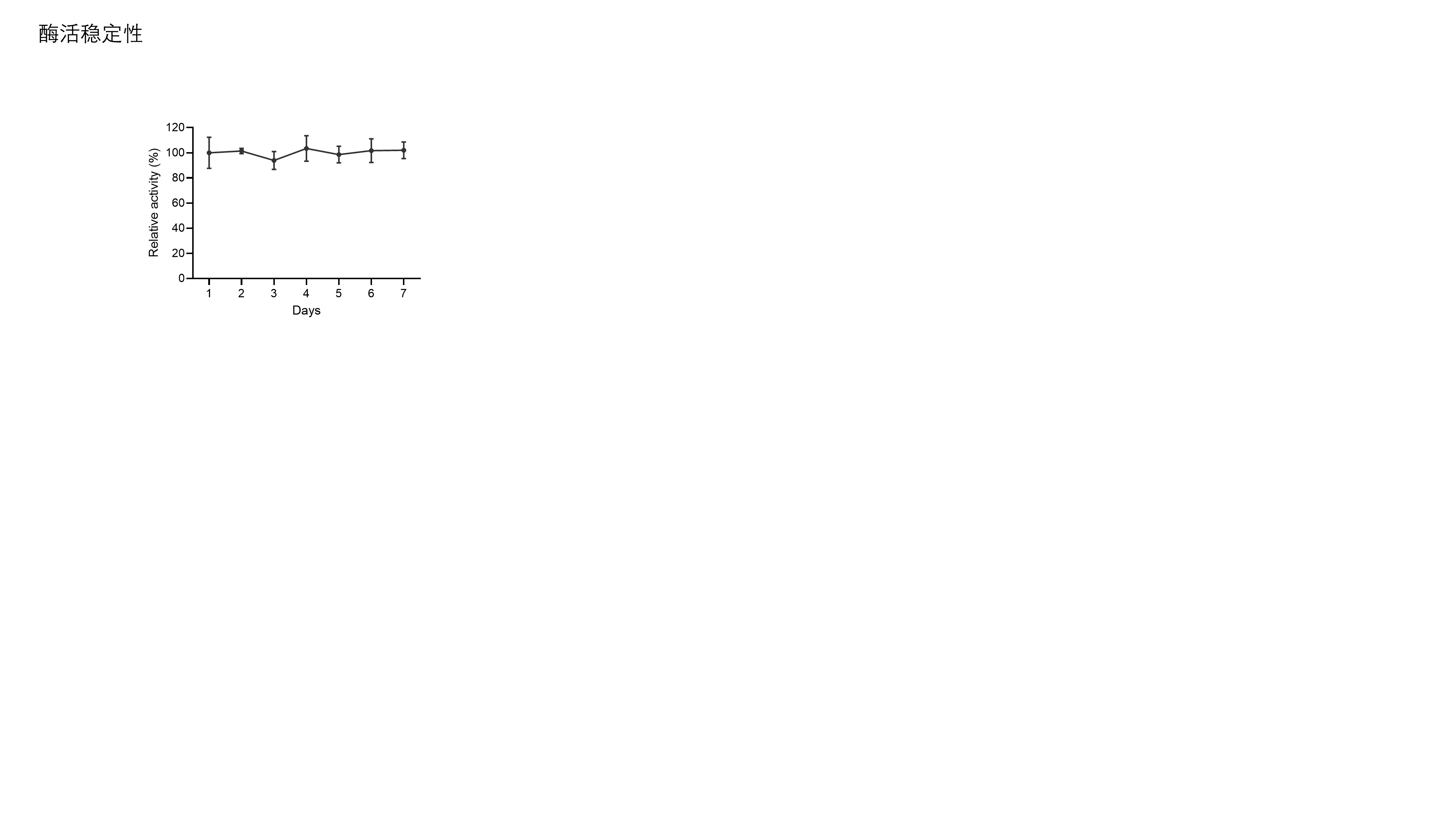


**Figure S11.** Enzyme activity stability of GOX-NG in PBS over a one-week period at 37°C.


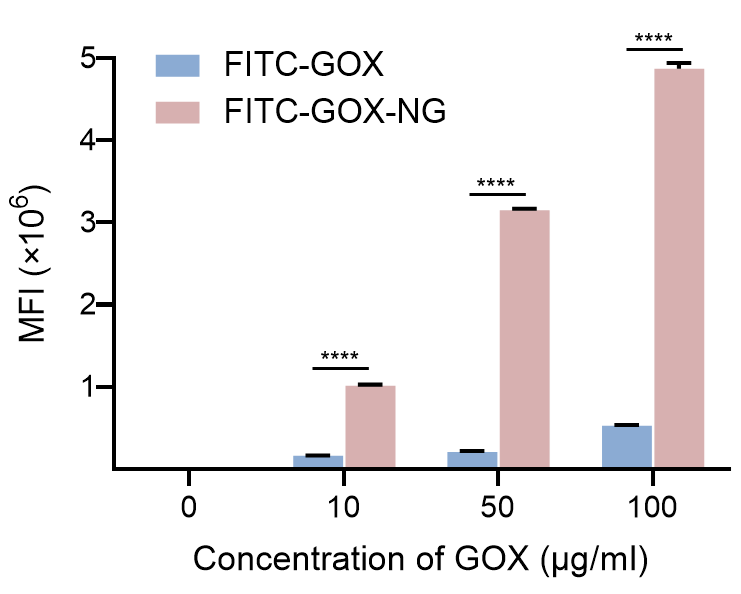


**Figure S12.** After co-incubation of FITC-GOX or FITC-GOX-NG with SCC7 cells for 2 h, the mean fluorescence intensity (MFI) of the cells was quantified by flow cytometry. Data represent the means ± SEM, n = 3.


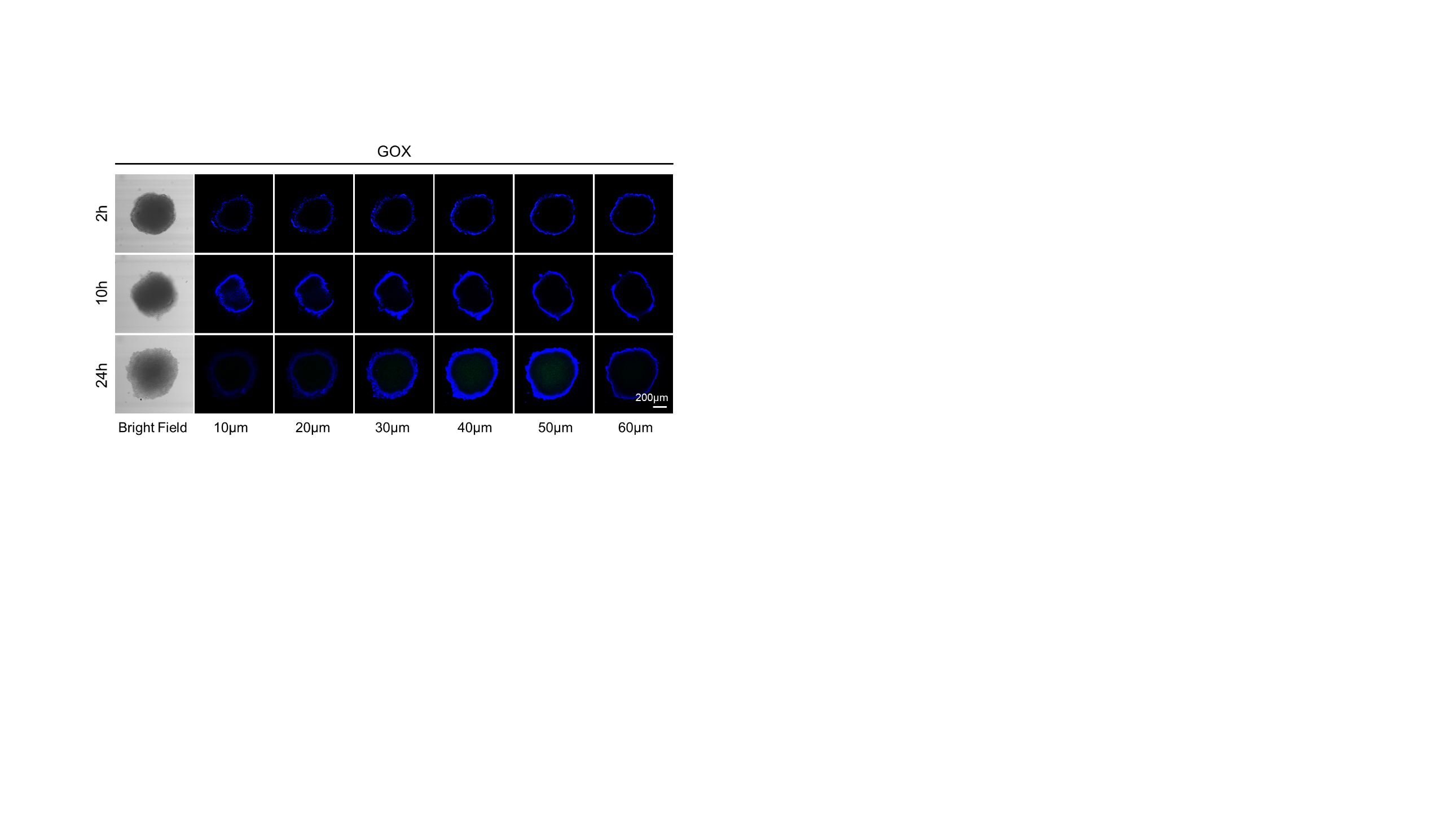


**Figure S13.** Fluorescence images of tumor spheres at different depths taken by CLSM after co-incubation of FITC-GOX with SCC7 tumor spheres for 2, 10, and 24 hours. Scale bar, 200 μm.


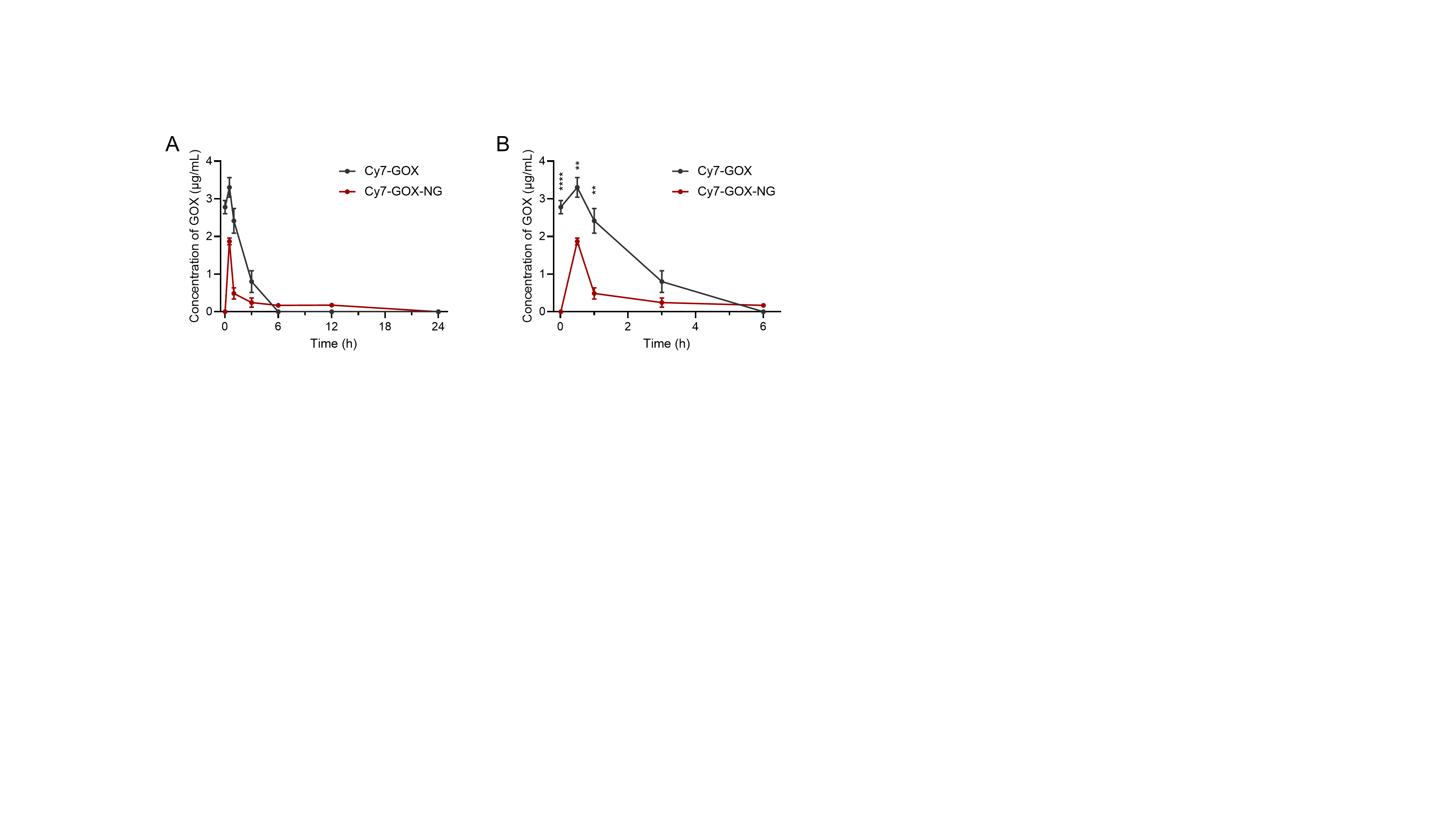


**Figure S14.** (A) Cy7-GOX or Cy7-GOX-NG was injected intratumorally, and serum GOX concentrations were measured at various time points over the next 24 hours. (B) Enlarged graph showing data from 0 to 6 hours.


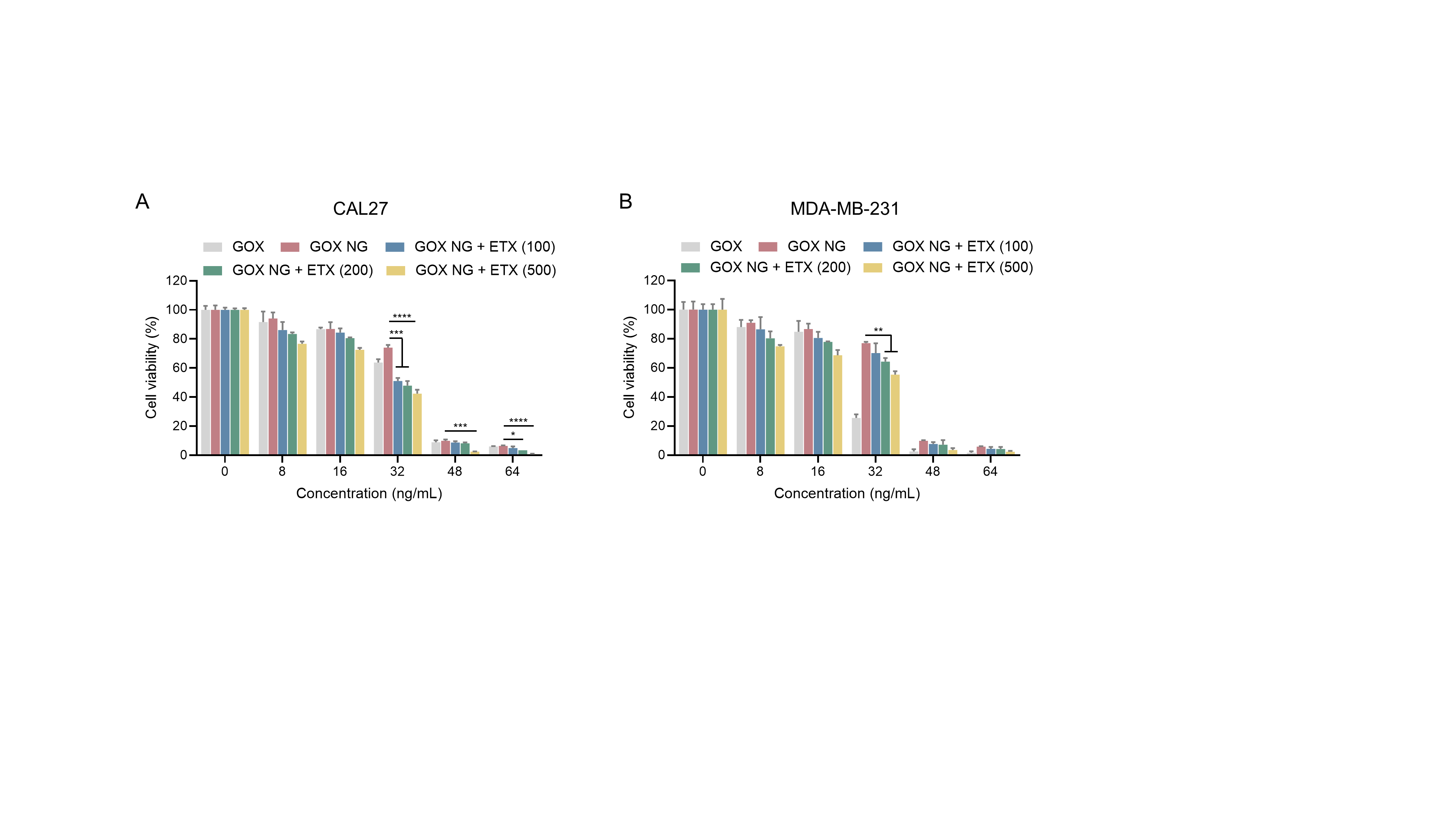


**Figure S15.** (A) The toxic effect of GOX-NG + ETX on CAL27 cells determined by CCK8 assay (n = 3). (B) The toxic effect of GOX-NG + ETX on MDA-MB-231 cells determined by CCK8 assay (n = 3).


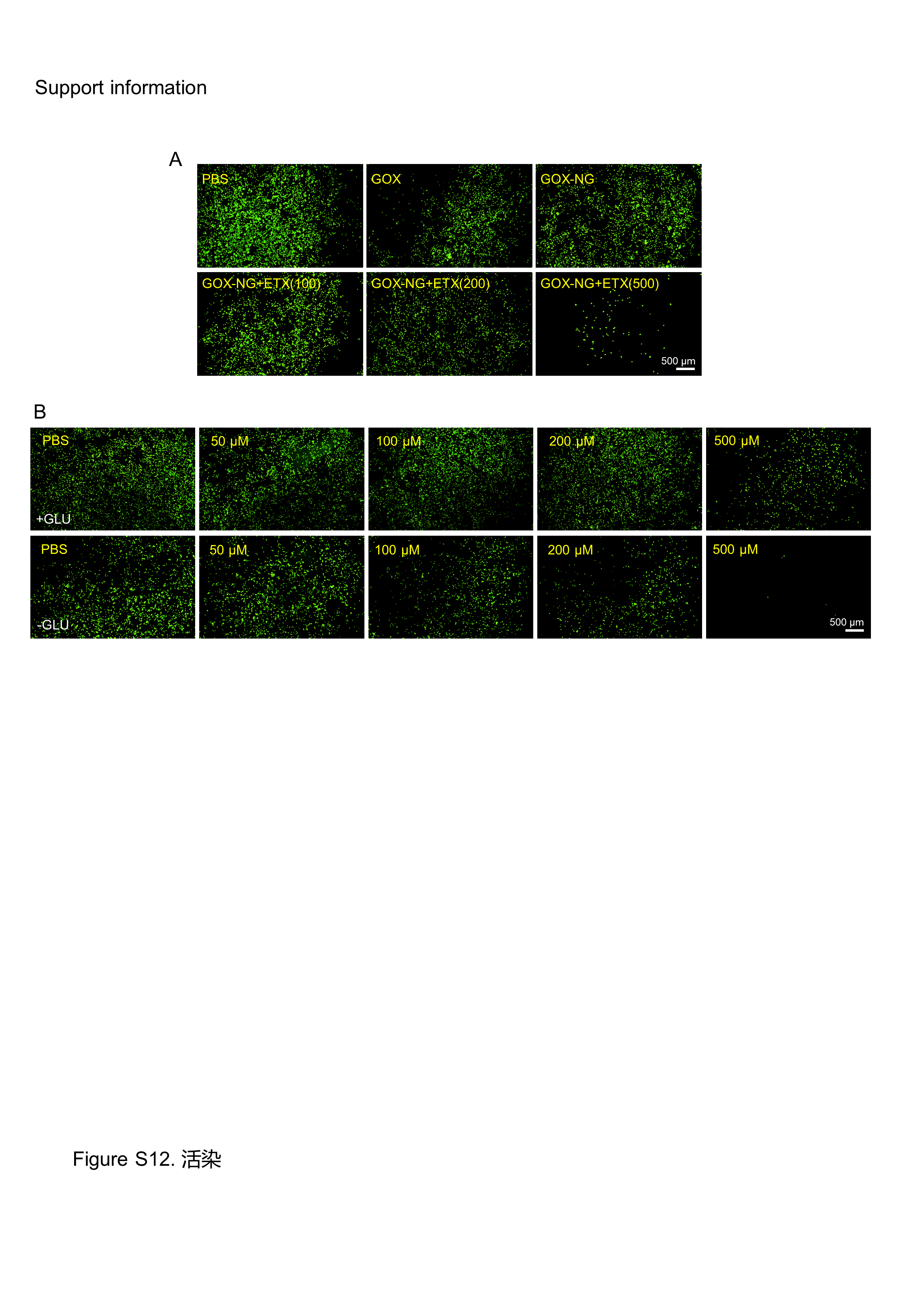


**Figure S16.** After SCC7 cells were incubated with different drug regimens for 24 hours, the living cells were stained (green fluorescence represented the living cells), and fluorescence images were obtained through a fluorescence microscope. Scale bar, 500 μm.


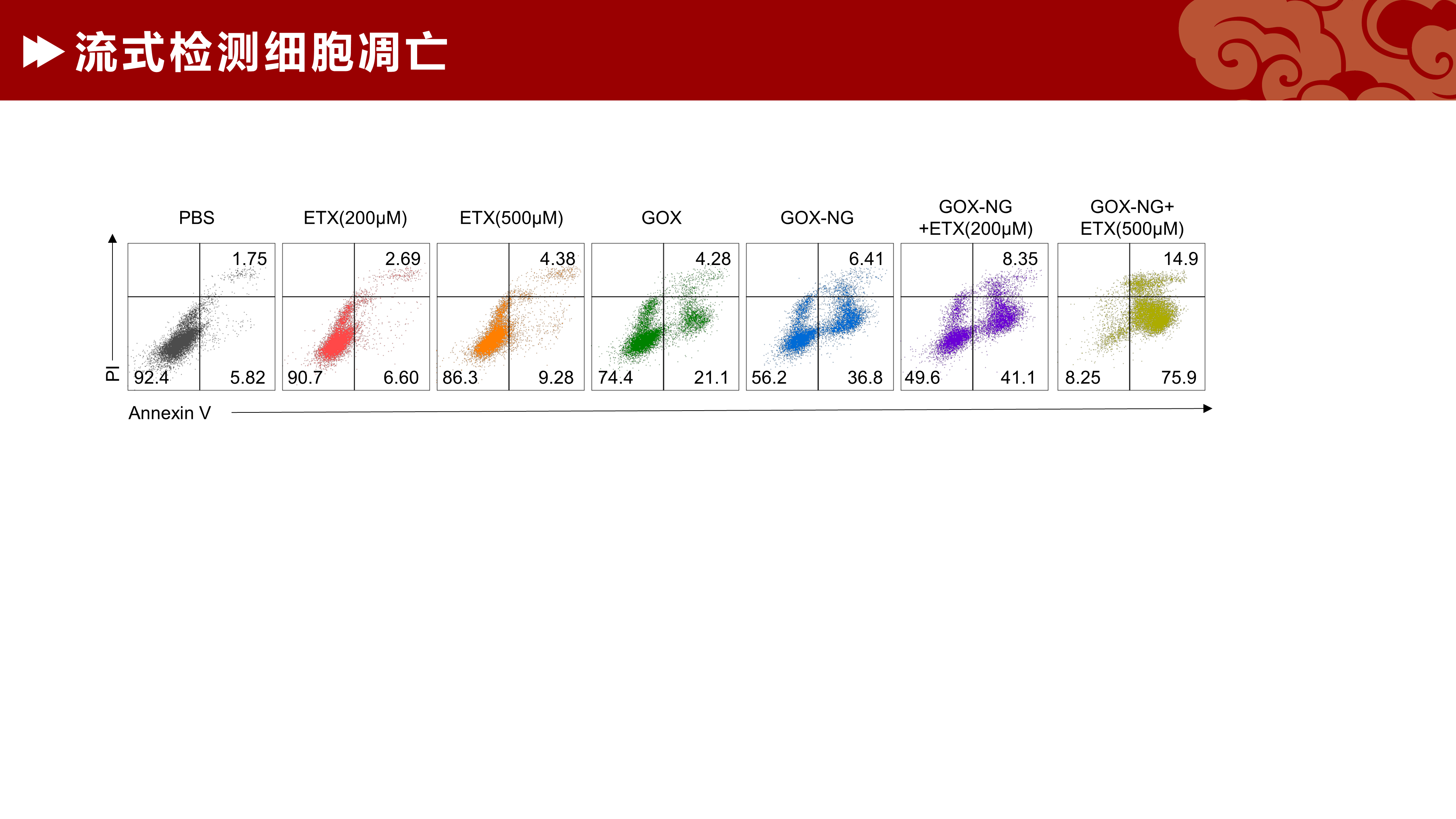


**Figure S17.** After SCC7 cells were co-incubated with drugs of different regimens for 24 hours, apoptosis was detected by flow cytometry.


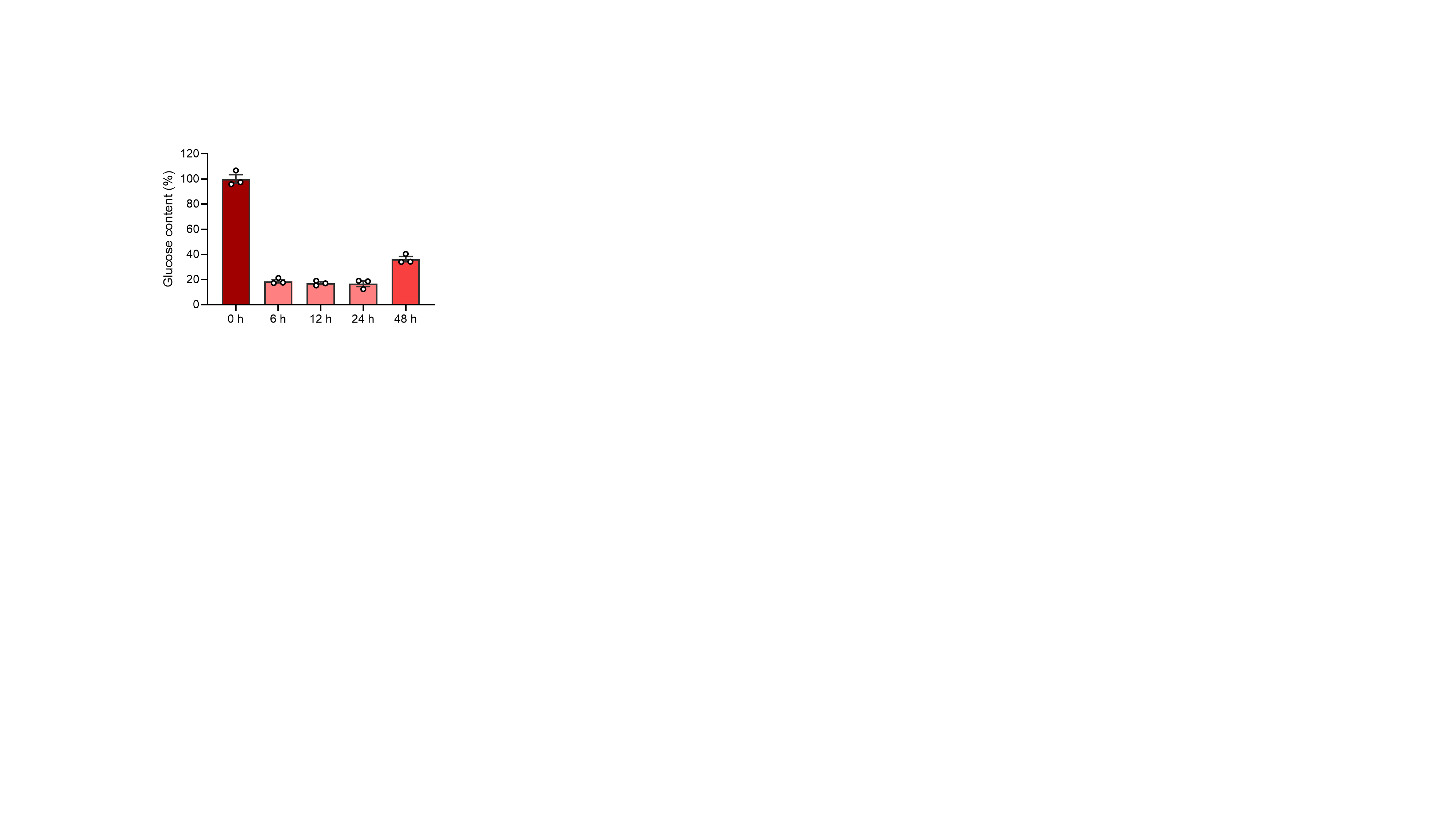


**Figure S18.** Intratumoral glucose levels in tumor-bearing mice after intratumoral injection of GOX-NG.


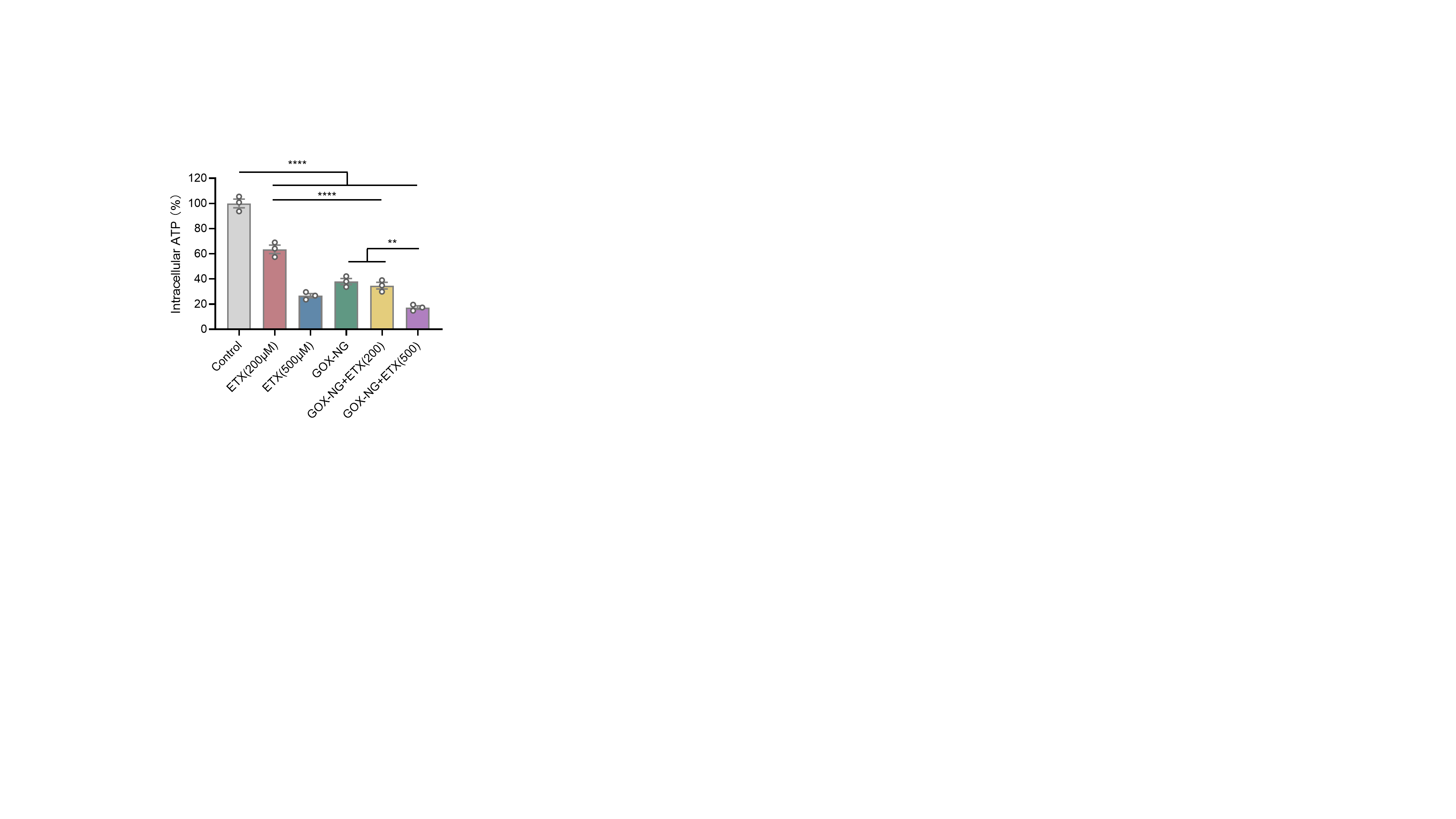


**Figure S19.** Intracellular ATP content of 4T1 cells after incubation with different regimens for 24 h (n = 3).


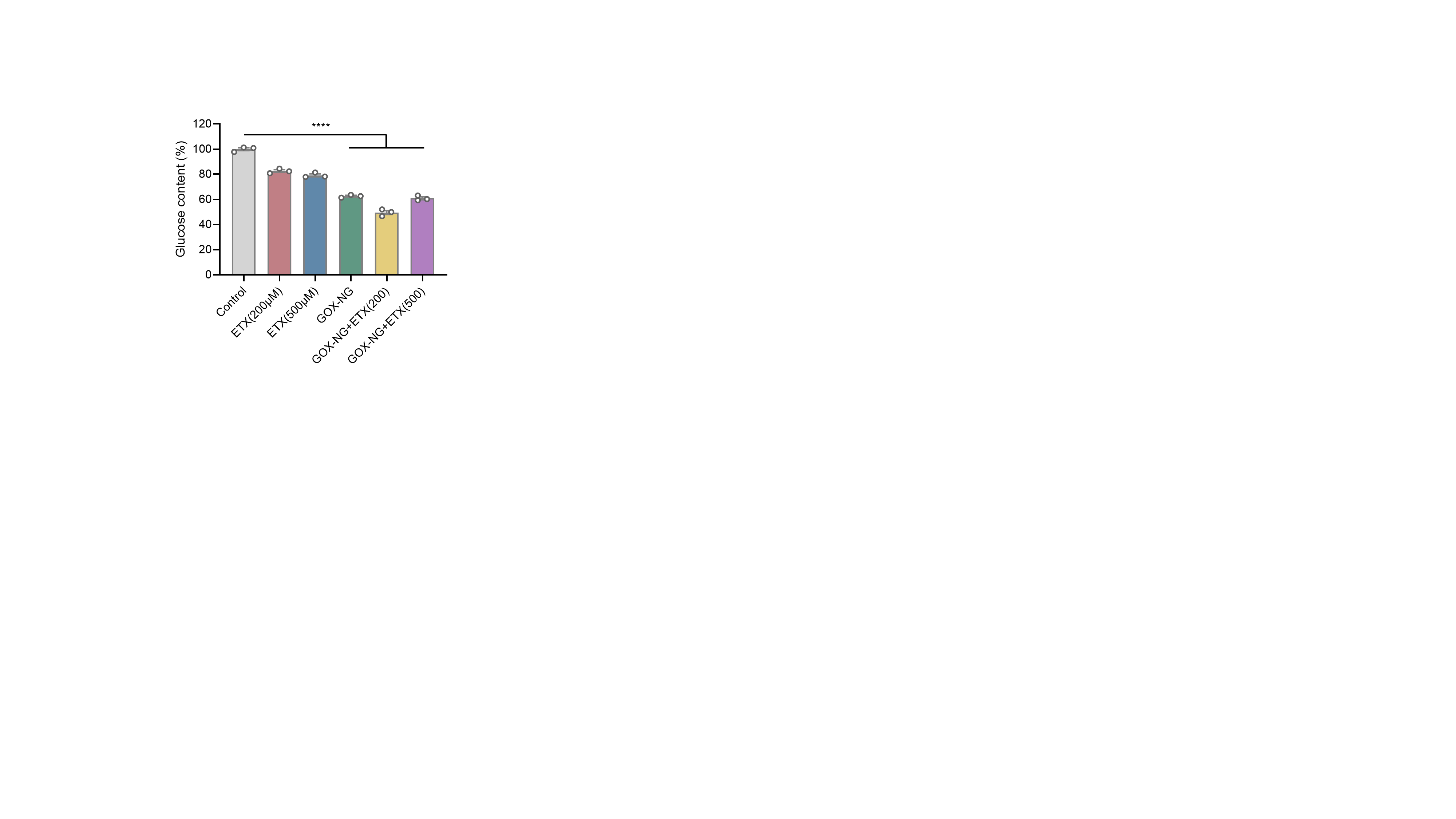


**Figure S20.** Glucose content in the supernatant of 4T1 cells after 24 hours of co-incubation with different drug regimens (n = 3).


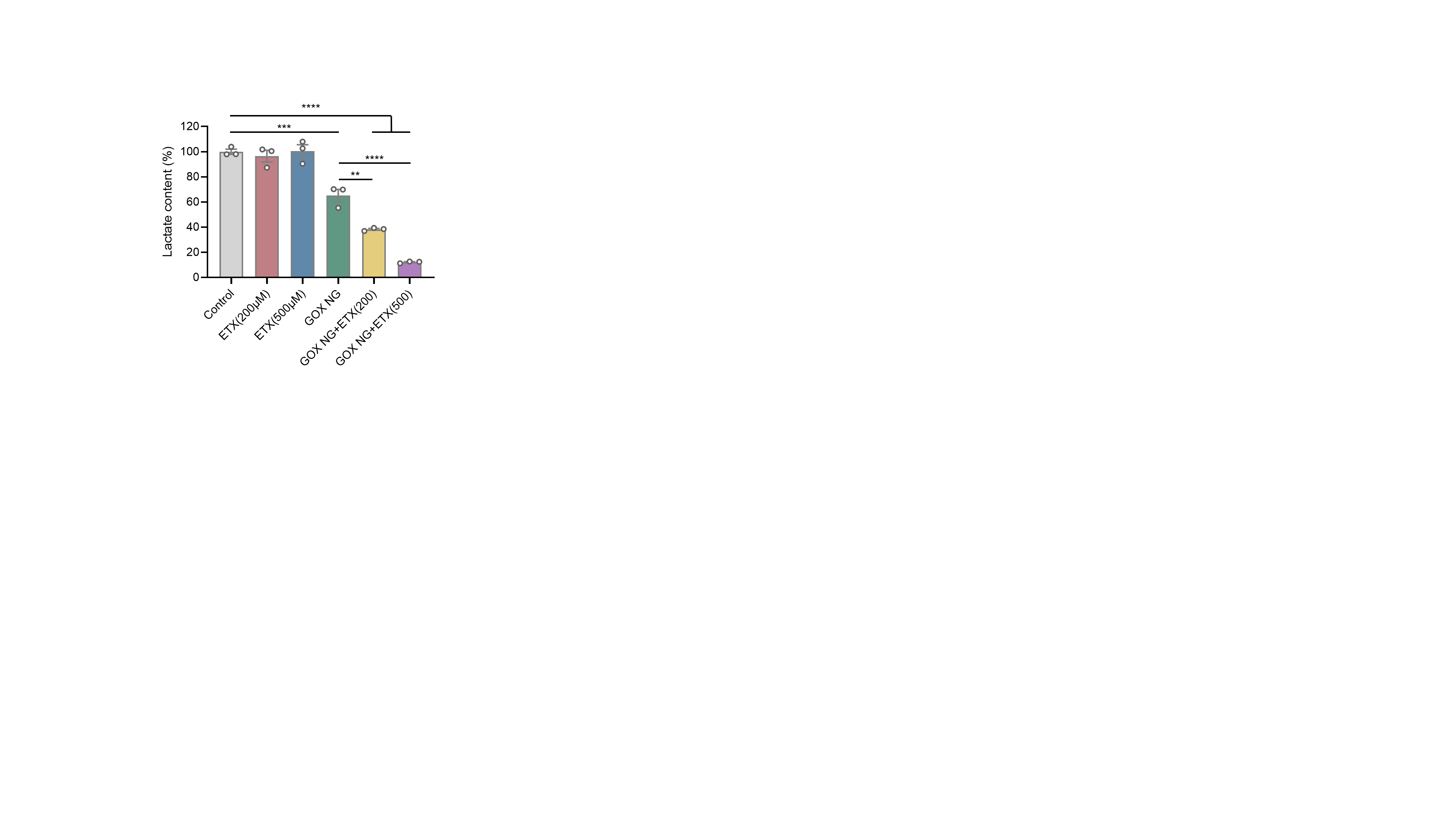


**Figure S21.** Lactate content in the supernatant of 4T1 cells after 24 hours of co-incubation with different drug regimens (n = 3).


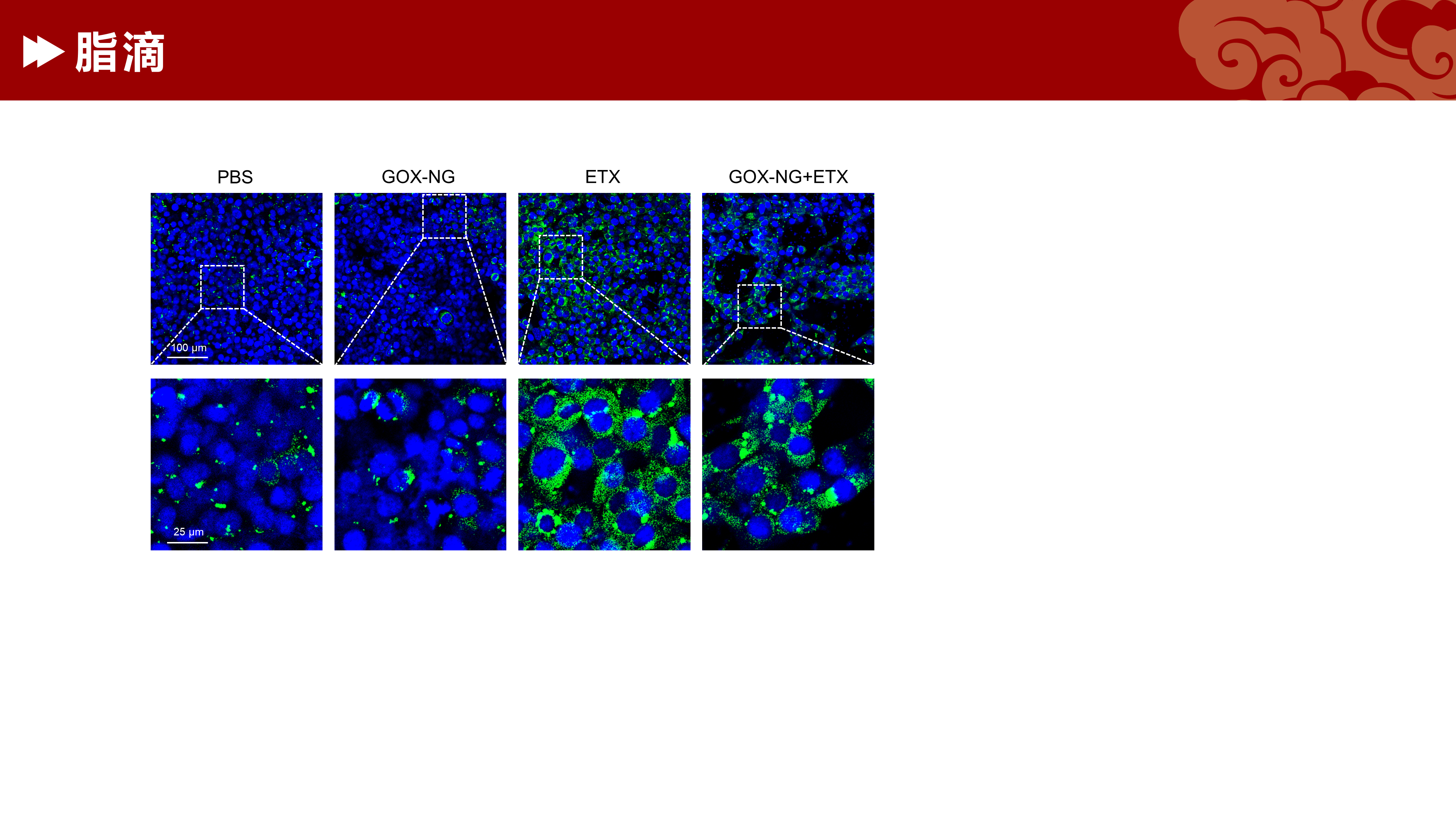


**Figure S22.** After SCC7 cells were incubated with different drug regimens for 24 hours, the intracellular lipid droplets (green fluorescence represented lipid droplets) were captured by CLSM. Scale bar, 20 μm or 10 μm.


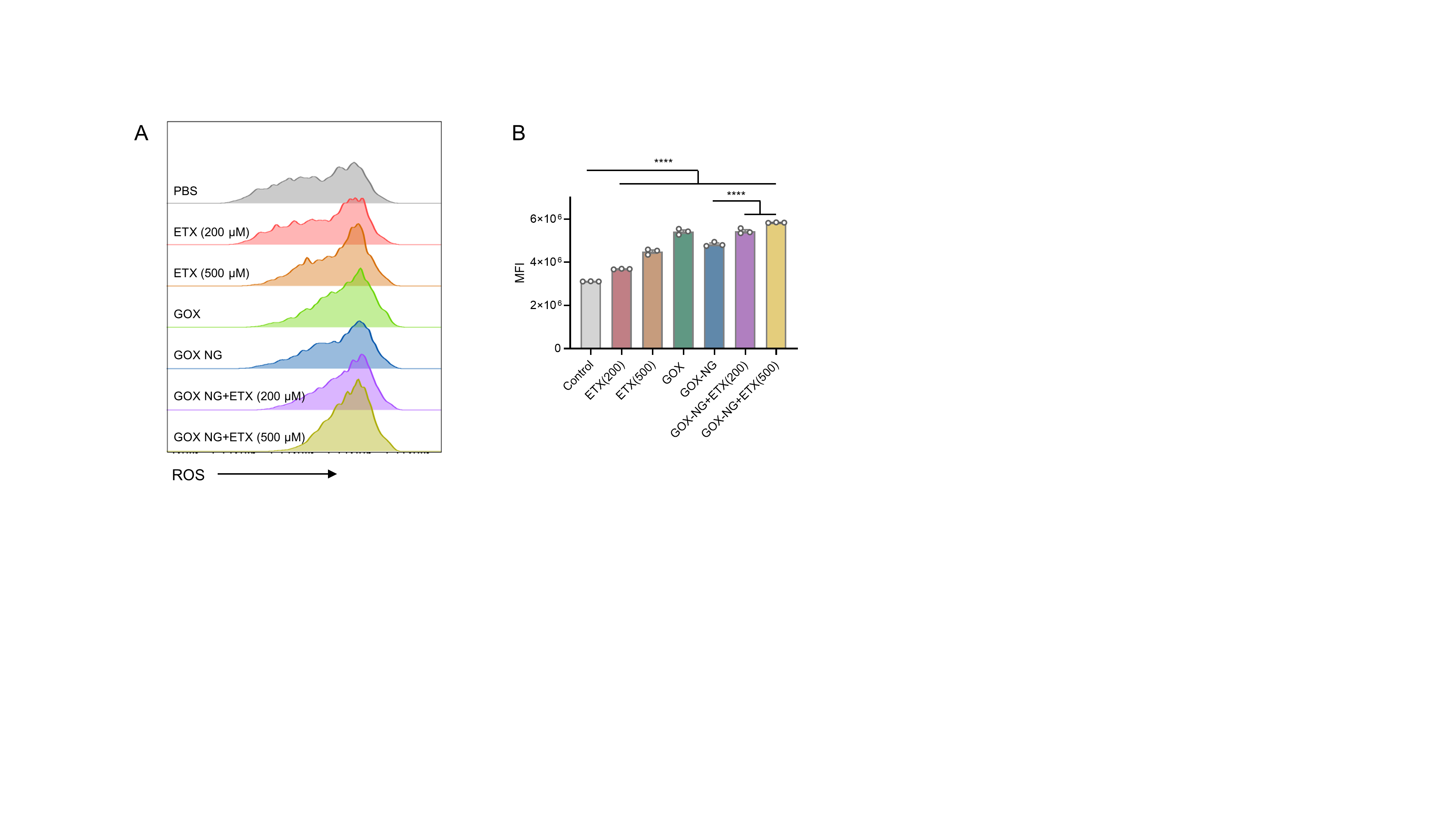


**Figure S23.** 4T1 cells were incubated with different regimens for 24 h, and ROS levels and quantification were measured by flow cytometry (n = 3).


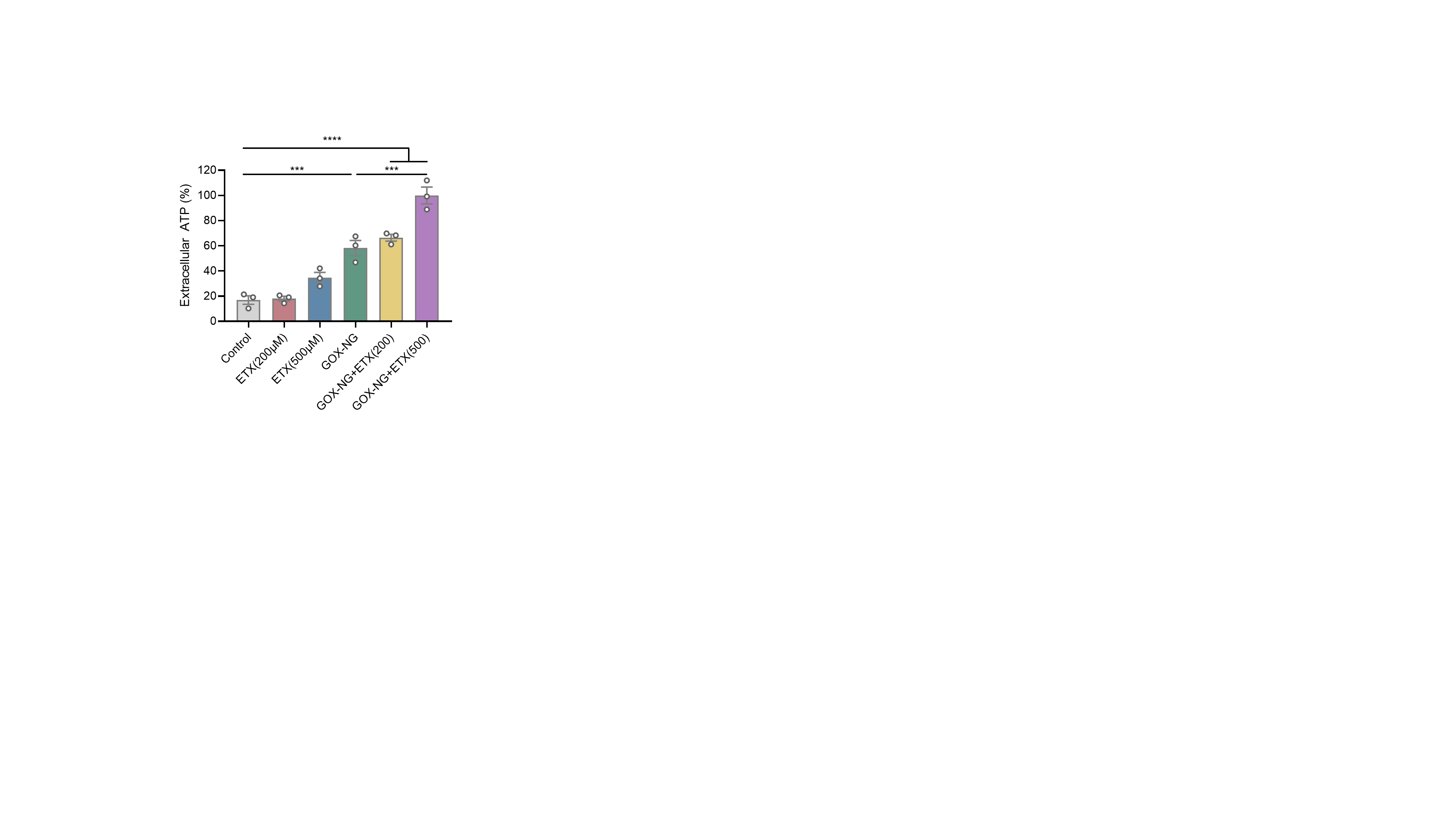


**Figure S24.** Extracellular ATP content of SCC7 cells after incubation with different regimens for 24 h (n = 3).


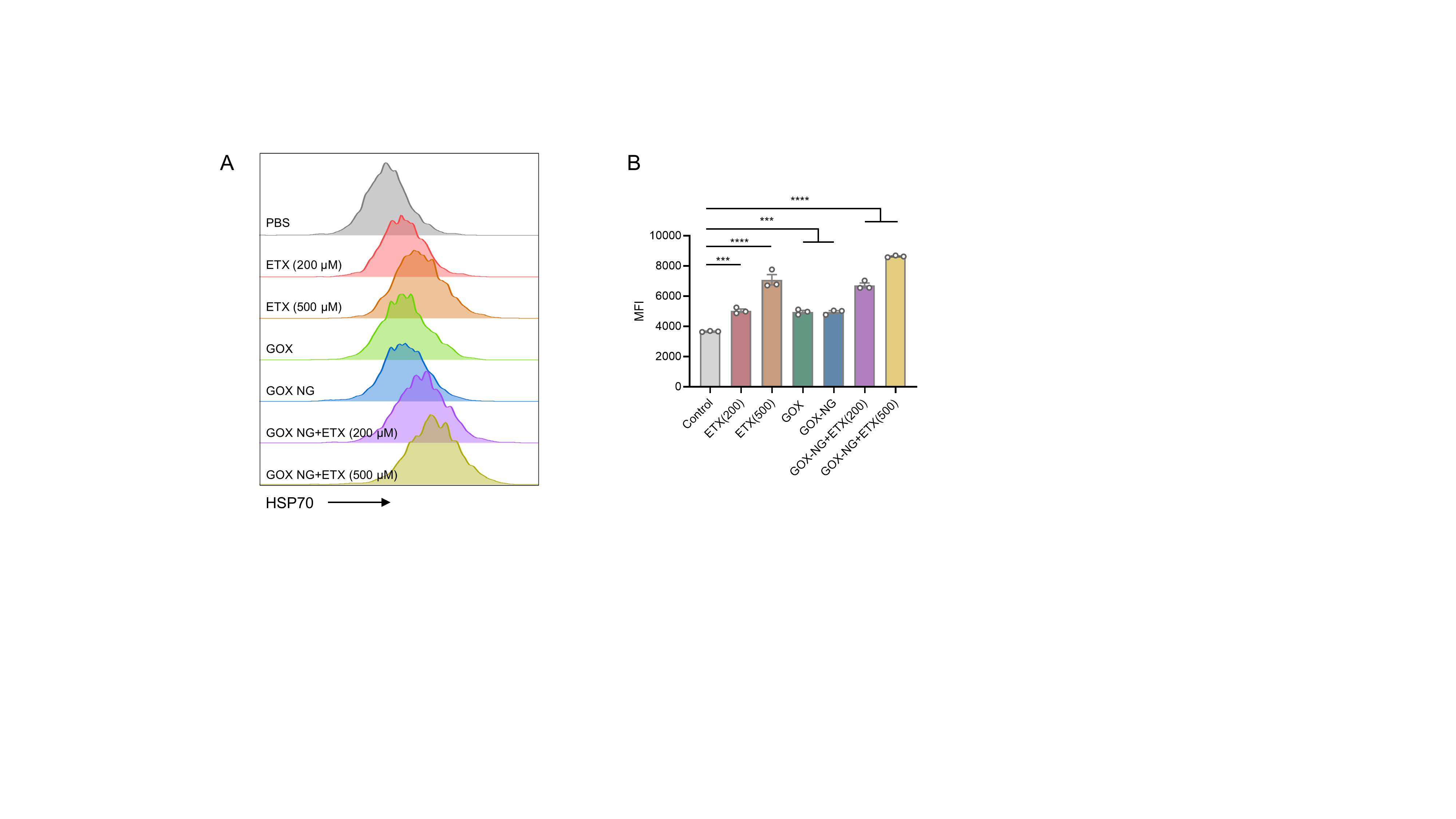


**Figure S25.** Expression of HSP70 in SCC7 cells was measured by flow cytometry after incubation with different regimens for 24 h (n = 3).


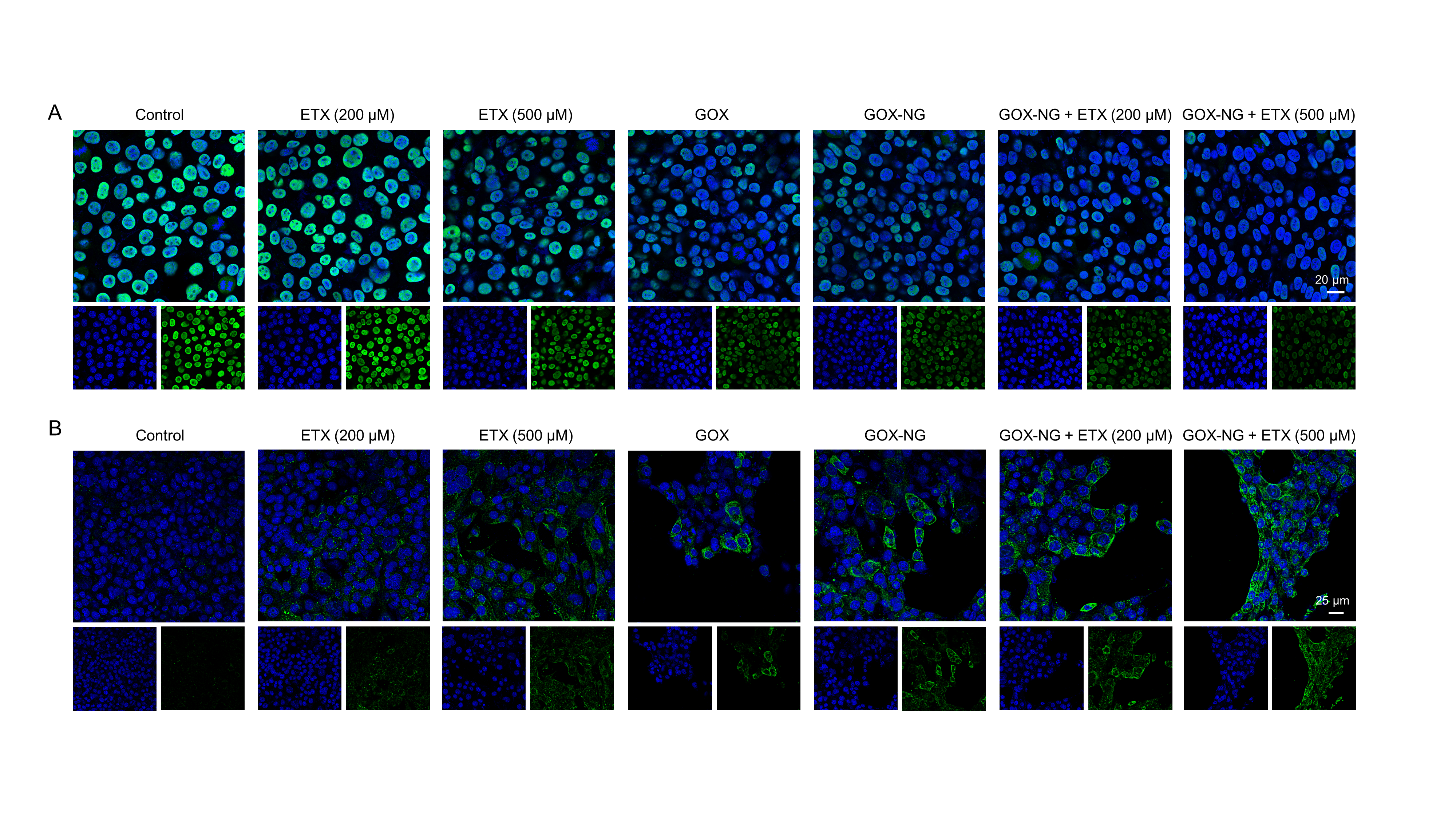


**Figure S26.** Representative CLSM images of HMGB1 (A) and CRT (B) in 4T1 cells after incubation with different regimens for 24 h.


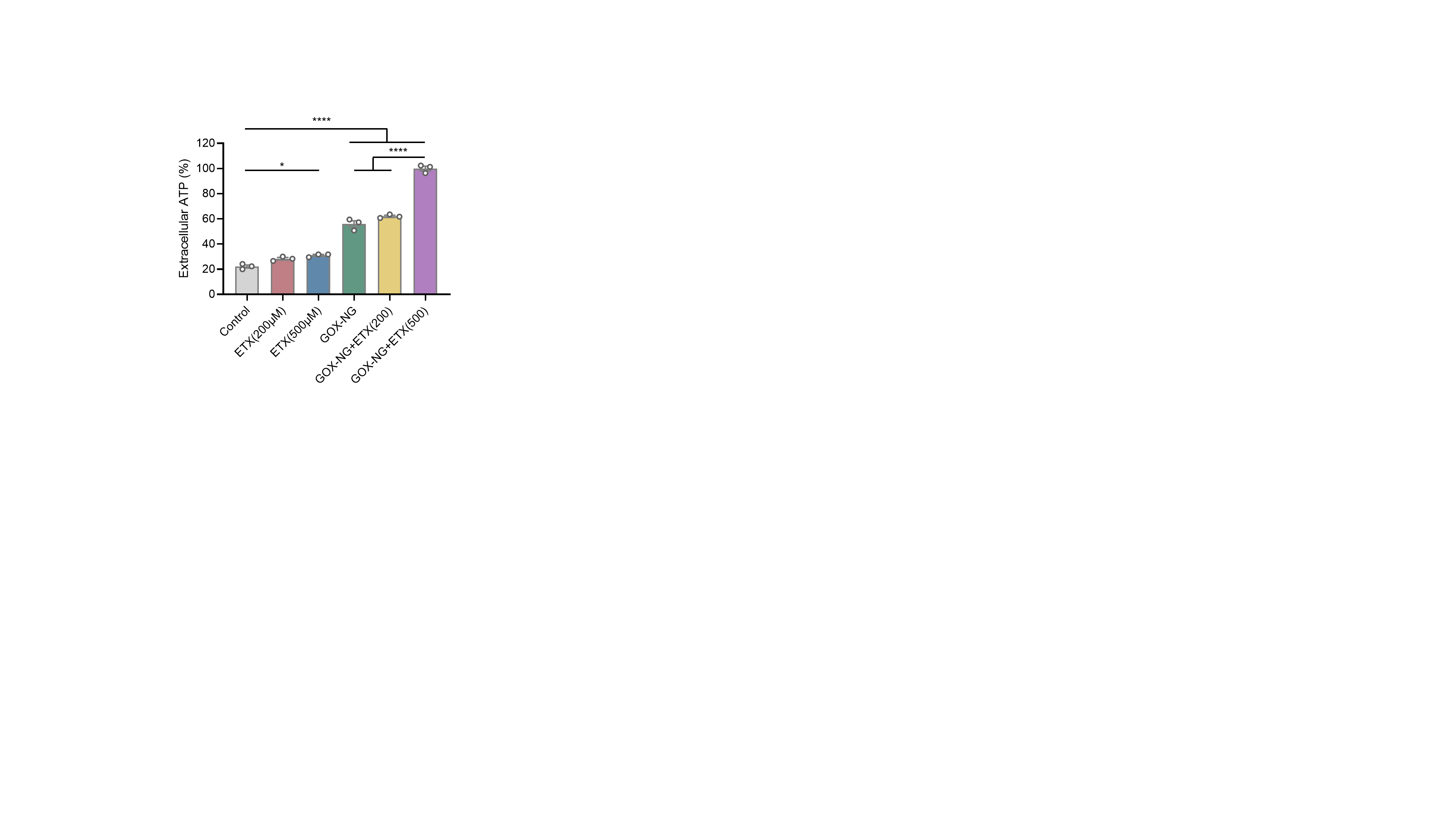


**Figure S27.** Extracellular ATP content of 4T1 cells after incubation with different regimens for 24 h (n = 3).


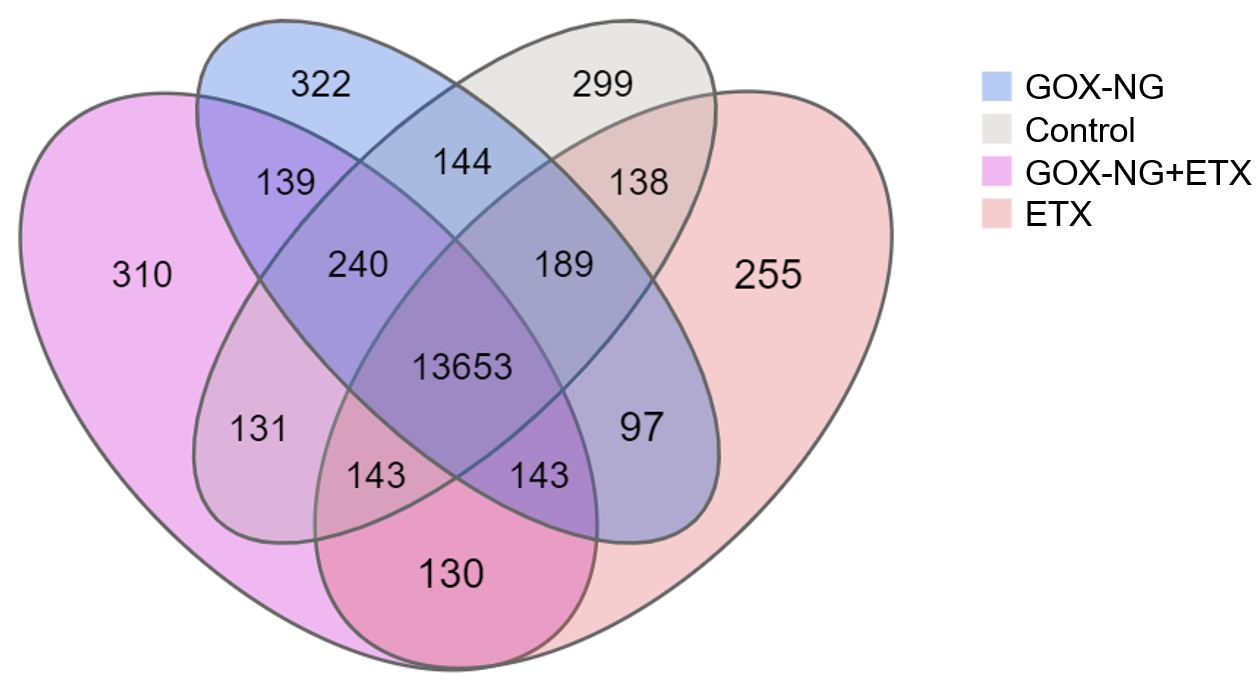


**Figure S28.** Venn diagrams of gene expression levels in different groups.


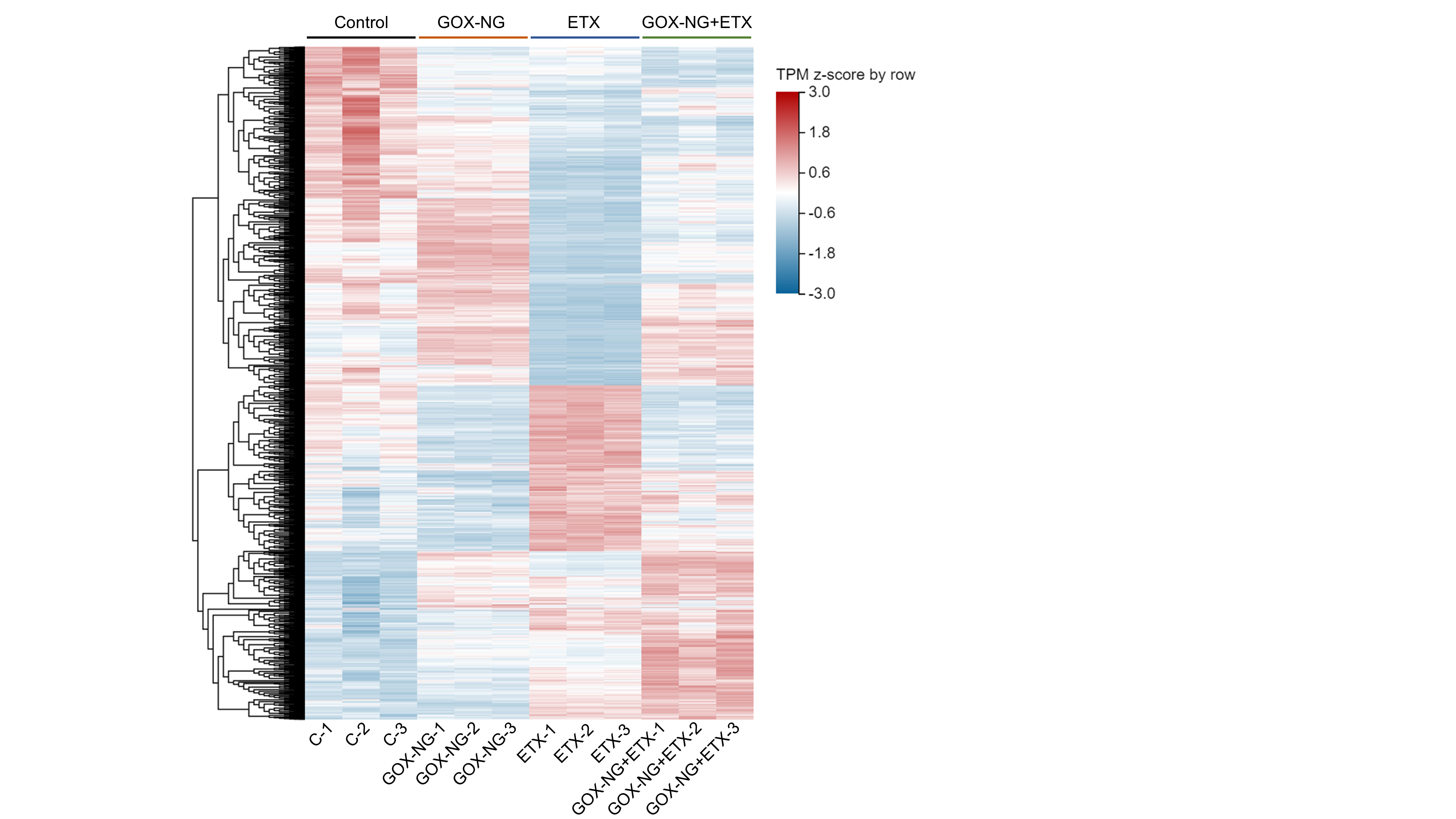


**Figure S29.** All heatmap of expression profiles of DEGs in different groups.


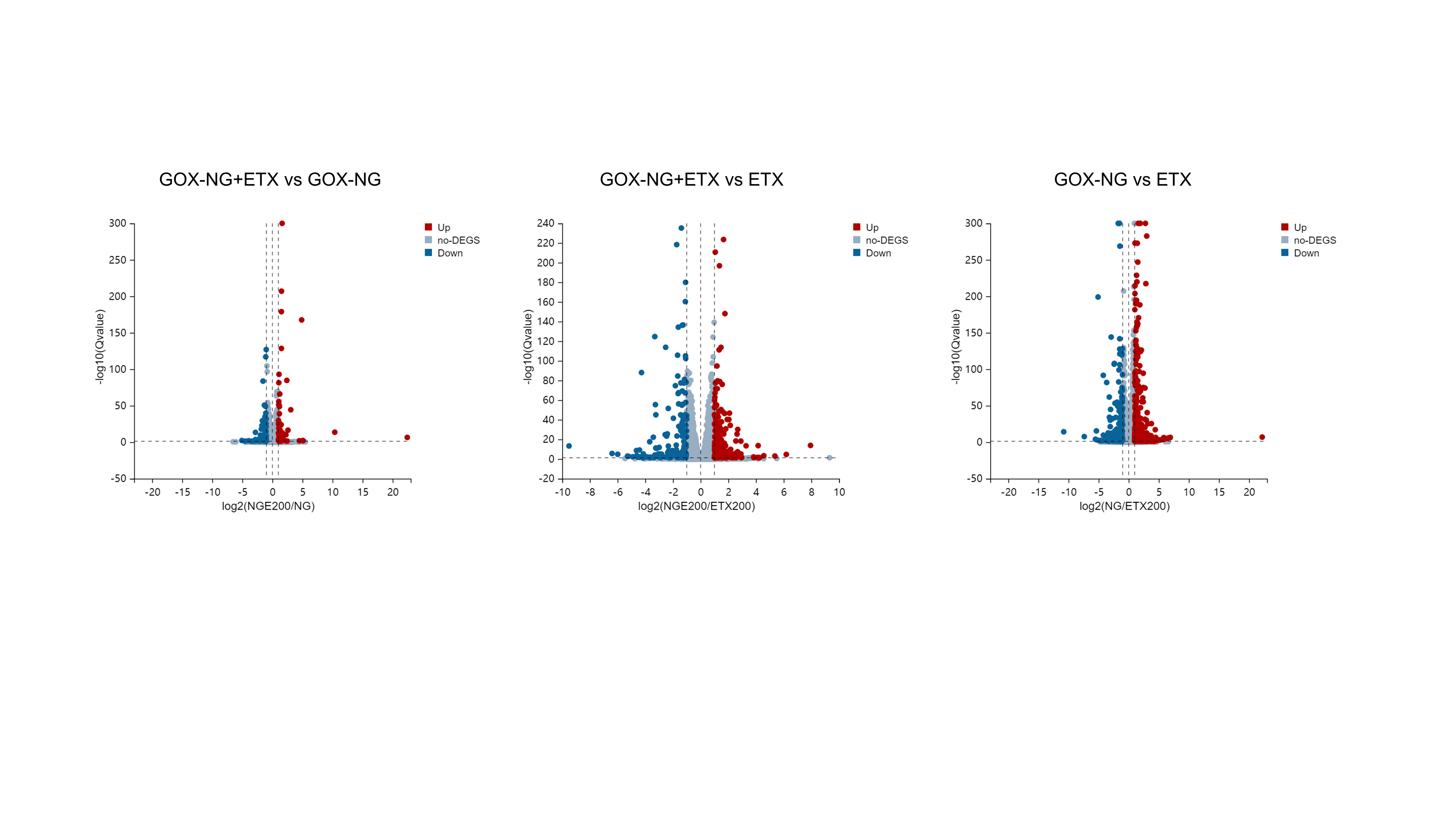


**Figure S30.** Volcano plots displayed the DEGs (GOX-NG+ETX vs GOX-NG, GOX-NG+ETX vs ETX, GOX-NG vs ETX).


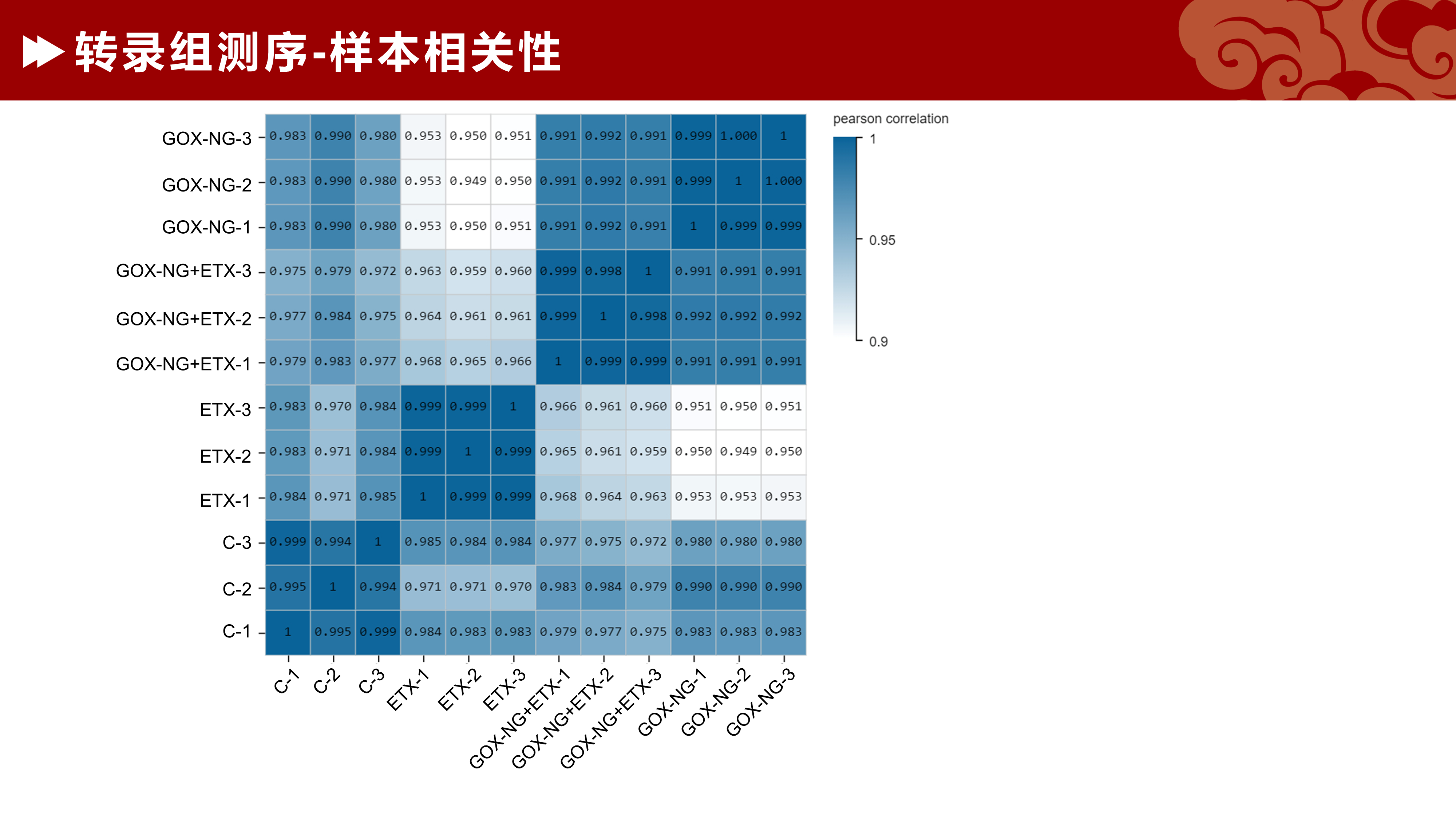


**Figure S31.** Heatmap of the Pearson correlation coefficient of genes in each group.


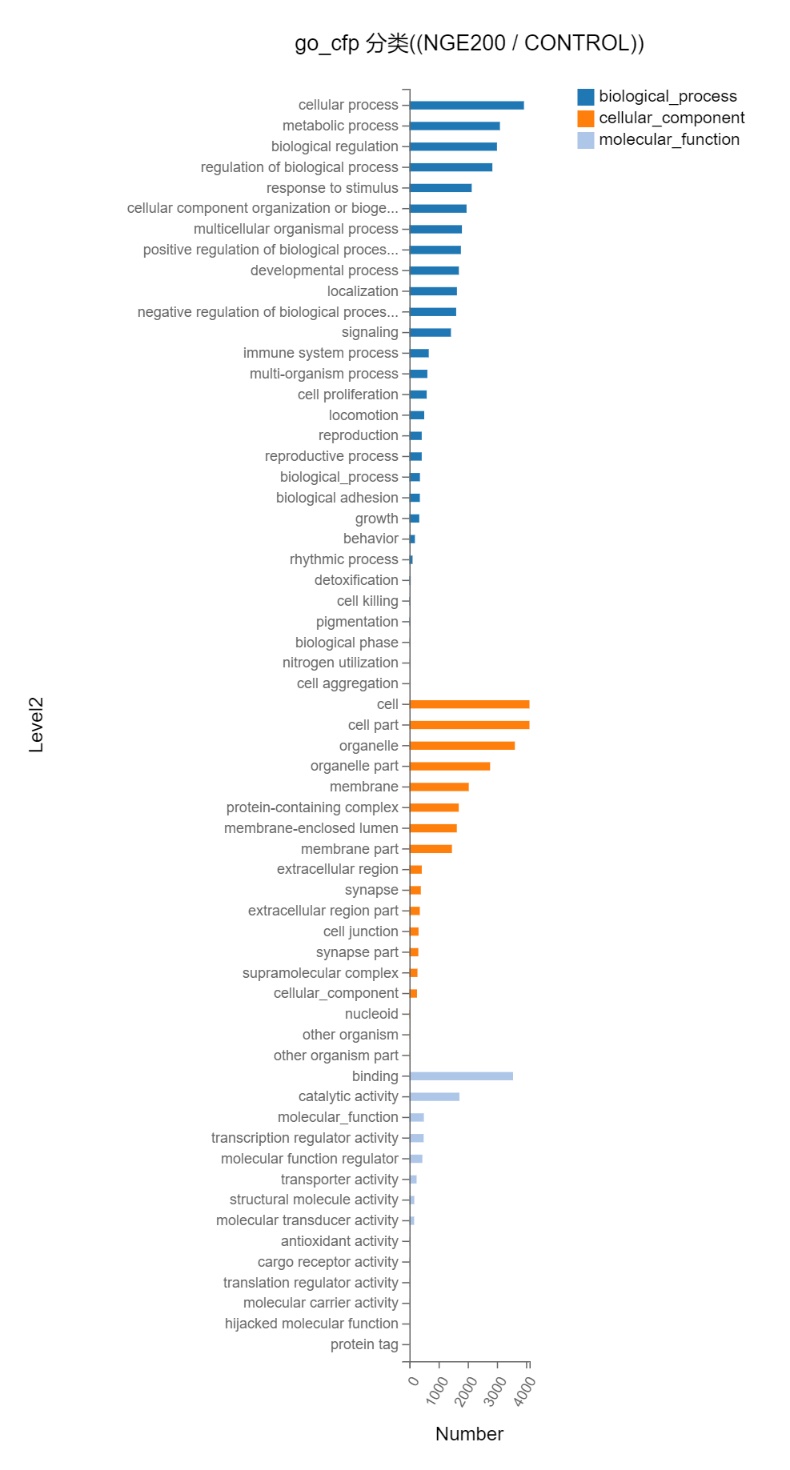


**Figure S32.** GO annotation classification of DEGs (Control vs GOX-NG + ETX).


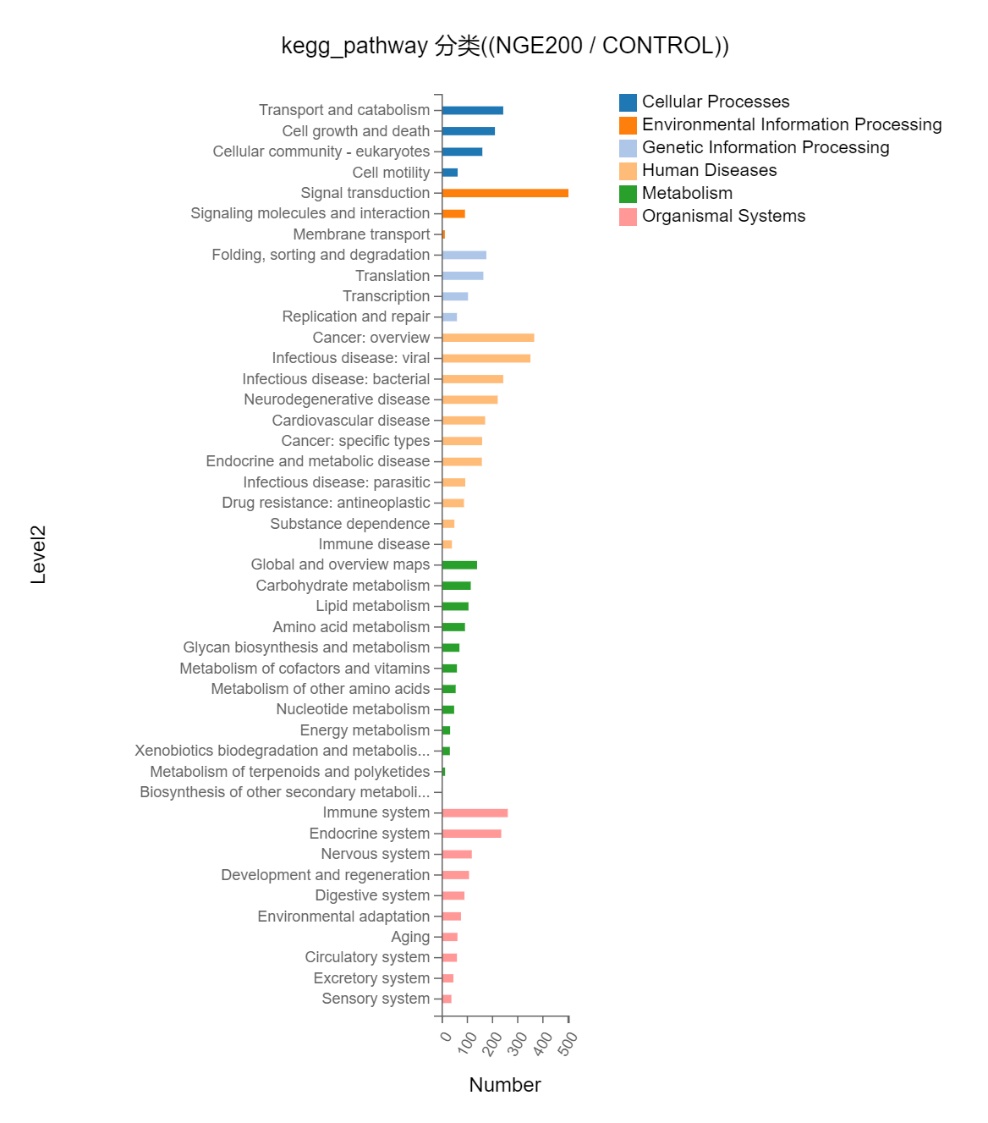


**Figure S33.** KEGG Pathway annotation classification of DEGs (Control vs GOX-NG + ETX).


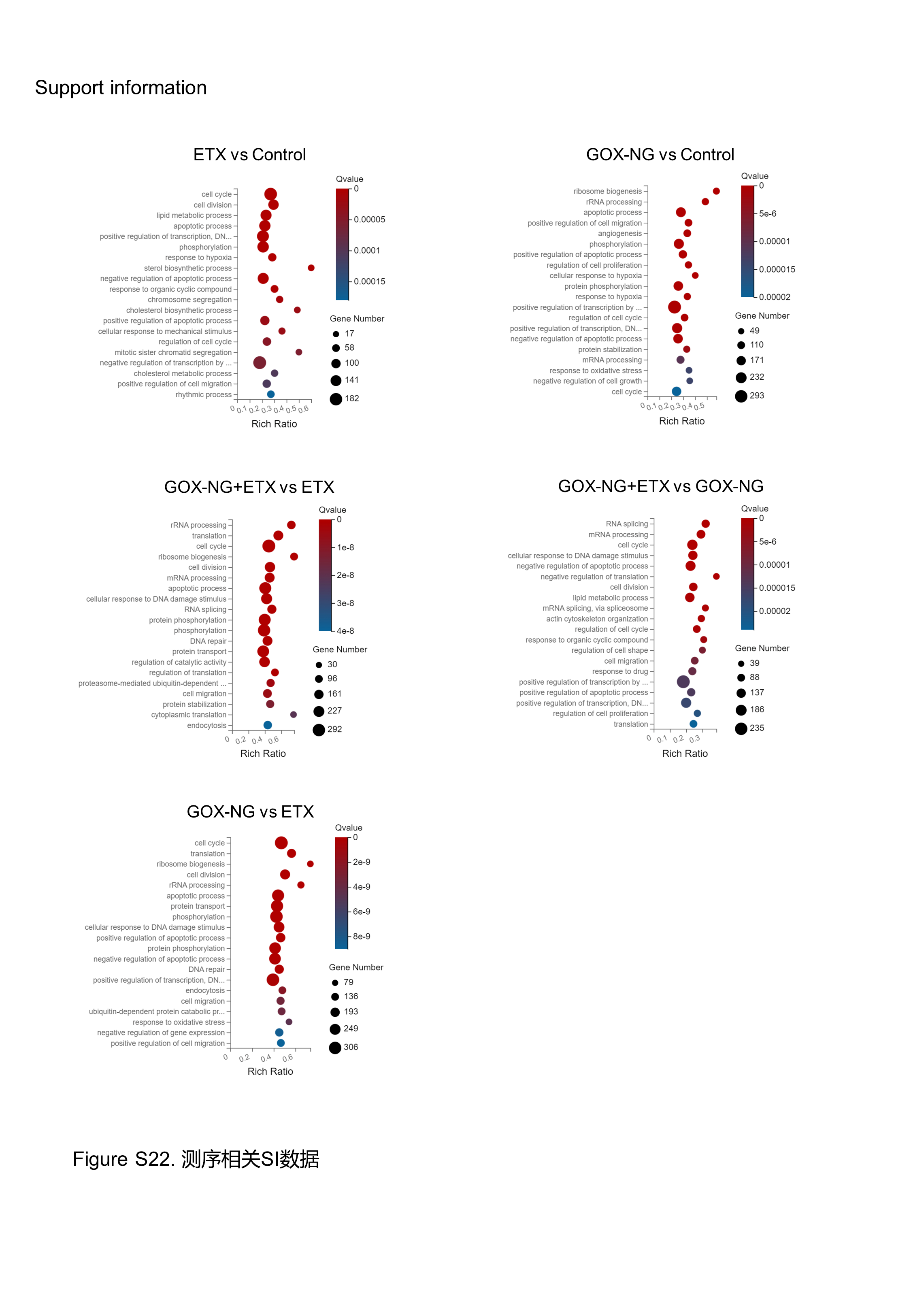


**Figure S34.** GO classiffcation of DEGs (ETX vs Control, GOX-NG vs Control, GOX-NG + ETX vs ETX, GOX-NG + ETX vs GOX-NG, GOX-NG vs ETX).


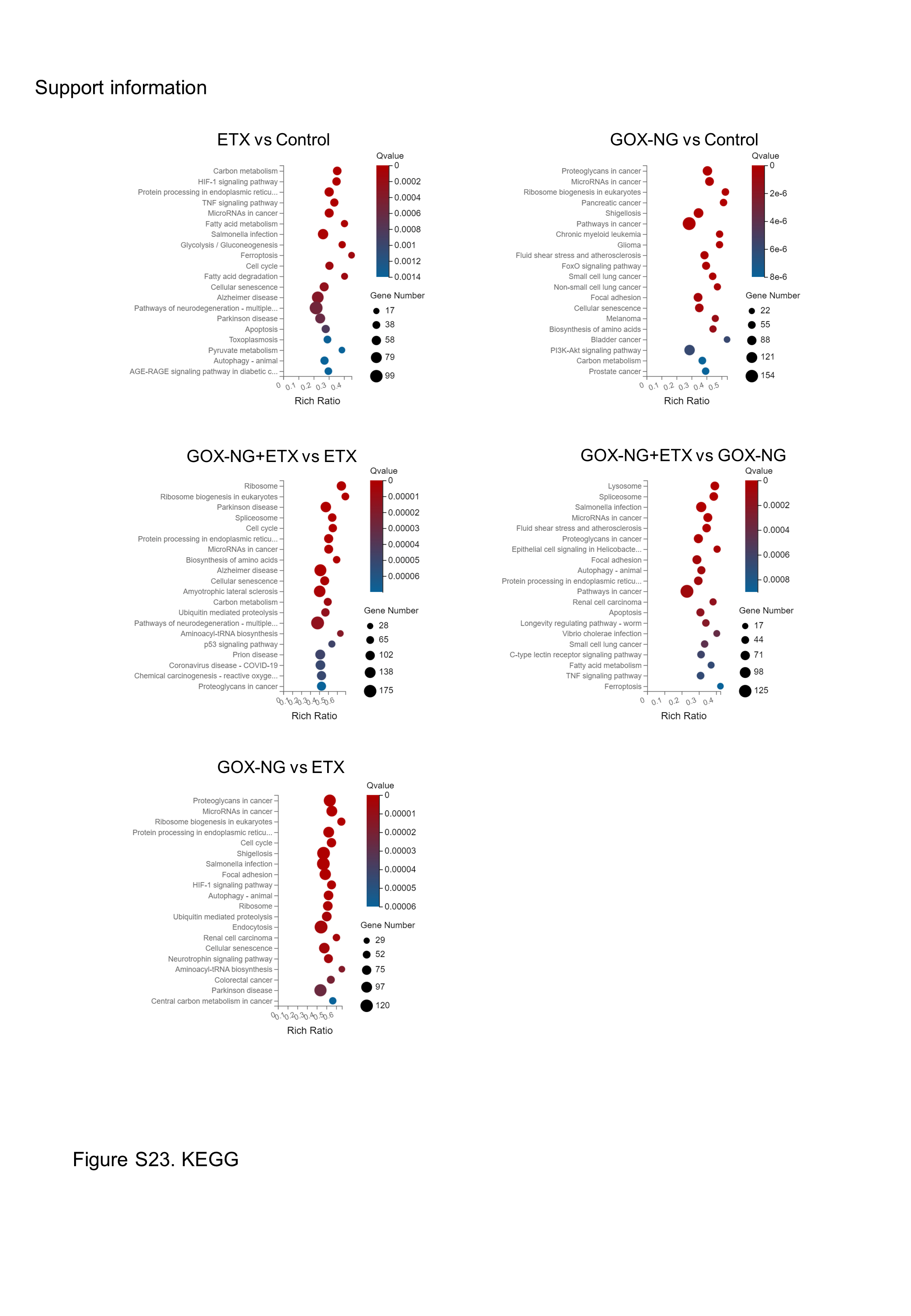


**Figure S35.** KEGG Pathway classiffcation of DEGs (ETX vs Control, GOX-NG vs Control, GOX-NG + ETX vs ETX, GOX-NG + ETX vs GOX-NG, GOX-NG vs ETX).


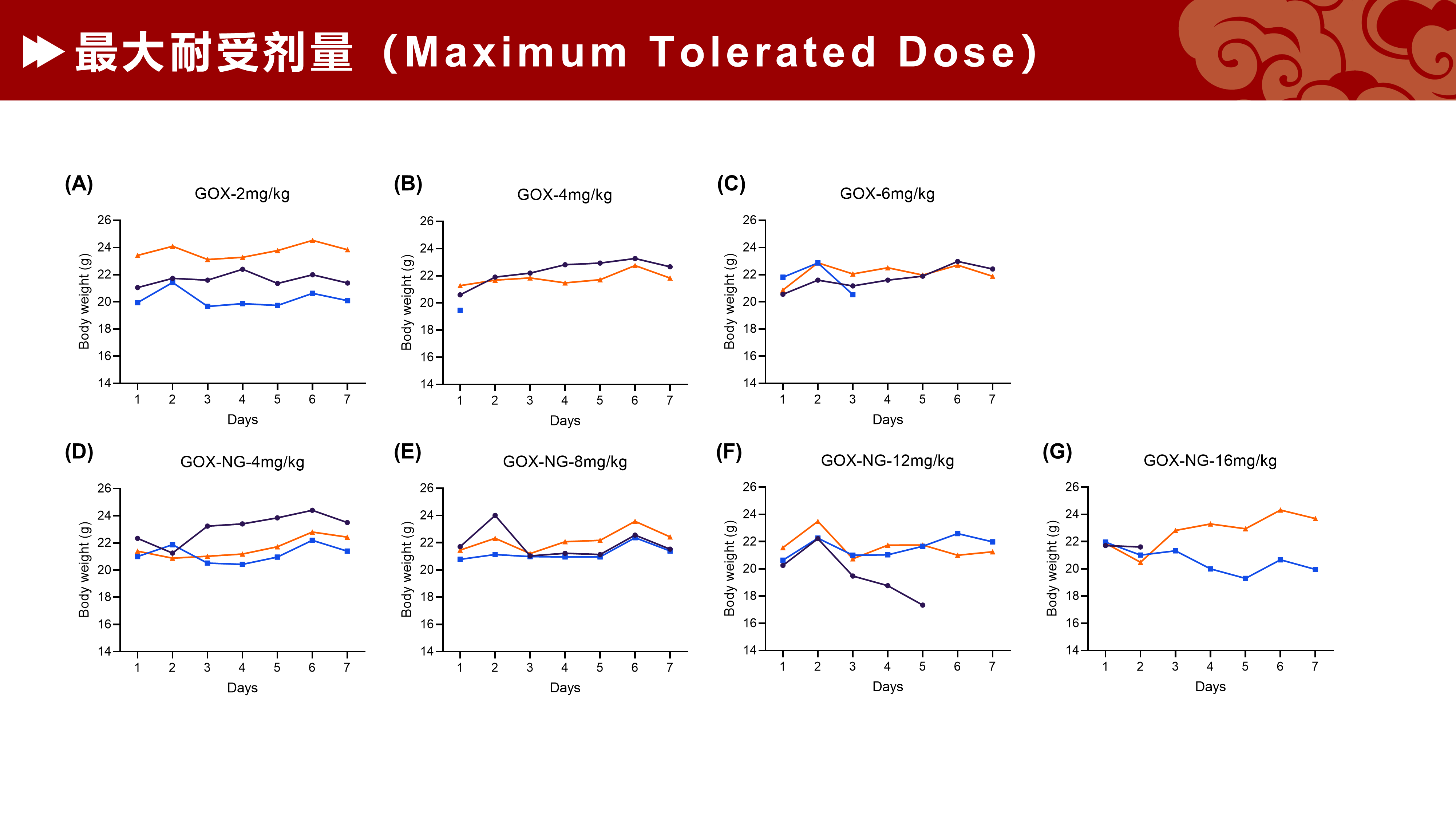


**Figure S36.** After intratumoral injection of GOX or GOX-NG in SCC7 tumor-bearing mice, the body weight of the mice was weighed every day for 7 consecutive days to detect their maximum tolerated dose.


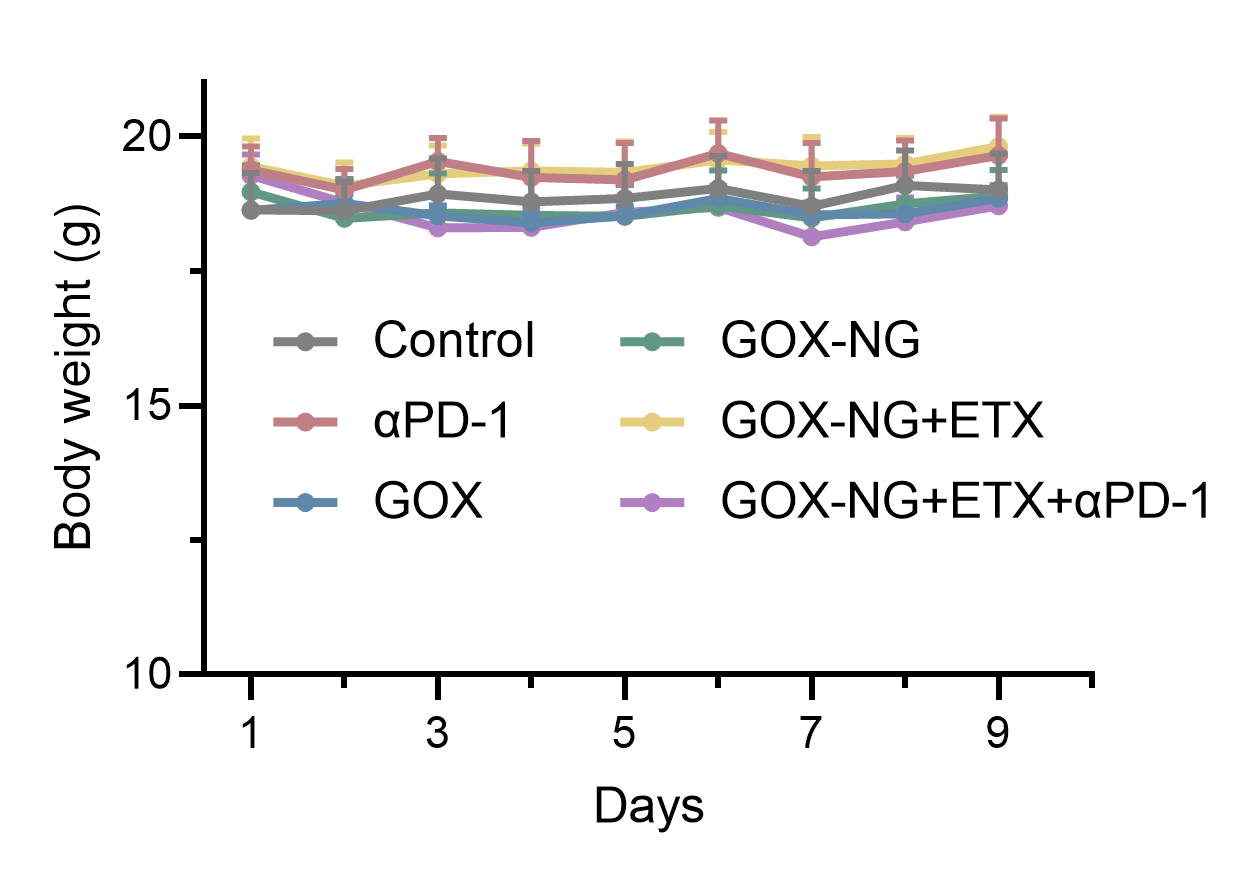


**Figure S37.** Average body weight of mice in different treatment groups during treatment (n = 5).


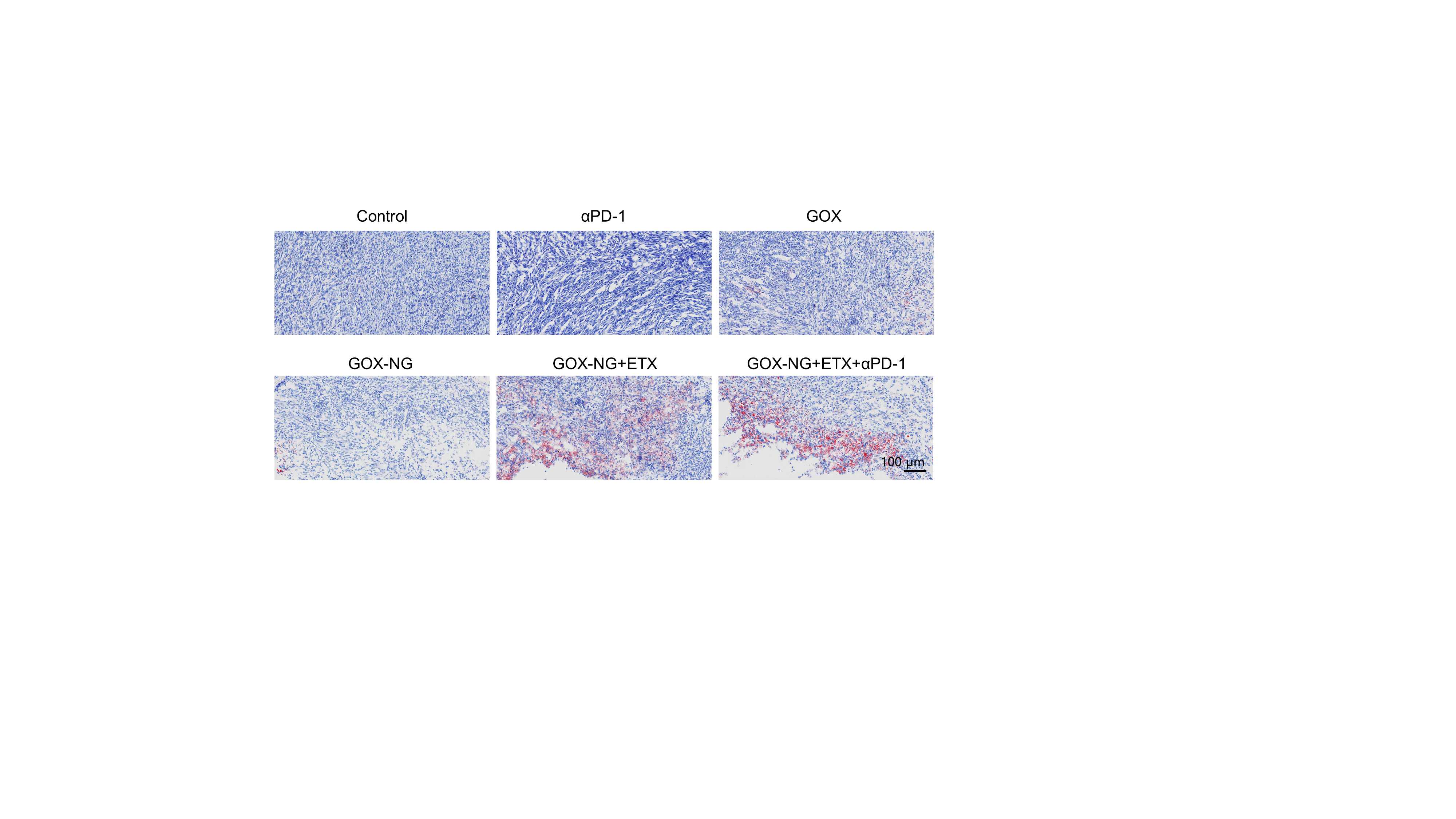


**Figure S38.** Oil red O staining of tumor sections of SCC7 tumor-bearing mice after different anti-tumor treatment regimens. Scale bar, 100 μm.


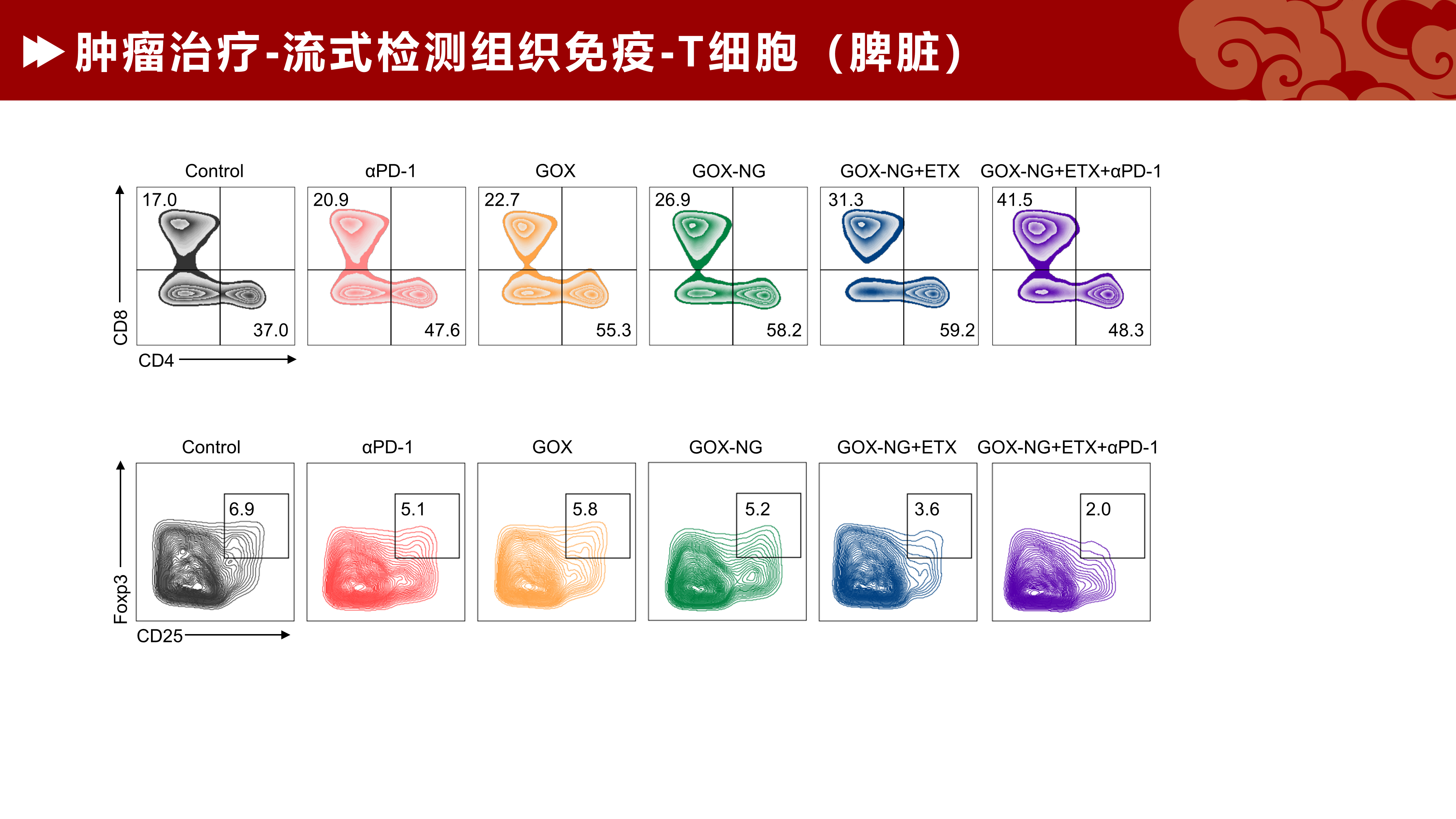


**Figure S39.** Representative flow cytometry plots of CD4^+^ and CD8^+^ T cells in the spleen of SCC7 tumor-bearing mice after different anti-tumor treatment regimens.


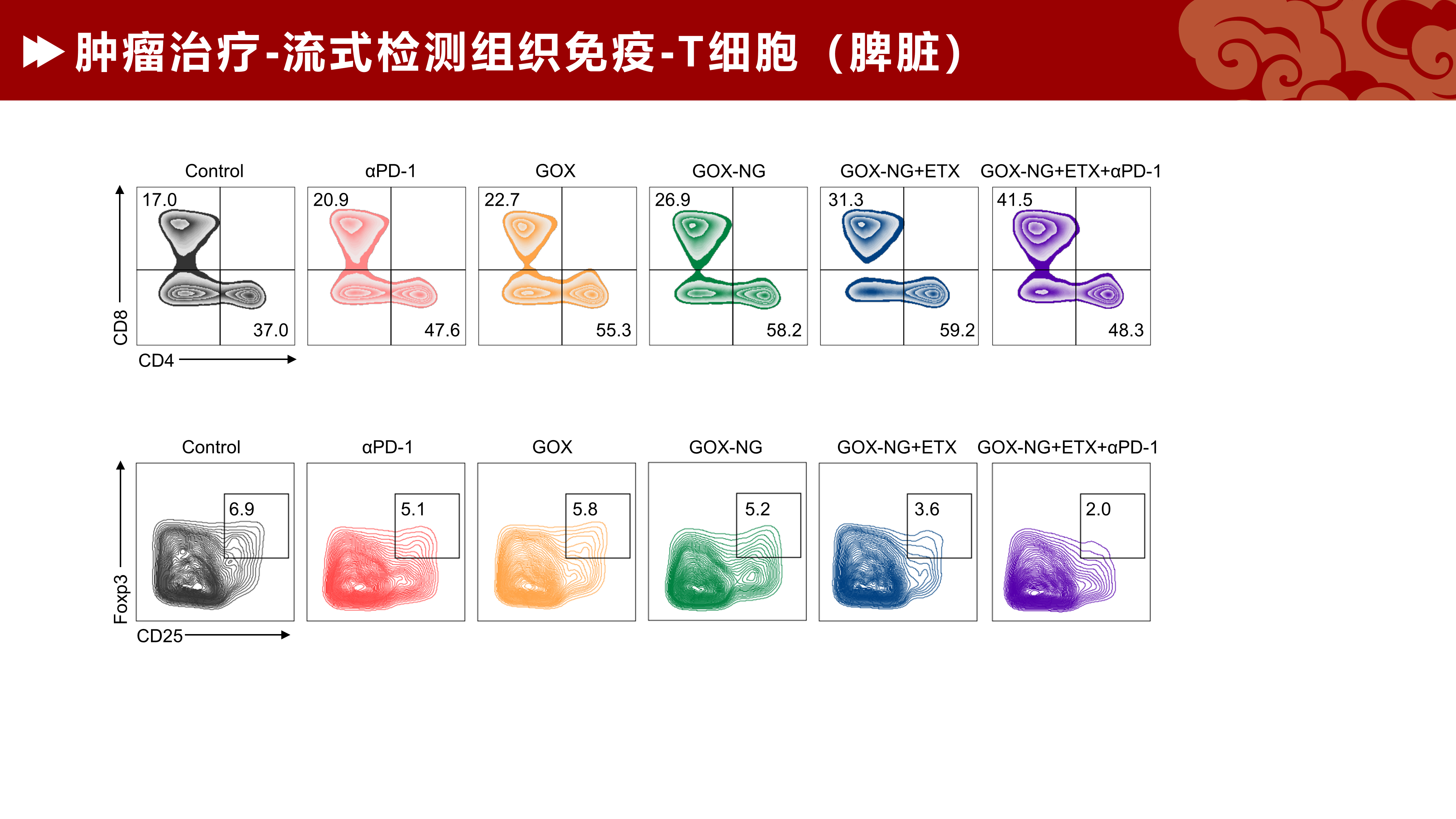


**Figure S40.** Representative flow cytometry plots of Treg cells in the spleen of SCC7 tumor-bearing mice after different anti-tumor treatment regimens.


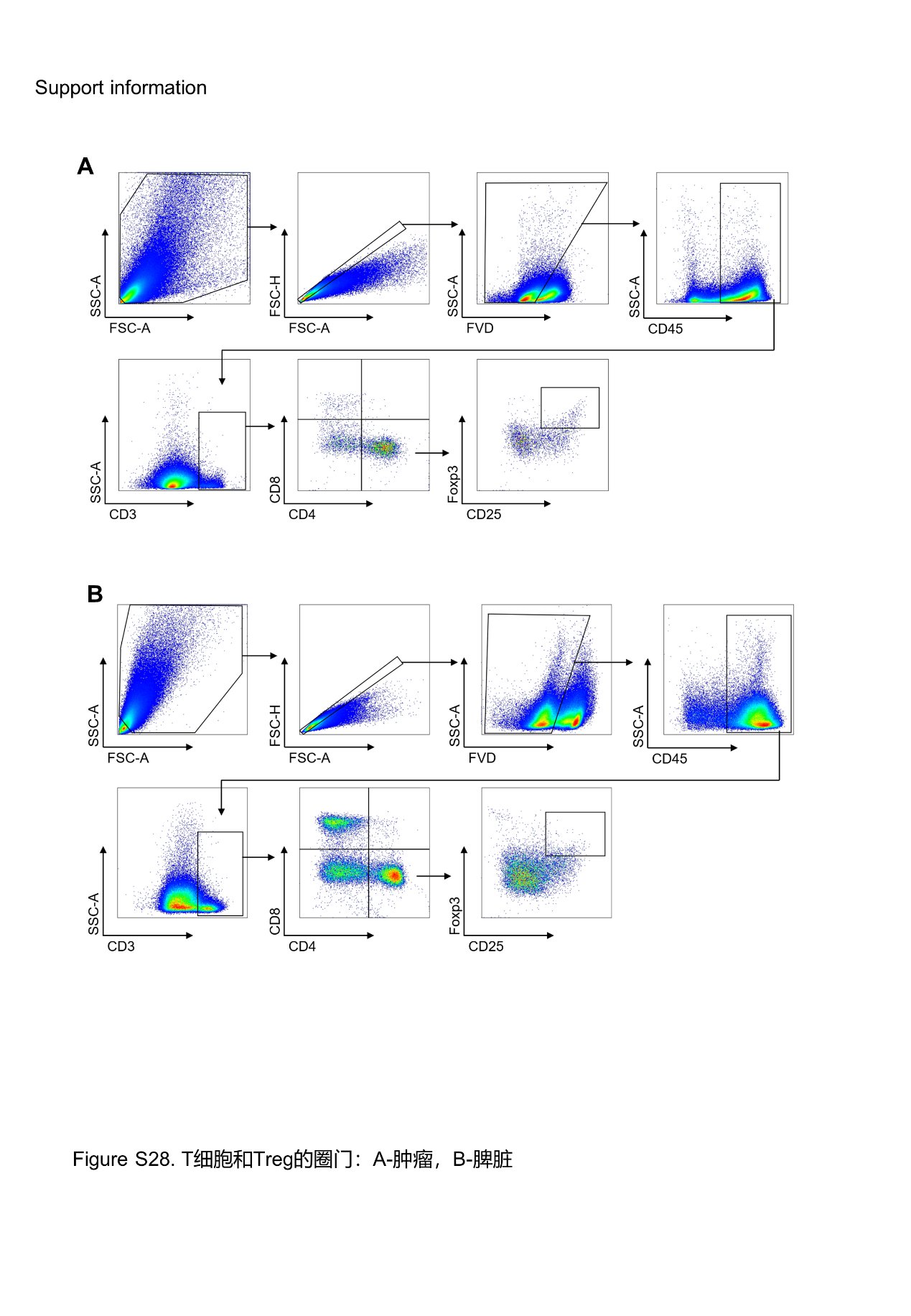


**Figure S41.** Gating strategy of CD4^+^ T cells, CD8^+^ T cells and Treg in tumor (A) or spleen (B).


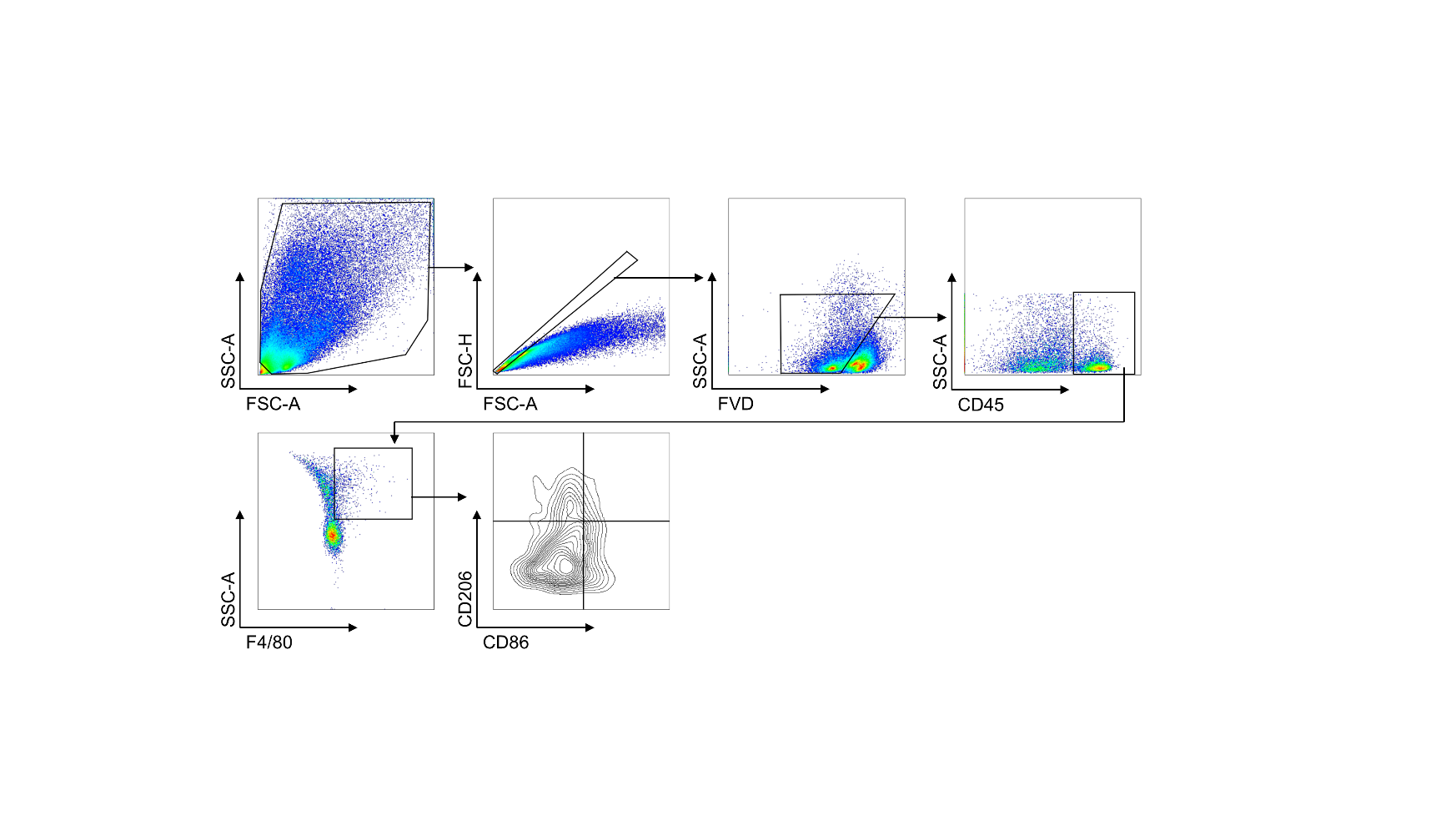


**Figure S42.** Gating strategy of macrophages.


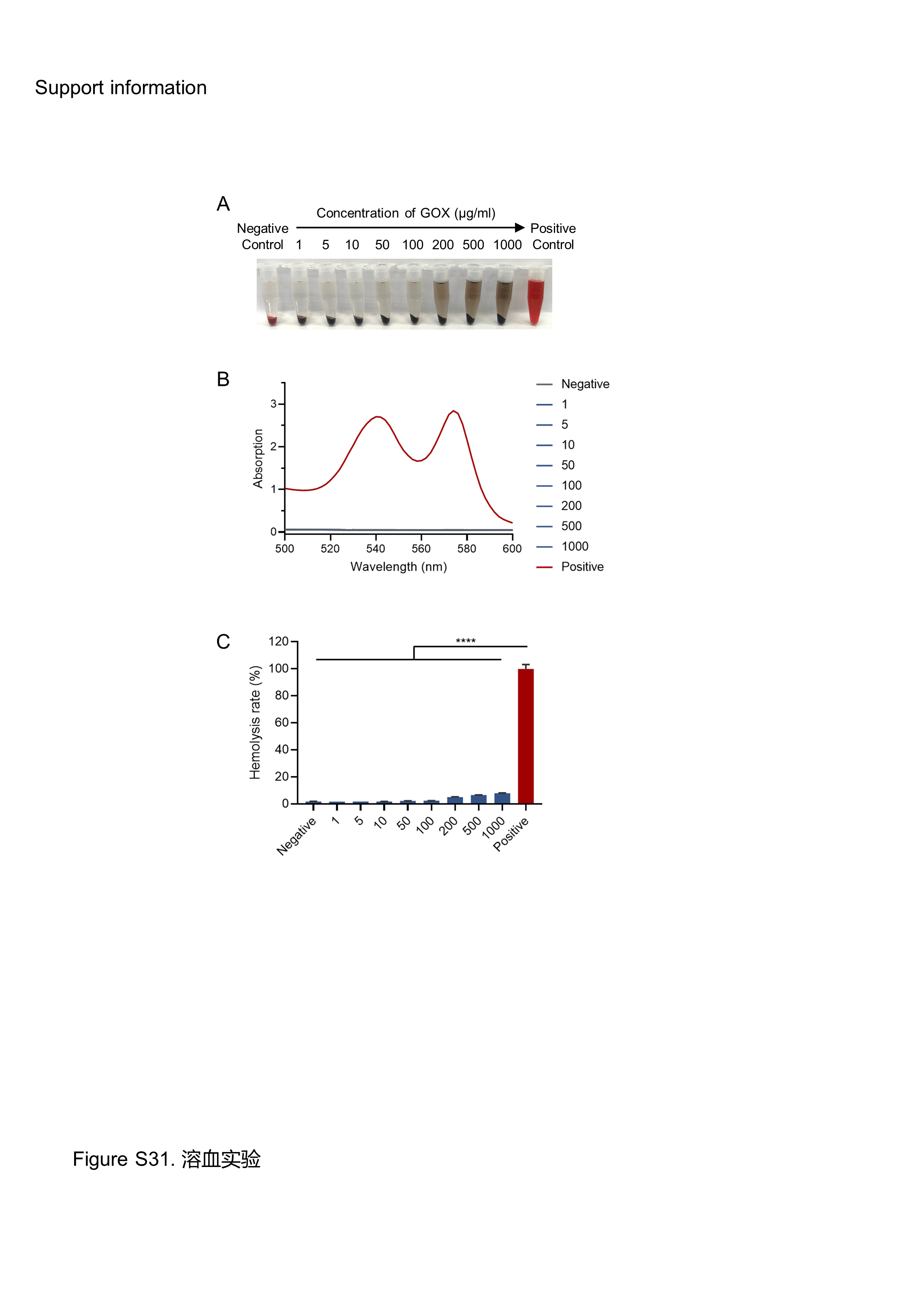


**Figure S43.** Hemolysis test. (A) Images of GOX-NG at different concentrations incubated with red blood cells for 1 hour after centrifugation (the negative control was normal saline and the positive control was water). (B) UV spectra of the supernatant. (C) Quantitative ultraviolet absorption and hemolysis rate at the characteristic peak (540nm). Data represent the means ± SEM, n = 3.


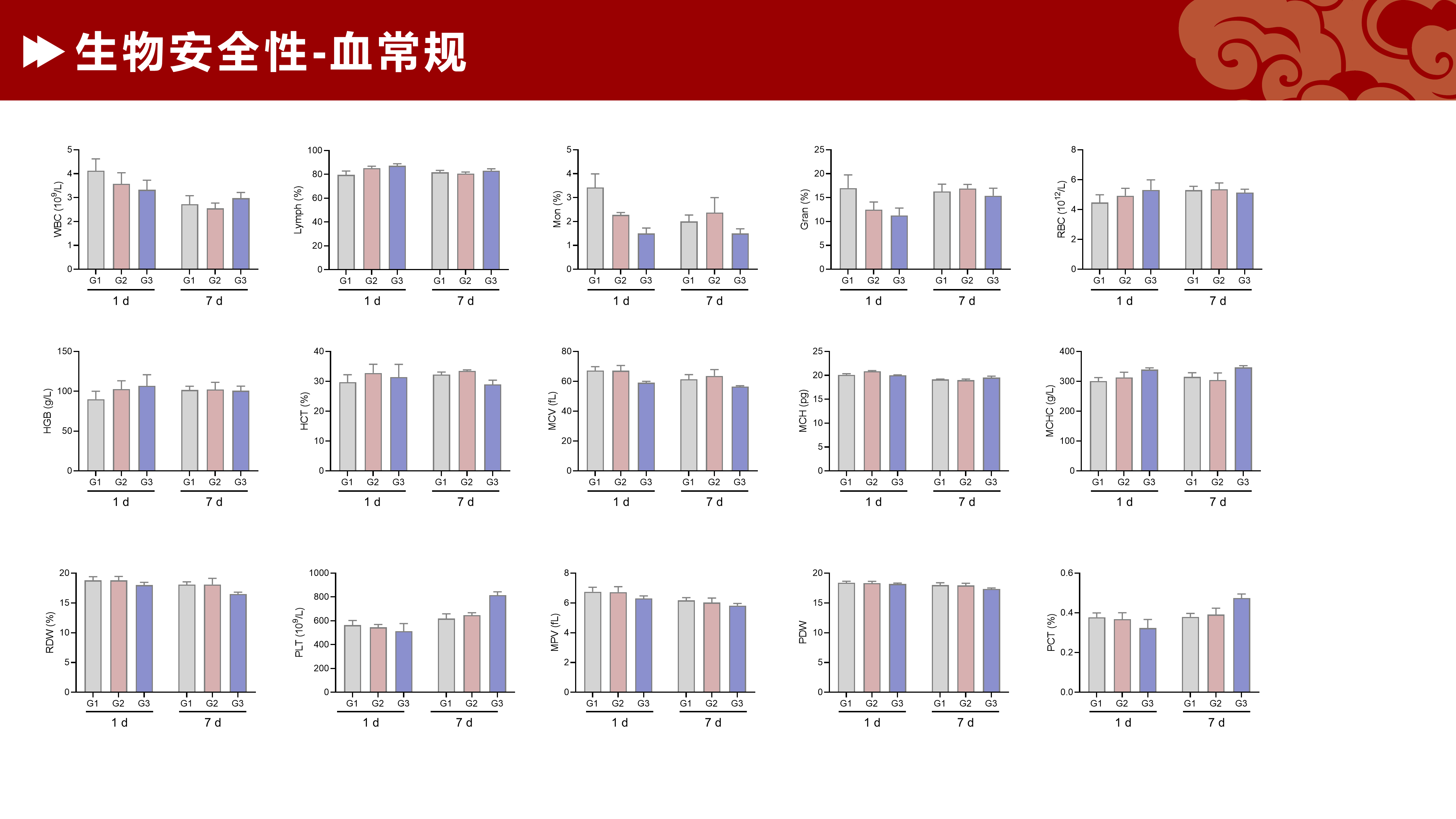


**Figure S44.** After intratumoral injection of the drug (G1-Control, G2-GOX, G3-GOX-NG) in SCC7 tumor-bearing mice, whole blood was collected on the 1st and 7th days for blood routine test. Data represent the means ± SEM, n = 4.


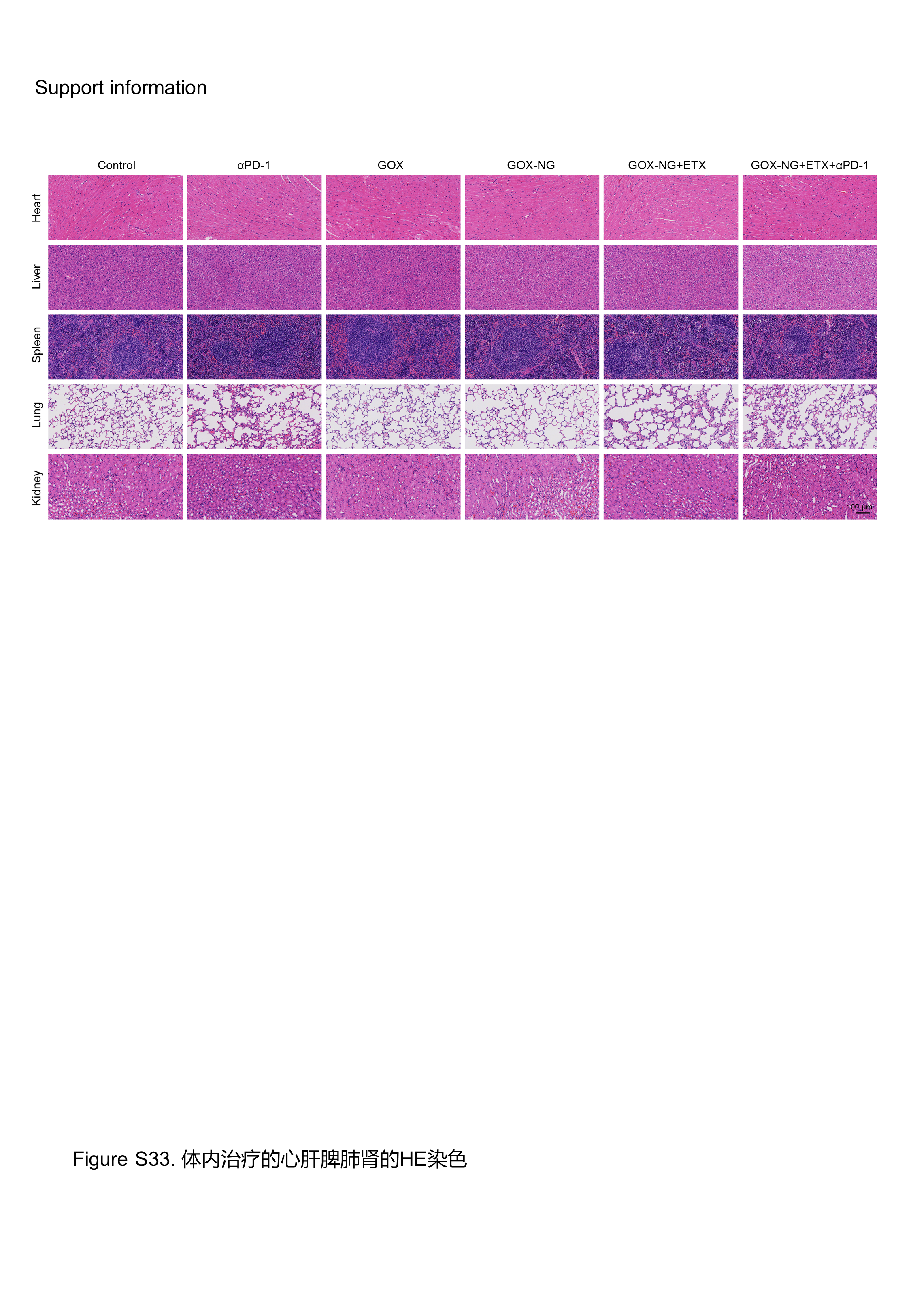


**Figure S45.** HE staining of representative sections of organs (heart, liver, spleen, lung, and kidney) of SCC7 tumor-bearing mice after different anti-tumor treatment regimens. Scale bar, 100 μm.

**Table S1.** Primer sequences.

| Gene | Primer sequences (F, forward; R, reverse) |
| --- | --- |
| CD206 | F: 5’- CGGAATTTCTGGGATTCAGCTTC-3’  R: 5’- CTCTGTTCAGCTATTGGACGC -3’ |
| IL-10 | F: 5’- GCTGGACAACATACTGCTAACC-3’  R: 5’- ATTTCCGATAAGGCTTGGCAA -3’ |
| TGF-β | F: 5’- CAGTACAGCAAGGTCCTTGC-3’  R: 5’- ACGTAGTAGACGATGGGCAG-3’ |
| TNF-α | F: 5’- TGTCTCAGCCTCTTCTCATT-3’  R: 5’- TGATCTGAGTGTGAGGGTCT -3’ |
| IL-6 | F: 5’- GTCCTTCCTACCCCAATTTCCA-3’  R: 5’- TAACGCACTAGGTTTGCCGA -3’ |
| β-actin | F: 5’- GGCTGTATTCCCCTCCATCG-3’  R: 5’- CCAGTTGGTAACAATGCCATGT-3’ |

**Table S2**. Antibodies for FACS.

| Antibody | Dilution ratio |  |  |
| --- | --- | --- | --- |
| Live/Dead | 1:1000 | Thermo Scientific | eFluor 506 |
| anti-CD45 | 1:500 | Biolegend | PE-Cy5 |
| anti-CD3 | 1:300 | Biolegend | APC |
| anti-CD4 | 1:300 | BD Biosciences | APC-Cy7 |
| anti-CD8a | 1:600 | Biolegend | Pacific Blue |
| anti-CD25 | 1:200 | Biolegend | PE |
| anti-FoxP3 | 1:200 | BD Biosciences | PerCP-Cy5.5 |
| anti-CD11b | 1:300 | TONBO Biosciences | FITC |
| anti-F4/80 | 1:300 | TONBO Biosciences | PerCP-Cy5.5 |
| anti-CD86 | 1:200 | Biolegend | PE |
| anti-CD206 | 1:200 | Biolegend | APC |
| anti-F4/80 | 1:300 | Biolegend | PE |
| anti-CD163 | 1:200 | Biolegend | PE-Cy7 |
